# Consensus Formation and Change are Enhanced by Neutrality

**DOI:** 10.1002/advs.202512301

**Published:** 2026-03-22

**Authors:** Andrei Sontag, Janina A. Hoffmann, Tim Rogers, Christian A. Yates

**Affiliations:** ^1^ Department of Mathematical Sciences University of Bath Bath UK; ^2^ Department of Mathematics University College London London UK; ^3^ Department of Psychology University of Bath Bath UK

**Keywords:** collective behaviour, consensus formation, decision making, individual‐based model, stochastic switching

## Abstract

Effective collective decision‐making in human and animal groups requires robust mechanisms for consensus formation and change, typically via feedback loops in which individuals adapt their behavior and opinions based on their perception of others. Such processes have been observed in the onset of motion in insect swarms and are believed to manifest across scales from nucleosomes to entire societies. However, levels of participation can be highly variable over time, with individuals sometimes adopting neutral positions such as moving to the back of a group or abstaining from a vote. Here, we present a new theoretical and experimental analysis showing that neutrality has two important and hitherto unreported benefits to collective decision‐making. First, it enables the robust formation of consensus in groups of individuals applying simple linear reasoning, updating their stance after consideration of at most one other individual at a time. Second, we find that neutral actors can facilitate efficient consensus change by reducing the effective population size during transitions. These findings are derived from a new general mathematical model of collective binary decision problems and validated against experiments with insect and human populations. Our results provide a parsimonious explanation of how groups of animals and humans quickly reach and overturn consensus, suggesting efficient solutions to collective decision‐making problems.

## Graphical abstract

Neutral agents are shown to enhance both the formation and overturning of consensus in collective decision‐making. A general mathematical model and experiments with locusts and humans reveal that neutrality enables robust consensus via simple interactions and accelerates consensus change by reducing effective population size. These findings offer a parsimonious explanation for adaptive group decisions.

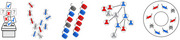

Consensus formation is core to the structures and processes of both human [1] and animal [2] societies. Honeybees [9] and ants [10, 11], demonstrate sophisticated mechanisms for choosing between potential nesting sites, and many social insects are known to exhibit complex collective decision‐making abilities when tasked with choosing between competing food sources [12, 13]. The formation and change of consensus is known to play a role in foraging efficiency [14], migration [15], and synchronization within animal populations. Ultimately, the later stages of human evolution have been characterized by sociality and collective coordination [16]. With the emergence of complex societies, consensus decision‐making strategies have developed to encompass nuanced tasks, including the resolution of social and ethical dilemmas [17], corporate strategy [18], and the election of representatives as part of the democratic process [6].

The theory surrounding consensus formation is rich and diverse. Heterogeneous opinion dynamics models have been developed for social network group decision‐making, incorporating stubbornness and evolving social ties to study how consensus emerges in large, distributed teams [19]. Computational and kinetic‐theory approaches have been applied to model consensus dynamics in financial markets [20] and artificial multi‐agent systems [21], underscoring the universality of these principles across broad domains. On a grander scale, interdisciplinary syntheses argue for unified models of consensus formation that bridge social, ecological, and technological systems to tackle global challenges [22].

Recent theoretical models have extended consensus formation to diverse application domains. Enhanced consensus‐reaching processes in large‐scale social networks tackle minority opinion management and non‐cooperative behaviors [23]. Entropy‐based negotiation algorithms enable robust consensus in artificial swarms under uncertainty [24]. Physics‐based models of biologically inspired systems reveal how quality‐sensitive agents balance speed and accuracy in consensus formation, echoing mechanisms observed in honeybee colonies [25].

Today, the limitations of our ability to gather and synthesize facts and opinions from members of geographically distributed groups presents a key challenge to social and economic progress. Iterative decision‐making protocols have been proposed as a possible route forward. The Delphi method, originating in the cold war [3] but recently popularized in contributions to public health policy [26], aims to integrate expert opinion through iterated rounds of anonymous contribution, evaluation, and voting. Betting markets, and financial markets more generally, can also be framed as an iterative game. Participants repeatedly engage with the market whilst observing a summary of their co‐participants' actions in the form of the market odds, which are in turn updated based on participants' actions, creating an iterative feedback loop [27]. In a political context, opinion polls provide voters with noisy subsamples of the intentions of the electorate, which may inform their own decision, particularly if voting tactically [28].

Experimental studies of consensus formation typically aim to distil the problem to the simple and controllable form of a binary choice problem in which a group must collectively select between one of two options. Much of the corresponding theoretical literature has been inspired by physics‐based approaches  [29, 30], which frame this scenario as a competition between opposing factions of so‐called ‘voters'  [31], attempting to recruit each other to their preferred option of two alternative ‘opinions’, here denoted X and Y. Voters may change their own opinions based on the opinions of others. Recent studies of these models incorporating higher‐order interactions on hypergraphs and studying dynamics in simplicial complexes, showing that group‐level influence and adaptive topology can accelerate consensus or create metastable states, enriching the dynamics beyond pairwise interactions [32, 33, 34, 35].

Writing z for the difference between the frequency of X and Y voters, and ignoring for now the effects of any topological constraints on interactions, the dynamics of such models are typically captured by the general stochastic differential equation $\dot{z}=f\left(z\right)+\eta_{z}\left(t\right)$. The form of the function f is determined by the actions of the voters and it, in turn, determines large‐scale outcomes for the population (i.e., whether or not consensus can form and its duration), while a Gaussian noise term $\eta_{z}$ captures the (system‐state‐dependent) intrinsic stochasticity arising from the random timing of events in a finite population.

In such systems, two qualitatively different consensus‐forming behaviors are well‐understood and have been widely observed in the literature [36, 37, 38], as illustrated in Figure 1a,b. First, if the average response of an individual voter to a change in the overall population is linear (i.e., voters consider at most one other individual when updating their opinion) then f(z) is at most quadratic and consensus can only form when the system is driven by noise to a boundary system state (z=±1) of total agreement [36]. As the effect of noise scales inversely with group size, only small groups can reach consensus in a reasonable time frame in this case. Second, permitting more complicated non‐linear responses (for example two X voters may cooperate to recruit a Y voter) allows for cubic f(z) and the possibility of stable interior fixed points (positions in which majority of individuals but not all agree on an opinion and for which the proportion of individuals holding that opinion is stable in time) that can be rapidly selected [37, 39]. As we will show, this apparent dichotomy between noise‐driven consensus and complex behavior models is in fact resolved when neutral agents are incorporated into the population.

**FIGURE 1:**
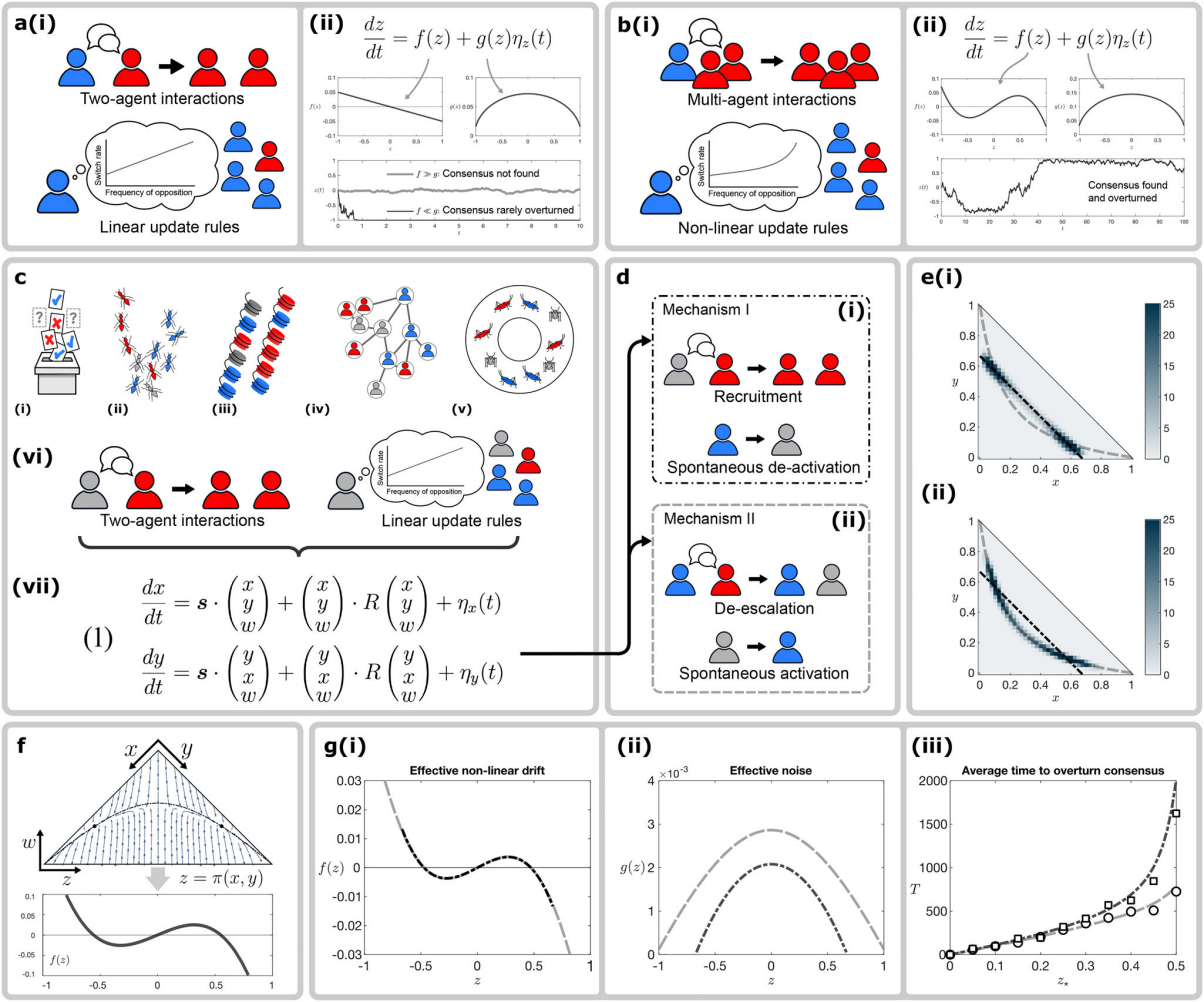
Neutrality resolves the apparent trade‐off between simplicity and flexibility in consensus models, enabling both robust formation and rapid switching of consensus without resorting to complex multi‐body interactions. (a) Simple low‐order symmetric voter models (e.g., Biancalani et al. [36]) do not explain the emergence and overturning of consensus. (i) The rate of switching opinions is a linear function of the density of opposition. Equivalently, at most two‐body interaction events are considered. (ii) Representative stochastic dynamics exhibit linear (or zero) drift and system‐state‐dependent diffusion; resulting time series either stay near an undecided (z=0) system state, or spend long periods at a boundary system state (z=1,−1). (b) Complex, higher‐order models (e.g., Dyson et al. [37]) can exhibit consensus formation and switching. (i) Less realistic multi‐body interactions or non‐linear rates of opinion switching are necessary. (ii) Representative stochastic dynamics exhibit cubic (or higher‐order) drift and system‐state‐dependent diffusion; resulting time series can exhibit stochastic transitions between a symmetric pair of interior consensus system states. (c) Our model describes a broad class of symmetric binary decision models with neutral agents that can be used to represent a wide range of processes e.g., voting with abstention  [50, 51] (ii) insect foraging [57, 58] (iii) nucleosome conformal modification [5, 59] (iv) social network dynamics [60] (v) onset of collective motion [37]. (vi) We consider at most two‐body interactions or, equivalently, linear rates of switching. (vii) General dynamics on the 2‐D simplex with rates s for spontaneous stance changes and R for reactive changes (derivation in Methods Section 7.1). (d) The dominant reaction dynamics for the two main mechanisms of consensus formation ‐ based on either (i) recruitment (for Mechanism I) or (ii) de‐escalation (for Mechanism II) interactions. (e) Dynamics associated to each mechanism. (i) Linear manifold (black dashed line). (ii) Hyperbolic manifold (grey dashed line). In each case the heat map shows the simulated residency duration at each point in system state space; the stochastic dynamics remain close to the predicted slow manifolds. Note that both manifolds are shown on each plot for ease of comparison. (f) A timescale separation allows us to project from the simplex to effective non‐linear dynamics of z (details in Methods Section 7.3). (g) The amplified effect of noise in Mechanism II allows for faster overturning of consensus. (i) Effective non‐linear drift. Parameters are chosen so that the effective drifts align. (ii) Effective noise is much larger for Mechanism II than Mechanism I leading to (iii) time to overturn consensus as a function of polarization, $z_{★}$ (the deterministic fixed points of the drift), at consensus being higher for Mechanism I than Mechanism II.

Of equal importance to consensus formation, but far less well studied, is the question of how consensus is overturned. In many contexts, it is vital that group decisions do not remain fixed in the face of new evidence; echo‐chamber effects must be suppressed so that the expressed collective preferences are not too strongly entrenched. Switching between consensus system states^1^ has been observed in alignment‐mediated models of animal collective motion [4, 40] (including the locust experiments analyzed herein^2^), bacterial cell migration  [42], eukaryotic cell specialization  [43], and neural decision making  [44] amongst other applications  [45, 46].

In some consensus formation situations levels of participation can vary, with some individuals temporarily adopting a neutral position [7, 8]. A number of different models that consider undecided individuals exist in the literature  [47, 48]. The models of Balenzuela et al. [49] show that undecided agents can strongly influence whether groups reach consensus or remain polarized. It has also previously been hypothesized that agents with intermediate ‘neutral’ stances may play a role in the dynamics of consensus change in models of politics  [50, 51], language  [52, 53], swarm robotics  [54], and general decision‐making  [55]. These models consider the possibility that individuals may hold a neutral stance when transitioning between opinions X and Y. Here, we will explore if the same is true of the population as a whole, asking if the transit from one system state consensus to another is characterized – or even facilitated – by an increase in neutrality.

Our theoretical analysis of a general model of binary collective decision problems with neutral agents uncovers two primary mechanisms of consensus formation and change. One is dominated by recruitment‐type interactions in which opinionated agents seek to convert others to their view (much in line with the political practice of canvassing). The other is characterized by a de‐escalation dynamic in which disagreement between opinionated agents results in one adopting a neutral stance and later updating their opinion spontaneously.

We find that the second mechanism provides a vastly more efficient route to the overturning of consensus; the population traverses a pathway through a high‐neutrality system state with decreased effective population size, creating a high‐noise environment that quickly allows the re‐establishment of a new consensus. We validate this finding with a new analysis of experimental data of the onset of collective motion in locusts  [4], and independently reproduce it in new experiments of a voting game with human participants. We argue that the ability to rapidly form and change consensus is the reason that neutral agents are favored in these real consensus‐forming systems, offering crucial insights into the ways in which group‐level decision‐making can be supported.

## 1 Neutral Agents Provide a Parsimonious Explanation of Consensus Formation

We introduce a general class of symmetric, autonomous collective decision dynamics with neutrality. Consider a population of fixed size N, composed of individuals that may choose either one of two decision options, X or Y, or can hold a neutral position, W. The frequencies of the types are denoted x, y, and w, respectively, and, as above, we write z=x−y for the strength of consensus. Individuals may change their stance spontaneously or by interacting with any other member of the population.

We make the parsimonious assumption that transition rates depend at most linearly on the current state of the system. In event‐based realizations of the model, this corresponds to transitions occurring either spontaneously (e.g., X→W), or responsively through pairwise interactions such as recruitment (e.g., X+W→X+X) or repulsion (e.g., X+W→X+Y). The lack of self‐interactions and the presence of X—Y symmetry means the general model is characterized by nine positive parameters; the full set of available actions/interactions are illustrated in Table SI.1.

From general principles (see Methods Section 7.1), we derive the governing stochastic dynamics

(1) dxdt=s·xlylwl+xlylwl·Rxlylwl+ηx(t),dydt=s·ylxlwl+ylxlwl·Rylxlwl+ηy(t),

where s is a three‐vector of parameters capturing the effect of spontaneous changes (i.e., individuals changing stance uninfluenced by others), R is a 3×3 matrix (with zero diagonal ‐ excluding self‐interaction) parameterising responsive events (i.e., individuals changing stance as a result of interacting with another individual through pairwise interactions). The terms $\eta_{x}$ and $\eta_{y}$ are corresponding multiplicative Gaussian noise; see Methods Section 7.1 for a complete characterization. Conservation of mass closes the system and confines the dynamics to a 2‐D simplex (see e.g. Figure 1e, f), in which the deterministic part is a plane quadratic system whose phase portrait [56] describes the emergence of consensus. Stable consensus system states correspond to attractive symmetric fixed points (positioned at the intersection of the curved and straight manifolds in Figure 1e) dominated by one of X or Y. These consensus system states may lie on the interior or boundary of the simplex, depending on parameters; a full classification is provided in Section SI.1.

## 2 Two Mechanisms to Overturn Consensus

Depending on the relative dynamical prominence of the spontaneous and responsive events, two qualitatively different consensus transition mechanisms are determined. Mechanism I: individuals tend to adopt a neutral position spontaneously, from which they may then be recruited by interaction with a non‐neutral agent (see Figure 1d(i) for the dominant reaction dynamics). Intuitively, each time an individual becomes neutral spontaneously, another individual quickly takes an opinionated stance, meaning that the number of neutral agents stays roughly constant. Mechanism II: individuals become neutral through interaction with another individual, later returning to an opinionated stance spontaneously (see Figure 1d(ii) for the dominant reaction dynamics). In this case, as the system transitions away from a consensus system state, individuals are converted to neutrality faster than they spontaneously revert to an opinionated stance, leading to an overall increase in neutral agents for lower levels of agreement. The reverse process takes place as the system transitions back toward consensus. For both of these mechanisms, in appropriate parameter regimes, the trajectories of the stochastic system are strongly attracted to a 1‐D slow manifold lying on the heteroclinic orbit between stable consensus system states (see Figure 1e). The shape of this path is determialloned by the role of neutrality in the system. For Mechanism I, the manifold describes a straight line with a constant proportion, α, of neutral agents (see Figure 1e(i)); for Mechanism II it follows a hyperbola with curvature controlled by γ passing through a higher‐neutrality system state (see Figure 1e(ii)). The coefficients α and γ are defined in terms of the system parameters in Section 7.2 of the Methods, and in the Supporting Information (see Sections SI.1.1– SI.1.3) we demonstrate that these are the only physically realisable low‐dimensional dynamics possible.

Projection of the fast outer flow field [61, 62, 63, 64] yields effective dynamical equations for consensus strength, z, for each mechanism. To leading order they are

(2) $$\begin{array}{cc} \mathrm{Mechanism}I:\; & \dot{z}=\beta z\frac{\left[p(z)-4\alpha \right]}{2(1-\alpha )}+\sqrt{\frac{2\rho \alpha }{N\gamma (1-\alpha )}p\left(z\right)}\;\eta_{z}\left(t\right),\\ \mathrm{Mechanism}\mathrm{II}:\; & \dot{z}=\rho z\left[w\left(z\right)-\alpha \right]+\sqrt{\frac{4\beta }{N}w\left(z\right)}\;\eta_{z}\left(t\right), \end{array}$$

where $p\left(z\right)=\gamma [{\left(1-\alpha \right)}^{2}-z^{2}]$ and $w\left(z\right)=\left(2+\gamma -\sqrt{4+4\gamma +\gamma^{2}z^{2}}\right)/\gamma$ gives w=1−x−y as a function of z=x−y along the hyperbola 1−x−y−γxy=0. The composite parameters β and ρ control the relative weight of the two mechanisms. Full details of the derivation of this result are given in Section 7.3 of the Methods.

## 3 Transitions Through States of High System Neutrality Enable Faster Consensus Switching

Equations (2) characterize our primary theoretical result and have several interesting features which we now explore. Where the original formulation (1) was quadratic, the slow manifold projection of the higher‐dimensional system generates higher‐order non‐linearity in the reduced dynamics, allowing stable interior consensus system states (see Figure 1e,f) that would require non‐linear individual interaction mechanisms in models without neutral agents (see Figure 1b) [37]. This supports the conclusion that in our model with neutrality no more than two‐body interactions are required to enable the formation of non‐unanimous consensus system states ‐ it alleviates the previous requirement that two agents must work together to change the opinion of a third  [37]. Note that for both mechanisms, the fixed points of the deterministic system (found by solving p(z)=4α or w(z)=α) both lie on the intersection of the linear and hyperbolic manifolds (see Figure 1e), allowing for direct comparison of the two dynamical regimes. Since the effective dynamics are univariate, we are able to straightforwardly compute the mean transit time (see Figure 1g(iii)) between opposing consensus system states (see Methods Section 7.3.3) for both mechanisms. We find that manifolds exhibiting intermediate curvature (Mechanism II), whereby the route between consensus system states passes first into a system state with increased neutrality (but avoiding an all‐neutral system state of collective indecision), offer transition times that are orders of magnitude faster than what can be achieved with linear manifolds imposing a fixed proportion of neutral agents (Mechanism I). The pathway through state‐space that traverses higher levels of neutral agents may be favored in real consensus‐forming systems because the reduced effective population size creates a high‐noise environment that quickly allows the re‐establishment of a new consensus.

These results together imply that neutrality is an integral component of both the formation of stable consensus, and the allowing of efficient transit to alternative consensus system states. To assess the practical relevance of our new theory, we have tested its premises and conclusions in two model systems of real‐world consensus formation.

## 4 Marching Locusts Exhibit Consensus Switching Through States of High System Neutrality

**FIGURE 2:**
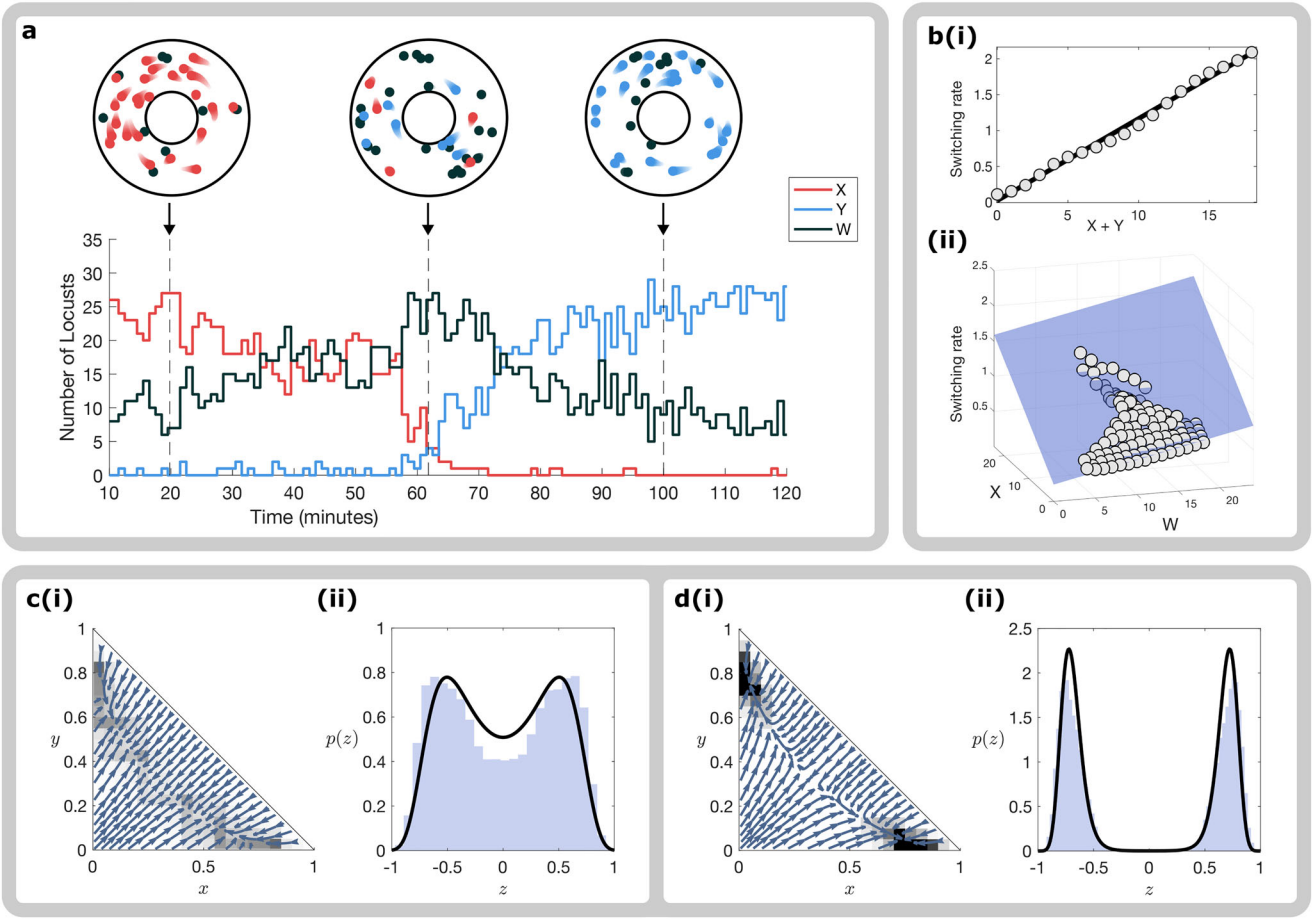
Locust consensus switches pass through high‐neutrality states, confirming that 'de‐escalation dynamics’ predicted by our model operate in real biological systems. (a) An experimental time‐course and snapshots (N=35 locusts) showing a consensus switch between predominantly clockwise (red) and anti‐clockwise (blue) motion, with an increase in neutral (stopped ‐ black) individuals during the transient. (b) Linear rate functions fit the data (white circles) well. (i) The rate at which individual stopped locusts resume motion as a function of the number of moving locusts is well modelled as a straight line. (ii) The rate at which individual locusts moving in one orientation change this orientation as a function of the number of stopped locusts and the number of locusts moving in the opposite direction is well‐fitted by a plane. (c) Model fitted to data (see text) for experiments with N=25 locusts. (i) 2‐D state space residency distribution (greyscale heat map) with overlaid model flow fields (blue arrows). (ii) The corresponding 1‐D projection of the stationary distribution from the experiment (blue histogram) onto the slow manifold and the model predicted stationary probability distribution (black curve) for experiments. (d) As for (c) but with N=70 locusts.

When placed in a ring‐shaped arena at sufficiently high density, locust nymphs have been found to demonstrate consensus formation in their choice of travel direction around the arena  [4]. Identifying clockwise and anticlockwise motion as the X and Y options of a binary choice problem, the emergent collective motion is typically characterized by periods of consensus punctuated by occasional rapid transitions (see Figure 2a), which are less common in larger group sizes. Previous studies comparing experimental data with simple two‐type mathematical models (without neutrality) have established evidence for consensus switching to be driven by demographic noise, but with a requirement of non‐linear (three‐body or higher) reaction terms  [65]. Direct examination of locust trajectories in the experimental data reveals that individuals frequently adopt a third ‘neutral’ option ‐ stopping (see Methods Section 7.5 for precise definitions). Some previous modelling studies have heuristically incorporated a stopped stance [66, 67, 68], but have not identified the profound consequences for the collective which we now explain.

We initially construct a spatial agent‐based simulation model of locust motion, which (for parameter values derived from experimental data ‐ see Sections SI.2.3 and SI.4) can be mapped to the effective model (2) above. A population of N agents is simulated in an annular domain (see Figure 2a), either stopped or moving deterministically with fixed angular velocity (see Figure 2a for an experimental time‐course exhibiting a switch in consensus). Changes of stance may occur spontaneously, or through interaction with a nearby agent ‐ resulting in a linear dependence of transition rates on the frequencies of types in the population, on average. This modelling framework generalizes the model frameworks of Biancalani et al. [36] and Dyson et al. [37]. Their models arise as special cases of our framework under particular parameter choices.

Rotational symmetry of the model implies the existence of spatially homogenous dynamical system states, but there is no a priori reason to assume these to be stable or representative of the experimental data. In Section SI.2, we prove the stability of rotationally symmetric system states to spatial perturbations and give a detailed mathematical derivation of equation (2) from the spatial simulation model. In Section SI.4, we also present statistical evidence for the lack of spatial correlation in the global dynamics of the population, reinforcing that our well‐mixed model is a suitable representation of the locust behavior.

Moreover, parameters of the effective low‐dimensional dynamics of model (2) can be determined from experimental data (see Methods Section 7.5), providing support for the linear interaction (i.e., at most two‐body interactions) model (see Figure 2b) and predicting a curved manifold indicative of Mechanism II dynamics (see Figure 2c(i),d(i)). We note that, consistent with our proposed Mechanism II, the curvature of the manifold is such that the high‐neutrality system state has a reduced active population size which is consequently more susceptible to demographic noise. The stationary distribution of model (2) can be directly computed and shows excellent agreement with the experimentally observed behavior (see Figure 2c(ii), d(ii)), further validating the theory underlying Mechanism II, which predicts a curved manifold joining consensus system states.

## 5 Consensus Switching Through Increased Neutrality is Observed in a Human Voting Game

**FIGURE 3:**
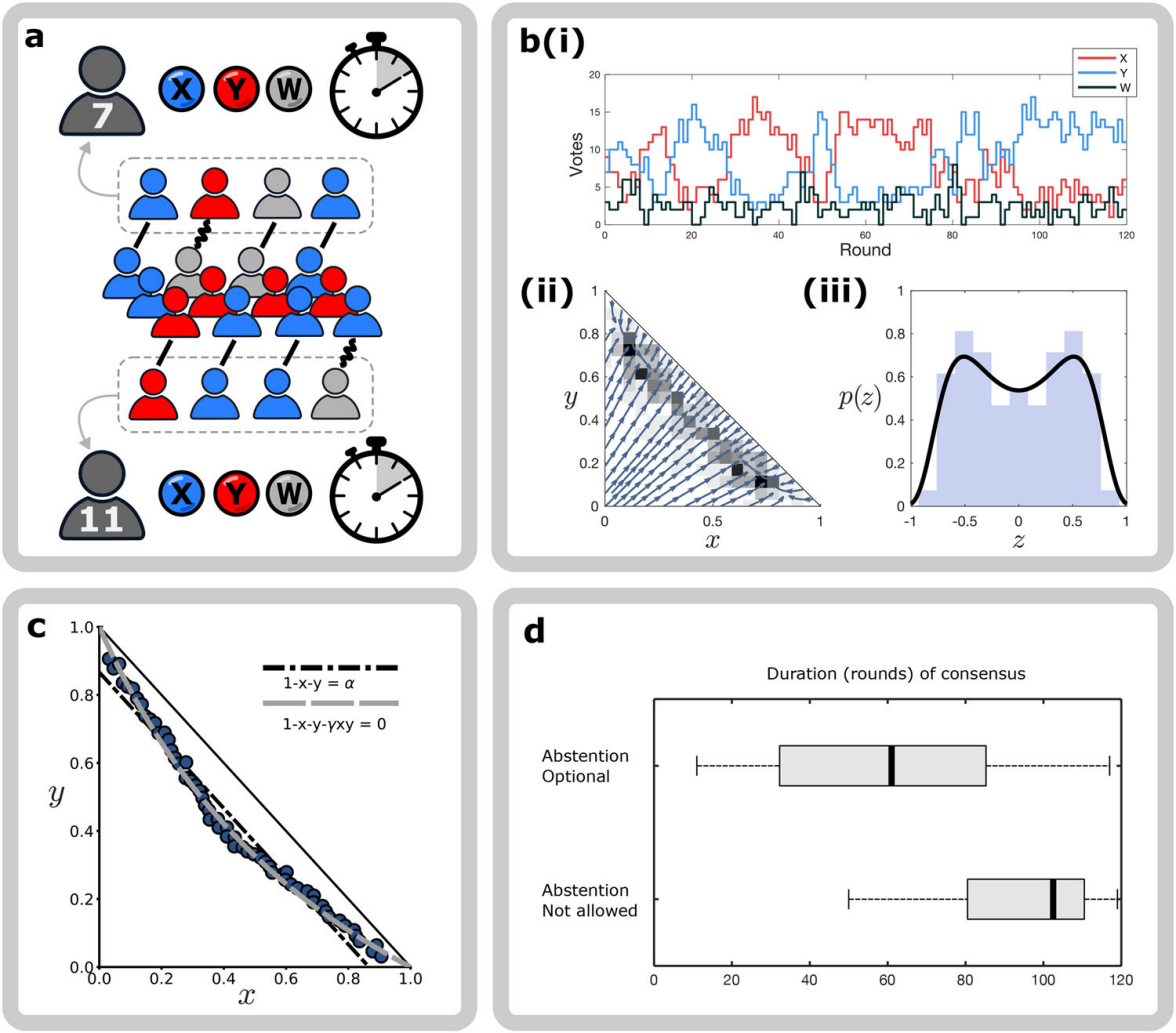
Human groups switch consensus through states of high abstention. Removing neutrality dramatically reduces consensus duration ‐ confirming neutral agents' critical role in adaptive decision‐making. (a) Schematic representing one round of our human voting experiments with 12 participants. Two examples of samples of four votes (avatars inside the dashed line) of participants from the previous round (the true votes are indicated by the 12 colored avatars in the middle) shown to individuals numbered 7 and 11 (dark grey with numbers). Most of the uniformly sampled participants' votes are displayed faithfully as indicated by straight black lines (e.g., a blue character mapped by a straight black line will always appear as a blue character in the sample) while some are stochastically flipped as indicated by wiggly black lines (e.g., a grey character mapped by a wiggly black line flips to a red character in the sample shown to participant 7). Individuals are then allowed to vote for option X, option Y or to abstain (by choosing option W or not making a choice) within each round's ten‐second voting period. (b) An example experiment with N=19 participants (see Section SI.6.3 for examples with different numbers of participants). (i) Time‐courses of the experimental data. Votes for option X, option Y and abstentions are represented in blue, red and black, respectively. (ii) A heat map of the data from the experimental time‐course in (i) with the flowfield of the corresponding deterministic model trajectories (with parameters inferred from the experimental data ‐ see Section SI.6.2–SI.6.3) superimposed. (iii) A 1D projection of the stationary probability distributions over the single variable z=x−y (blue bars ‐ see Sections SI.1 and SI.6 for details). The theoretical stationary probability distribution from the model with experimentally inferred parameters (see Section SI.6.2–SI.6.3) is superimposed as a black curve. (c) The curved manifold is a superior fit to the data than the straight manifold (see Methods Section 7.8). (d) Duration of periods of observed consensus (in number of rounds of voting) in experiments with abstaining optional (upper) and with no abstention option given (lower). For each replicate in both conditions the duration of consensus is measured as the sum of the periods between passing from z>0.75 to z<−0.75 (or vice versa) or until the end of the experiment at round 120.

The general relevance of our theory and its findings was further explored through new experiments on human consensus‐seeking behavior in an anonymous iterated voting game (with variable numbers of participants per group). Participants were given the choice to vote for one of two options at a small cost, with a reward (of value one and a half times that of the voting cost) given to all those voting with the majority; an additional ‘abstain’ option was also offered, with no direct associated cost or reward. After each round participants were shown a noisy subset of responses from the previous round (sampled uniformly at random with some responses we switched ‐ see Methods Section 7.6 for specifics) before voting in the next round (see Figure 3a).

In the Section SI.6, we show how a discrete‐time simulation model of the voting experiment again reduces to the effective dynamics (1). Again our model can be parameterized using the voting experimental time‐course data (see Sections SI.6.2– SI.6.3). The stationary probability distributions computed using our theory and the fitted parameters show good agreement with those of the experiment (see Figure 3b(iii)). Crucially, we again find that the system exhibits consensus switching (see Figure 3b(i)) via Mechanism II curved manifold dynamics (see Figure 3c); this is reflected in the heat map (see Figure 3b(ii)) which demonstrates that the highest probability path between consensus positions is through higher‐neutrality system states. Statistical analyses establish with high confidence that these paths are indeed curved (see Methods Section 7.8 and Figure 3c).

In further experiments, weremoved the option to abstain (see Section SI.6.4); in these experiments we found either a failure to settle on a robust consensus, or a substantially slower transit between consensus system states (see Figure 3d), consistent with the previously presented theory illustrated in Figure 1, demonstrating the crucial importance of neutral actors for consensus formation and change.

## 6 Discussion

We have postulated a refined model which allows the formation of consensus amongst groups of individuals holding two opinion types and a third neutral position. Our model is stripped of extraneous detail in order to have the most general applicability. We note that incorporating additional features can bring other effects to the fore. When symmetry is broken, for example by a minority group strongly pushing one option, the population can be rapidly driven to a chosen consensus system state, although this effect is weakened by the presence of uninformed individuals  [69]. When interactions are constrained by topology — for example if voters are arranged in a network  [70] or have spatially localized interactions  [71] — then domains of consensus (groups of proximate individuals sharing the same opinion) grow and coarsen  [38], with the fate of the system being determined by the behavior of the domain interfaces. Nevertheless, the qualitative categorization in terms of an effective stochastic differential equation is found to hold in many cases.

In addition to those systems that we have studied in detail here, we have identified other applications (ranging from nucleosome conformal modification [5, 59] to genetic switching  [72]) for which our theory offers explanatory insights. Specifically, in the nucleosome modification model of Dodd et al. [5] the roles of the active stances are played by acetylated/methylated nucleosomes, while neutral stances corresponds to unmodified nucleosomes. The model allows for consensus formation and change, exhibiting switching via either the curved or the straight manifold, dependent on parameter choices (see Section SI.7). The plethora of model application areas suggests the broad applicability of our general theory across dynamical systems.

The theoretical insights generated by our model suggest that rapid switching between consensus system states can be facilitated by neutral agents who serve to lower the effective population size. This has a significant consequence for the underpinning behaviors needed to allow adaptive decision‐making in groups. Systems in which individuals' preferences are reinforced by in‐group dynamics are found to spend orders of magnitude more time stuck in one consensus system state than those in which encounters with opposing opinions prompt the adoption of a more neutral stance.

Our theoretical and experimental analyses point to a new understanding of both formation and change of consensus across a broad range of systems, two of which ‐ in locust marching and human voting games ‐ we have explicitly investigated through experiments. Consequently, we can suggest strategies for overturning entrenched consensus. In modern elections, much political energy is spent attempting to win over the so‐called floating voter  [73]. We posit that efforts might be better expended on moderating the positions of those holding strong opinions, so that they temporarily adopt a neutral stance. Neutral individuals should then be allowed to independently make up their own minds about which position they choose to adopt. This is our Mechanism II ‘de‐escalation’ strategy, which appears to be naturally favored in systems such as those we have examined here, and should allow for the more efficient overturning of consensus.

## 7 Methods

### 7.1 Derivation of Stochastic Dynamics Main Text Equation (1)

All events involve a single agent changing stance, with rate modelled as a linear function of the current densities of the other agents. For example, the event X→Y has total rate $\rho_{XY}=n_{X}\left(a_{XY}+b_{XY}n_{Y}/N+c_{XY}n_{W}/N\right)$. The terms here are explained as follows: the prefactor $n_{X}$ counts the number of X agents that might change stance; $a_{XY}$ is the rate parameter for spontaneous change from X to Y; $b_{XY}$ is the rate of recruitment of X agents by Y agents, with $n_{Y}/N$ giving the density of Y agents; $c_{XY}$ is the rate of repulsion of W agents by X agents so that they convert to Y agents, and $n_{W}/N$ is the corresponding density of W agents. The stoichiometry vector for the change of an X into a Y is $\nu_{XY}=\left(-1,1,0\right)$. Each of the five other possible change events have rates and stoichiometric vectors described similarly. Note that we impose X—Y symmetry on the rate parameters so that, for example, $a_{XY}=a_{YX}$.

Introducing scaled variables $\left(x,y,w\right)=\left(n_{X},n_{Y},n_{W}\right)/N$, the theorem of Kurtz [74], states that for large N the dynamics converge to solutions of the stochastic differential equation

(3) ddtxlylwl=∑jνjϱj+1N∑jνjϱjζj(t),

where the sums run over all possible events, and the terms $\zeta_{j}\left(t\right)$ denote independent standard Gaussian white noise variables. With the rates and stoichiometry vectors described above we obtain the forms given in equation (1),

dxdt=s·xlylwl+xlylwl·Rxlylwl+ηx(t),dydt=s·ylxlwl+ylxlwl·Rylxlwl+ηy(t),

where

(4) $$s=\left(\begin{array}{c} -a_{XY}-a_{XW}\\ a_{XY}\\ a_{WX} \end{array}\right)\;R=\left(\begin{array}{ccc} 0 & -c_{XW} & -c_{XY}-b_{XW}\\ 0 & 0 & c_{XY}\\ b_{WX} & c_{WX} & 0 \end{array}\right).$$

The noise terms $\eta_{x}\left(t\right)$ and $\eta_{y}\left(t\right)$ are related to the noise terms in Equation (3) according to

(5) $$\begin{array}{cc} \eta_{x} & =\frac{1}{\sqrt{N}}\left(-\sqrt{\rho_{XY}}\zeta_{XY}-\sqrt{\rho_{XW}}\zeta_{XW}+\sqrt{\rho_{YX}}\zeta_{YX}+\sqrt{\rho_{WX}}\zeta_{WX}\right),\\ \eta_{y} & =\frac{1}{\sqrt{N}}\left(\sqrt{\rho_{XY}}\zeta_{XY}-\sqrt{\rho_{YX}}\zeta_{YX}-\sqrt{\rho_{YW}}\zeta_{YW}+\sqrt{\rho_{WY}}\zeta_{WY}\right),\; \end{array}$$

In the supplement, we provide an alternative derivation of this result, modelling the events as ‘chemical reactions’ that are either spontaneous (e.g., X→W) or a result of two‐body interactions (e.g., X+Y→X+W). The resulting expressions are identical.

### 7.2 Geometry of the drift

The deterministic (or ‘drift') part of the system (1) can be decomposed into functions defining two straight lines and one hyperbola. Specifically, introducing the composite parameters

(6) $$\begin{array}{cc} & \alpha =\frac{a_{XY}+a_{XW}}{b_{WX}-b_{XW}-c_{XY}}\;,\;\gamma =\frac{c_{XW}}{a_{WX}}\;,\\ & \beta =a_{WX}\;,\;\rho =b_{WX}-b_{XW}-c_{XY}\;,\\ & \delta =c_{XY}+c_{WX}\;,\;\chi =\frac{a_{XY}}{c_{XY}+c_{WX}}\;, \end{array}$$

we obtain

(7) $$\begin{array}{cc} \frac{dx}{dt}=\; & \beta (1-x-y-\gamma xy)+\rho x(1-x-y-\alpha )\\ & +\delta y\left(1-x-y+\chi \right)+\eta_{x}\left(t\right),\\ \frac{dy}{dt}=\; & \beta (1-x-y-\gamma xy)+\rho y(1-x-y-\alpha )\\ & +\delta x\left(1-x-y+\chi \right)+\eta_{y}\left(t\right). \end{array}$$

The zero sets of the bracketed terms common to both equations in system (7) have different geometries: 1−x−y−γxy=0 is a hyperbola with curvature determined by γ; 1−x−y−α=0 is a straight line with a fixed positive proportion α of neutral agents; 1−x−y+χ=0 is a straight line parallel to the first but lying outside of the simplex, as χ is positive. These features will be key to the remaining analysis.

In many real‐world scenarios (in particular the applications we are concerned with here), the rate parameter $c_{XY}$ is likely to be negligibly small, as it relates to the event in which an encounter with a neutral agent results in a reversal of stance of an active agent. Similarly, $c_{WX}$ describes the rate with which neutral agents take up an opposing position after an encounter with an active agent, which again we consider to be negligible for most salient purposes. We are therefore motivated to make the simplifying assumption δ=0, whereby system (7) reduces to

(8) $$\frac{dx}{dt}=f\left(x,y\right)+\eta_{x}\left(t\right)\;,\;\frac{dy}{dt}=f\left(y,x\right)+\eta_{y}\left(t\right)\;,$$

where

(9) f(x,y)=β(1−x−y−γxy)+ρx(1−x−y−α),

Stable fixed points f(x,y)=f(y,x)=0 are found at the intersection of the line 1−x−y−α=0 and the hyperbola 1−x−y−γxy=0, with an unstable fixed point between them on the line x=y. The behavior of the stochastic system is characterized by periods spent in the vicinity of one or other stable fixed point, punctuated by traversal along the heteroclinic orbit between them. Depending on which of β and ρ dominates, this heteroclinic orbit is close to either the line or the hyperbola.

When β≫ρ we have the ‘fast’ outer system $\dot{x}\approx \dot{y}\approx \beta \left(1-x-y-\gamma xy\right)$. In this case trajectories starting at a general point in the simplex will collapse quickly along lines of constant z (because $\dot{z}=\dot{x}-\dot{y}\approx 0$) to the hyperbolic manifold 1−x−y−γxy=0. Conversely, when ρ≫β, the outer system has $\dot{x}/\dot{y}\approx x/y$, with solutions y∝x, collapsing rapidly to the linear manifold 1−x−y−α=0. This separation into fast and slow timescales allows us to derive an effective stochastic equation for the dynamics of z.

### 7.3 Slow Manifold Projection

To extract a 1‐D description of the dynamics of z(t) from system (8), we apply the method of Katzenberger [61], as expounded on in Parsons and Rogers  [64]. To enable a streamlined presentation of the theory we will make the simplifying assumption that direct transitions between active stances are rare, i.e., $a_{XY}\ll 1$ and $b_{XY}\ll 1$. This assumption is not strictly necessary for the analysis (full calculations are given in Sections SI.1.4– SI.1.5), however it will simplify the resulting expressions, as $\eta_{x}\left(t\right)$ and $\eta_{y}\left(t\right)$ become independent with variance $\langle \eta_{x}\left(t\right)\eta_{x}\left(t\right)\rangle =g\left(x,y\right)$ and $\langle \eta_{y}\left(t\right)\eta_{y}\left(t\right)\rangle =g\left(y,x\right)$, where

(10) $$g\left(x,y\right)=\frac{1}{N}\left((1-x-y)(\beta +\rho x)+xy\beta \gamma +x\rho \omega \right),$$

For both mechanisms we proceed by identifying a function that maps from the simplex to the manifold along the trajectory lines of the fast outer system. From there we derive a stochastic description for z alone, treated as a coordinate of the manifold.

#### 7.3.1 Mechanism I: Straight Manifold

As explained above, the action of the fast outer flow field for this mechanism is projection onto the line 1−x−y−α=0, while keeping fixed the ratio of x to y. This is captured mathematically by the function $\pi_{I}\left(x,y\right)=\left(1-\alpha \right)\left(x-y\right)/\left(x+y\right)$, which maps from a general point (x,y) in the simplex to the resulting value of z after projection to the straight line manifold. Writing $z=\pi_{I}\left(x,y\right)$, application of Itō's lemma yields a stochastic differential equation for z:

(11) $$\begin{array}{c} \frac{dz}{dt}=\frac{\partial \pi_{I}}{\partial x}\frac{dx}{dt}+\frac{\partial \pi_{I}}{\partial y}\frac{dy}{dt}+\frac{1}{2}\frac{\partial^{2}\pi_{I}}{\partial x^{2}}\left\langle \eta_{x}\left(t\right)\eta_{x}\left(t\right)\right\rangle +\frac{1}{2}\frac{\partial^{2}\pi_{I}}{\partial y^{2}}\left\langle \eta_{y}\left(t\right)\eta_{y}\left(t\right)\right\rangle . \end{array}$$

Following Katzenberger [61, 64], we close this expression by approximating x and y by their values on the manifold for a given z. In this case x≈(1−α+z)/2 and y≈(1−α−z)/2. The result is an expression for z alone:

(12) $$\begin{array}{c} \frac{dz}{dt}=\beta \frac{z(p(z)-4\alpha )}{2(1-\alpha )}-\frac{\beta }{N}\frac{z(p(z)-4\alpha )}{2{\left(1-\alpha \right)}^{2}}+\sqrt{\frac{2\rho \alpha }{N\gamma (1-\alpha )}p\left(z\right)}\;\eta_{z}\left(t\right), \end{array}$$

where $p\left(z\right)=\gamma [{\left(1-\alpha \right)}^{2}-z^{2}]$ and $\eta_{z}\left(t\right)$ is a standard Gaussian white noise. It is important to note that while the large parameter ρ controls the fast collapse to the manifold, it is the small parameter β that governs the slow dynamics on the manifold. With N large and β small, we may neglect the sub‐dominant β/N term to obtain the expression from system  (2) in the main text. For moderate values of N, this term has an interesting effect on the dynamics which is fully explored in the Supporting Information (see Section SI.1.4).

#### 7.3.2 Mechanism II: Hyperbolic Manifold

In the case of the hyperbolic manifold, the projection operator has the more simple form $\pi_{\text{II}}\left(x,y\right)=x-y$. For a given z the corresponding coordinates (x,y) on the manifold can be written x=(w(z)+z)/2, y=(w(z)−z)/2, where we have introduced $w\left(z\right)=\left(2+\gamma -\sqrt{4+4\gamma +\gamma^{2}z^{2}}\right)/\gamma$. Performing the same analysis as above yields the other result from system (2) for Mechanism II:

(13) $$\frac{dz}{dt}=\rho z\left[w\left(z\right)-\alpha \right]+\sqrt{\frac{4\beta }{N}w\left(z\right)}\;\eta_{z}\left(t\right),$$

where $\eta_{z}\left(t\right)$ is again a standard Gaussian white noise. Note that the linearity of $\pi_{\text{II}}$ leads to the absence of any N‐dependent terms in the deterministic part for this mechanism.

#### 7.3.3 Stationary Distributions and Mean Transit Times

The advantage of a 1‐D description of the effective stochastic dynamics is that the statistical properties can be computed exactly [75]. For a univariate stochastic differential equation of the general form

(14) $$\frac{dz}{dt}=f\left(z\right)+\sqrt{g(z)}\eta_{z}\left(t\right),$$

the stationary distribution is found as

(15) $$p_{★}\left(z\right)=\frac{C}{g(z)}\exp\int_{z_{\min}}^{z}\frac{2f(z^{\prime })}{g(z^{\prime })}dz^{\prime },$$

where C is the appropriate normalizing constant and $z_{\min}$ is the lower boundary for z (which will be either 1−α or −1 for our straight line or hyperbolic manifolds, respectively). The mean transit time between locations $z_{0}$ and $z_{1}$ is given by

(16) $$\tau \left(z_{0}\to z_{1}\right)=2\int_{z_{0}}^{z_{1}}\frac{1}{g\left(z\right)p_{★}\left(z\right)}\int_{z_{\min}}^{z}p_{★}\left(z^{\prime }\right)dz^{\prime }dz\;.$$

For both of our mechanisms, exact formulas can be obtained for $p_{★}$ and τ, although they are complicated and not immediately enlightening; we present them in the Supporting Information(see Sections SI.1.4 and SI.1.5).

### 7.4 Agent‐Based Model for Locust Motion in an Annular Arena

We simulate a population of N agents moving in a 1‐D annular domain of length L. At any moment each agent is either moving clockwise with constant velocity v (we denote this stance X), anticlockwise with velocity −v (stance Y) or is stationary (neutral stance W). Following the so‐called ‘lambda‐rho’ approach for spatial chemical reaction models  [76], we define an interaction radius R and specify that two agents separated by less than this distance may interact, resulting in a change of stance with a given probability. Informed by analysis of tracking data (see below) we specify the events and rates:

X⇄r2r1WX+W→r32WX+W→r42XY⇄r2r1WY+W→r32WY+W→r42YX+Y→r5X+WX+Y→r5W+YX+Y→r62W.

Writing $\varphi_{T}\left(\theta \right)$ for the density of agents of type T with angular position θ, for large N, one may derive the stochastic intergo‐partial‐differential equation system

(17a) $$\begin{array}{cc} \partial_{t}\varphi_{X} & =-v\partial_{\theta }\varphi_{X}+r_{2}\varphi_{W}-r_{1}\varphi_{X}+\varphi_{W}\int_{|\theta -\theta^{\prime }|<R}r_{4}\varphi_{X}\left(\theta^{\prime }\right)d\theta^{\prime }\\ & \;-\varphi_{X}\left(\int_{|\theta -\theta^{\prime }|<R}r_{3}\varphi_{W}\left(\theta^{\prime }\right)d\theta^{\prime }+\int_{|\theta -\theta^{\prime }|<R}r_{5}\varphi_{Y}\left(\theta^{\prime }\right)d\theta^{\prime }\right)+\eta_{X}\left(\theta ,t\right), \end{array}$$

(17b) $$\begin{array}{cc} \partial_{t}\varphi_{Y} & =v\partial_{\theta }\varphi_{Y}+r_{2}\varphi_{W}-r_{1}\varphi_{Y}+\varphi_{W}\int_{|\theta -\theta^{\prime }|<R}r_{4}\varphi_{Y}\left(\theta^{\prime }\right)d\theta^{\prime }\\ & \;-\varphi_{Y}\left(\int_{|\theta -\theta^{\prime }|<R}r_{3}\varphi_{W}\left(\theta^{\prime }\right)d\theta^{\prime }+\int_{|\theta -\theta^{\prime }|<R}r_{5}\varphi_{X}\left(\theta^{\prime }\right)d\theta^{\prime }\right)+\eta_{Y}\left(\theta ,t\right), \end{array}$$

(17c) $$\begin{array}{cc} \partial_{t}\varphi_{W} & =r_{1}\left(\varphi_{X}+\varphi_{Y}\right)-2r_{2}\varphi_{W}-\varphi_{W}\left(\int_{|\theta -\theta^{\prime }|<R}r_{4}\left(\varphi_{X}+\varphi_{Y}\right)\left(\theta^{\prime }\right)d\theta^{\prime }\right)\\ & \;+\varphi_{X}\left(\int_{|\theta -\theta^{\prime }|<R}r_{5}\varphi_{Y}\left(\theta^{\prime }\right)d\theta^{\prime }\right)+\varphi_{Y}\left(\int_{|\theta -\theta^{\prime }|<R}r_{5}\varphi_{X}\left(\theta^{\prime }\right)d\theta^{\prime }\right)\\ & \;+\left(\varphi_{X}+\varphi_{Y}\right)\left(\int_{|\theta -\theta^{\prime }|<R}r_{3}\varphi_{W}\left(\theta^{\prime }\right)d\theta^{\prime }\right)+\eta_{W}\left(\theta ,t\right). \end{array}$$

Full details of the process to obtain this result are given in the Supporting Information (see Section SI.2.2). The terms $\eta_{X}$, $\eta_{Y}$, and $\eta_{W}$ are spatiotemporal Gaussian noises with a correlation structure similar to those of the noise terms in system (1). Although the spatial structure of system (17) appears complex, one can show that the family of spatially homogeneous solutions of the form $\varphi_{X}\left(\theta \right)\equiv x\left(t\right)/2\pi ,\;\;\varphi_{Y}\left(\theta \right)\equiv y\left(t\right)/2\pi$ are stable in the deterministic limit N→∞ (full details of this calculation are given in the Supporting Information (see Section SI.2.3)). We conclude that spatial effects are not the dominant factor in transitions between consensus system states.st

In the Section SI.2.4, we show how the SDE system (1) may be derived from this model when either of the interaction radius or the agent velocity are sufficiently large. The parameters $\left(a_{XY},\;b_{XY},\;\text{…}\right)$ are obtained from $\left(r_{1},r_{2},\text{…}\right)$ after appropriate rescaling by 2R/L and v/L.

We establish the range of applicability of the map between the spatial ahypnd well‐mixed models with general parameters by comparing stationary probability distributions in the spatial and well‐mixed models (see Section SI.4.3 for more details).

### 7.5 Parameter Fitting for Locust Experiments

Summary: We analyzed video‐tracking data from multiple locust marching experiments originally reported by Buhl et al. [4]. Each experiment involved groups of between 5 and 100 locust nymphs in an annular arena, recorded at five frames per second. At each time point individuals were classified as clockwise, anticlockwise, or stopped (neutral) using a moving‐average velocity threshold (0.005 rad/frame averaged over ten frames). We aggregated data across all available replicates and verified that the observed dynamics were consistent across group sizes and replicates (see Section SI.4.1). Parameter inference for the stochastic model was performed using an equation‐free method applied to pooled data, and fitted models reproduced the stationary distributions and switching behavior observed in each replicate (Figure 2c,d). Additional checks confirmed that spatial correlations were negligible, supporting the well‐mixed approximation used in the analysis (see Figure SI.6).

We test our theoretical findings against empirical data from experiments on the spontaneous collective motion of locust nymphs placed in an annular arena. Details of the experimental protocols are provided in Buhl et al.  [4]

We work with video tracking data giving the coordinates of locusts in the experimental arena measured with a frequency of five frames per second. For each frame we classify each locust as either ‘stopped’ (i.e., neutral, W) or moving clockwise (X) or anticlockwise (Y), based on a moving average velocity over the previous ten frames with a threshold of 0.005 rad/frame to be classified as moving or stopped.

We use a 2‐D variant of the equation‐free method  [37, 65]. The locusts orientation data can be aggregated into a natural integer discretization over the simplex. For each coordinate pair $\left(x_{i},y_{i}\right)$, we collect all the data time‐points visiting that point and look at the distributions of stances after a short period δt. The mean and covariance of this distribution then provide point estimates for the drift and diffusion at $\left(x_{i},y_{i}\right)$. In practice, we found the best results using δt=0.2s, corresponding to advancing a single frame.

From the equation‐free estimates for drift and diffusion, we then extract parameters using the functional forms derived above (see Equations (9) and (10)) for our theoretical model. Write $\tilde{f}\left(x_{i},y_{i}\right)$ and $\tilde{g}\left(x_{i},y_{i}\right)$ for the estimates of the drift and diffusion terms for the point $\left(x_{i},y_{i}\right)$ in the data set obtained using the equation‐free method. These must be compared to the values $f(x_{i},y_{i}|r)$ and $g(x_{i},y_{i}|r)$ obtained from Equations (9) and (10), where we have made the dependence on the parameter r explicit. To fit parameters, we define the weighted error function of a parameter set r to be

(18) $$\begin{array}{ccc} \text{Er}(r) & = & \sum_{i}\left[\omega_{i}{\left(\tilde{f}\left(x_{i},y_{i}\right)-f\left(x_{i},y_{i}|r\right)\right)}^{2}\right.\\ & & \left.+\sqrt{\omega_{i}}{\left(\tilde{g}\left(x_{i},y_{i}\right)-g\left(x_{i},y_{i}|r\right)\right)}^{2}\right], \end{array}$$

where the sum runs over all bins i in our discretization of the simplex, and $\omega_{i}$ counts the number of data points in that bin. Note that typically not all possible pairs $\left(x_{i},y_{i}\right)$ are present in the data, hence, the sum is only over the observed pairs. Since we are fitting a symmetric model, we symmetrize the data by duplicating it with x and y values swapped. We then minimize Er(r) using MATLAB's particleswarm function with tolerance 1e‐9 to perform the particle swarm minimization algorithm  [77, 78, 79].

### 7.6 Iterated Human Voting Experimental Protocol

We here summarize the protocol and analysis of the experiments performed on an online iterated voting/abstention game. Anonymous participants were recruited to play a voting game in which repeatedly choosing the option that was most popular with the rest of the group would yield a financial reward at the end of the experiment. The game was programmed in oTree  [80], and deployed on the online platform Prolific  [81].

Participants were recruited via Prolific over an hour‐long window before each experimental session started^3^. The numbers of participants in each experimental replicate was varied in order to determine whether there was an impact of group size on voting behavior. To be included in the study, participants had to be English‐speaking residents of the UK between the ages of 18 and 75 (the average age was 37). People who had played the game previously were prevented from joining again. After completing informed consent, participants were invited to join another virtual room with all other participants in their experimental group.

After reading the instructions and undertaking five practice rounds, participants played 120 rounds of the voting game. In each round of the game participants had 10 seconds to choose between Option X, Option Y or an Abstain option. Failing to make a decision in time resulted in automatic abstention. Voting would cost participants 100 points, while abstaining would not cost any points. Participants who voted with the majority in that round would receive a reward of 150 points (in the case of a tie no points were awarded).

To inform their choice, in each round each participant was presented with a noisy random sample of nine responses from the previous round. Noise is then introduced in the sample by allowing the potential for each of the nine drawn responses to be modified with probability 0.4. If a drawn response is selected to be ‘modified’ in the sample, it is substituted by a uniformly drawn decision between Option X, Option Y or Abstain. Note that this sampling strategy means that, one third of the time, responses selected for ‘modification’ will be reassigned their original response. We note that modifications occur only in the sample shown to participants and not in the recorded votes. The first sample shown to all participants contained only Y votes.

**FIGURE 4:**
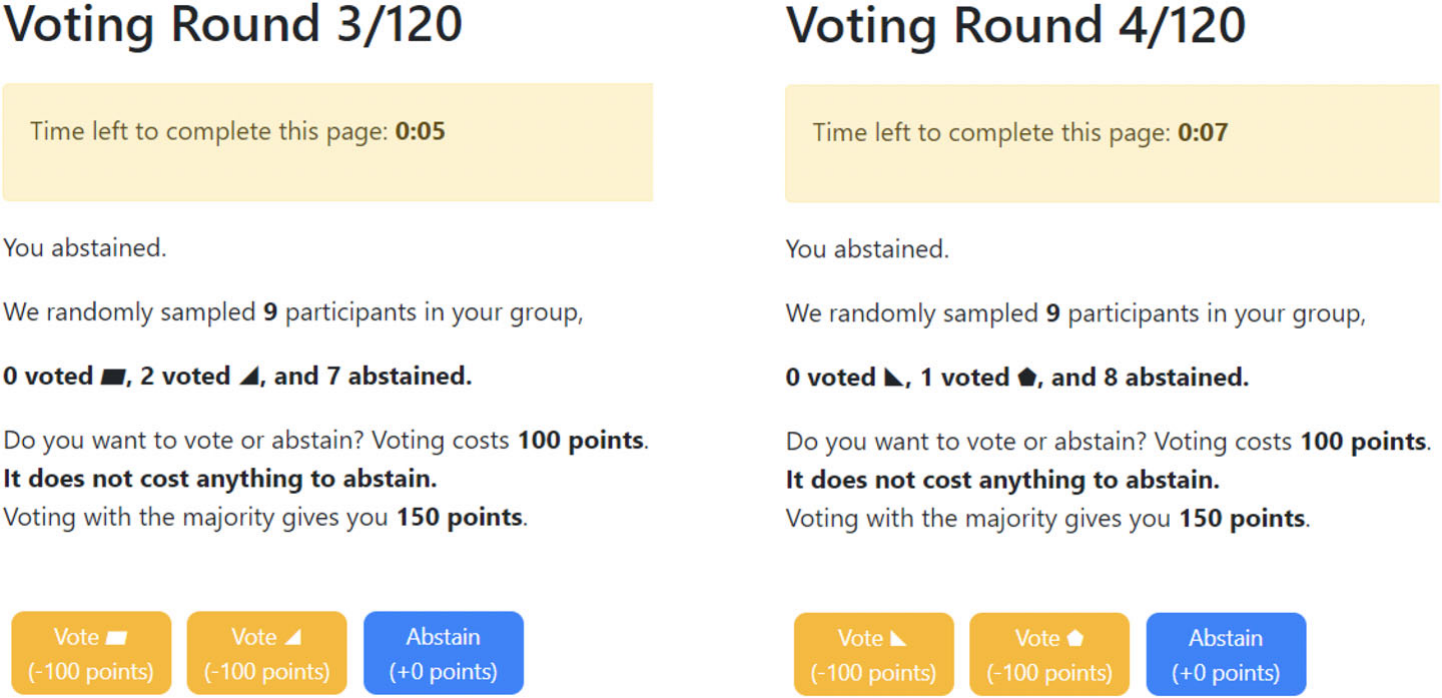
Examples of the screen a participant might see in a round of the iterated voting game. The options were labelled with different shapes (square, parallelogram, left right‐triangle, right right‐triangle, rhombus, pentagon, and hexagon). In every round, we randomly assigned a different shape‐label to options X and Y to reduce the effect of preferences and we randomized the position of the buttons between X and Y to reduce memory effects. The color of the vote and abstain buttons was determined at random for each participant but kept the same throughout the experiment.

The formatting of the screens presented to participants was partially randomized to reduce memory and preference effects. Figure 4 demonstrates a possible example of a screen seen by a participant in two consecutive rounds.

Participants who failed to click a vote or abstain button for five consecutive rounds were presented with an activity check screen, giving them 10 s to click a button to confirm they are actively playing the game. Failing activity checks twice resulted in a ‘dropout’. Dropouts were substituted by bots to keep the flow of the game. Bots always voted for the option with a lead of two or more in the shown sample and abstained if the difference in votes for option X and option Y was one or less. No more than one dropout occurred in any of the replicates. Given their low frequency, these substitutions did not materially influence the experimental outcomes.

At the end of the session, participants saw their ranking. A fixed fee for participation (5.25 GBP) was paid, along with a bonus payment (5.25 GBP) to the top 50% of participants ranked in each group.

### 7.7 Calibration and Control Experiments

We conducted two calibration experiments to obtain the empirical distribution of the probability of voting for each option X,Y or choosing to abstain and to help tune the randomization protocol. Between calibration experiments, we varied the number of previous votes in the sample shown to participants (5 vs. 9).

The calibration experiments followed the same procedure as the main experiments, except that the “information” presented to participants was fixed and not drawn from the actual responses. Across the rounds we presented samples covering all possible combinations of voting and abstention responses. Each combination was repeated as many times as possible in the 120 rounds of the experiment in a random order. There are 12 unique (accounting for X—Y symmetry) combinations of the three stances a sample of five votes (so each combination was repeated ten times over 120 rounds). Similarly there are 30 unique (accounting for X—Y symmetry) combinations for a sample of nine votes (so each combination was repeated four times over 120 rounds).

Seven participants completed the experiment with a sample of five votes, providing 70 data points for each combination, and a further 11 participants completed the experiment with a sample of nine votes, providing 44 data points for each combination. This data was used to estimate the voting distribution between stances X, Y and Abstain.

Using the above two empirical distributions, we simulated the experiment to determine parameters which would increase the probability of observing a switch in consensus within the 120 rounds of the experiments. In the simulation, we varied group sizes and noise in the sample votes for sample sizes of 5 and 9 votes. The highest likelihood of observing a switch was obtained with the randomization protocol described above, with 15 to 20 participants, and an information sample size of nine. These values were employed in the experiments described in the previous section.

We conducted eight control experiments without the option to abstain in groups with an average of 16 participants. In those experiments which exhibited consensus formation, consensus was maintained for longer when abstention was prohibited (see Figure 3d of the main text). See Section SI.5 for protocols and Section SI.6.4 for experimental time‐courses.

### 7.8 Voting Experiment Manifold Curvature Analysis

We ran a total of 12 replicates of the main protocol, with group sizes 12, 13, 16, 18, 19, 19, 20, 22, 25, 28, 31, and 33. To assess which of our theoretical manifolds better explains the results from the voting experiments with abstentions, we performed least squares fitting of the data to the general curve 1−x−y−γxy=α, with the straight line and the hyperbola being the special cases γ=0 and α=0, respectively. We attempted the fitting using both of the projection operators $\pi_{I}$ and $\pi_{\text{II}}$ identified in the theoretical analysis above (see Section 7.3). The resulting parameter fits and residuals $\sigma^{2}$ are summarized in Table 1.

**TABLE 1:** Parameter fits (± Standard Errors), residuals and Bayesian Information Criterion for the voting experiment data under two possible projections to three different manifolds.

	$\pi_{I}$	$\pi_{\text{II}}$
Line	α=0.144±0.005	α=0.137±0.006
	$\sigma^{2}=441.59$, BIC =314.54.	$\sigma^{2}=800.42$, BIC =385.10
Hyperbola	γ=1.021±0.025,	γ=1.062±0.027
	$\sigma^{2}=136.34$, BIC =254.60	$\sigma^{2}=167.69$, BIC =296.00
Combined	γ=0.89±0.10, α=0.020±0.014,	γ=0.92±0.09, α=0.020±0.012,
	$\sigma^{2}=131.34$, BIC =256.63	$\sigma^{2}=159.58$, BIC =297.22.

To test the hypothesis that either γ or α is zero in the combined fit, we perform a z‐test on the parameters of the combined fit. The z‐test gives a score suggesting how many standard deviations the estimated value is from the theoretical hypothesis. The larger the score, the less likely the error is to explain the difference between the hypothetical and the observed values. This is not to be confused with the alignment z=X−Y defined earlier in the manuscript. For the projection $\pi_{I}$, we find $z_{\gamma }=8.9$ (p‐value <0.001, right‐tailed test), while for the $\pi_{\text{II}}$ projection, $z_{\gamma }=10.2$ (p‐value <0.001, right‐tailed test). In both cases, this suggests that the data present strong evidence for curvature of the slow manifold.

For the coefficient, α, which characterizes the linear manifold, $z_{\alpha }=1.43$ (p‐value =0.076, right‐tailed test) under the $\pi_{I}$ projection and $z_{\alpha }=1.67$ (p‐value =0.048, right‐tailed test) under the $\pi_{\text{II}}$ projection. We therefore do not reject the null hypothesis that α is zero under the first projection at 0.05 significance level.

Table 1 also shows that the hyperbola fit has a much lower sum of squared residuals than the linear fit (suggesting the hyperbola is a better fit). We employ the Bayesian Information Criterion (BIC)  [82] for model selection, finding support for the hyperbolic fit. The BIC penalizes models with extra parameters that do not better explain the data. By computing the BIC for different proposed models, we can compare them against each other – the lower the BIC, the better the model is at describing the data. For the hyperbolic fit, the BICs are 254.60 and 296.00 for projections $\pi_{I}$ and $\pi_{\text{II}}$, respectively; while for the combined fit, we obtain 256.63 and 297.22, as the extra degree of freedom in the parametrization does not significantly improve the reduced squared error when compared to the hyperbolic fit. For the linear fit, the BICs are 314.54 and 385.10, much higher than the models with curvature. Therefore, the hyperbolic model should be selected.

Hence, from both model selection and hypothesis testing on the fitting of both parameters, we can conclude that the hyperbolic manifold provides a better fit to participants’ voting behavior in the voting game with the option to abstain. This implies that as the groups switch from one consensus system state to another, the system transitions through a state of increased abstentions (neutrality).

### 7.9 Ethics

The Social Science Research Ethics Committee at the University of Bath approved the study (0164–499 and 4996–5145), and the experiment was performed in line with institutional review board guidelines. Participants gave informed consent before the study.

## Author Contributions

A.S., C.Y., and T.R. conceived the study and performed the theoretical analysis. A.S. performed the new analysis of data – provided by Buhl – from  Buhl et al. [4] A.S., C.Y., and T.R. conceived of the voting study; A.S. and J.H. developed the experimental design. A.S. gathered the data and performed analysis of the voting study with support from J.H. C.Y. and T.R. drafted the main text of the manuscript, A.S. drafted the supplement. All authors contributed to the final writing of the manuscript.

## Conflicts of Interest

The authors declare no conflicts of interest.

## Supporting information


**Supporting File**: advs74005‐sup‐0001‐SuppMat.pdf.

## Data Availability

All raw and processed data generated during this study, along with the code used for analysis, have been deposited in University of Bath. These resources are publicly accessible and can be retrieved using the following DOI: https://doi.org/10.15125/BATH‐01478 [83].

## Endnotes

1 For clarity we will refer to the overall state of the system as the ‘system state’, while the status of an individual will be referred to as its ‘stance’.

2 It is worth noting that recent work on locust swarms has demonstrated that collective motion can emerge without explicit alignment, instead relying on internal consensus dynamics and sensory integration [41].

3 In total across all experimental replicates we recruited 256 participants (75 men, 181 women)
