# Supplementary material for: Consensus Formation and Change are Enhanced by Neutrality
Source: Adv Sci (Weinh). 2026 Mar 22:e12301. Online ahead of print. doi: 10.1002/advs.202512301 (PMC13325515; doi:10.1002/advs.202512301)
Supplement: Supplementary file 1 — Supporting File: advs74005‐sup‐0001‐SuppMat.pdf. [file ADVS-9999-e12301-s001.pdf]

Consensus formation and change are enhanced by neutrality
Supplementary Information

Andrei Sontag[1,2,*], Janina Hoffmann[3], Tim Rogers[1,*], Chirstian A. Yates[1,*]

[1]Department of Mathematical Sciences, University of Bath, Bath, UK

[2]Department of Mathematics, University College London, UK

[3]Department of Psychology, University of Bath, Bath, UK

*Email: a.sontag@ucl.ac.uk, t.c.rogers@bath.ac.uk, c.yates@bath.ac.uk

Supplementary Information

SI.1 General linear response model and submanifolds

In both the locust and human voting experiments, the system comprises a fixed number of particles that can interact and change stance as a result of these interactions. The particles can adopt either one of two active stances – moving left or right (for locusts), voting for option X or Y (for voters) – or a neutral stance W – stopping (for locusts) or abstaining (for voters). It is then natural to model both systems under the same general modelling framework, in which the rates of stance change are described in terms of the current state of the system. The simplest assumption for the responses is that the rate of change of stance varies at most linearly with the state of the system.

Our modelling approach considers the rate of change of state $\mathcal{S} \equiv (n_X, n_Y, n_W)$ of the system. n_X, n_Y and n_W are the numbers of particles with stances X, Y and W, respectively. Similar approaches have been used in the context of the collective behaviour of ants [Biancalani et al., 2014] and locusts [Dyson et al., 2015]. Note that $n_X + n_Y + n_W = N$ is a constant of the system, the total group size. Considering all possible interactions between any pair of two distinct-stance particles that change only one species, corresponding to a linear response of each particle to the system's state, we obtain a microscopic description of the system as detailed in Table SI.1, with the corresponding reaction rates, ϱ, and stoichiometry vectors, $\boldsymbol{\nu}$.

A mesoscopic description of the system in terms of stochastic differential equations (SDEs) is more amenable to analysis. It is obtained straightforwardly as

$$\frac{\mathrm{d}\boldsymbol{x}(t)}{\mathrm{d}t} = \sum_{j=1}^{q} \boldsymbol{\nu}_j \varrho_j(\boldsymbol{x}(t)) + \sum_{j=1}^{q} \boldsymbol{\nu}_j \sqrt{\varrho_j(\boldsymbol{x}(t))}\zeta_j(t), \tag{SI.1.1}$$

for $\boldsymbol{x}(t) = \left(\frac{n_X(t)}{N}, \frac{n_Y(t)}{N}\right)^T = (x, y)^T$. The equation for $w = \frac{n_W}{N}$ is obtained from $\frac{\mathrm{d}w}{\mathrm{d}t} = -\frac{\mathrm{d}x}{\mathrm{d}t} - \frac{\mathrm{d}y}{\mathrm{d}t}$ due to conservation of mass. Here, q is the number of reaction channels, $\boldsymbol{\nu}_j$ is the stoichiometric vector of reaction j, and $\varrho_j(\boldsymbol{x})$ is the corresponding reaction rate. $\zeta_j(t)$ are independent white noise with correlations $\langle \zeta_i(t) \rangle = 0$ and $\langle \zeta_i(t)\zeta_j(t') \rangle = \delta(t - t')\delta(i - j)$, for $\langle \cdot \rangle$ the expected value. Combining independent ζ_j where possible, we obtain, for our system,

$$\frac{d\boldsymbol{x}}{dt} = \boldsymbol{F}(\boldsymbol{x}) \ + \sqrt{N^{-1}}\boldsymbol{G}(\boldsymbol{x})\boldsymbol{\zeta}(t), \tag{SI.1.2}$$

Reaction	Reaction rate (ϱ)	Stoichiometry vector ($\boldsymbol{\nu}$)
$X \underset{a_{XY}}{\overset{a_{XY}}{\rightleftarrows}} Y$	$a_{XY}n_X$<br>$a_{XY}n_Y$	$(-1, 1, 0)$<br>$(1, -1, 0)$
$X \underset{a_{WX}}{\overset{a_{XW}}{\rightleftarrows}} W$	$a_{XW}n_X$<br>$a_{WX}n_W$	$(-1, 0, 1)$<br>$(1, 0, -1)$
$Y \underset{a_{WX}}{\overset{a_{XW}}{\rightleftarrows}} W$	$a_{XW}n_Y$<br>$a_{WX}n_W$	$(0, -1, 1)$<br>$(0, 1, -1)$
$X + Y \xrightarrow{b_{XY}} 2X$<br>$X + Y \xrightarrow{b_{XY}} 2Y$	$b_{XY}\frac{n_X n_Y}{N}$	$(1, -1, 0)$<br>$(-1, 1, 0)$
$X + W \xrightarrow{b_{WX}} 2X$<br>$X + W \xrightarrow{b_{XW}} 2W$	$b_{WX}\frac{n_X n_W}{N}$<br>$b_{XW}\frac{n_X n_W}{N}$	$(1, 0, -1)$<br>$(-1, 0, 1)$
$Y + W \xrightarrow{b_{WX}} 2Y$<br>$Y + W \xrightarrow{b_{XW}} 2W$	$b_{WX}\frac{n_Y n_W}{N}$<br>$b_{XW}\frac{n_Y n_W}{N}$	$(0, 1, -1)$<br>$(0, -1, 1)$
$X + Y \xrightarrow{c_{XW}} X + W$<br>$X + Y \xrightarrow{c_{XW}} W + Y$	$c_{XW}\frac{n_X n_Y}{N}$	$(0, -1, 1)$<br>$(-1, 0, 1)$
$X + W \xrightarrow{c_{XY}} Y + W$<br>$Y + W \xrightarrow{c_{XY}} X + W$	$c_{XY}\frac{n_X n_W}{N}$<br>$c_{XY}\frac{n_Y n_W}{N}$	$(-1, 1, 0)$<br>$(1, -1, 0)$
$X + W \xrightarrow{c_{WX}} X + Y$<br>$Y + W \xrightarrow{c_{WX}} Y + X$	$c_{WX}\frac{n_X n_W}{N}$<br>$c_{WX}\frac{n_Y n_W}{N}$	$(0, 1, -1)$<br>$(1, 0, -1)$

Table SI.1: Complete set of reactions in our microscopic model, assuming at most two-body interactions, X and Y symmetry and the absence of self-interactions. The middle column gives the aggregate rate of the reactions in the population. The right column gives the stoichiometry vectors – the changes in population numbers after the reaction has happened. Imposing X and Y symmetry, the number of distinct parameters reduces from 18 down to 9: $a_{XY} = a_{YX}$, $a_{XW} = a_{YW}$, $a_{WX} = a_{WY}$, $b_{XY} = b_{YX}$, $b_{XW} = b_{WY}$, $b_{WX} = b_{WY}$, $c_{XY} = c_{YX}$, $c_{XW} = c_{YW}$, $c_{WX} = c_{WY}$.

where the drift, $\boldsymbol{F(x)}$, is

$$\boldsymbol{F(x)} = \begin{pmatrix} -(a_{XY} + a_{XW})x - c_{XW}xy + (b_{WX} - b_{XW} - c_{XY})xw \\ \qquad +a_{XY}y + a_{WX}w + (c_{XY} + c_{WX})yw \\ \\ a_{XY}x - c_{XW}xy + (c_{XY} + c_{WX})xw - (a_{XY} + a_{XW})y \\ \qquad +a_{WX}w + (b_{WX} - b_{XW} - c_{XY})yw \end{pmatrix},$$

$\boldsymbol{\zeta} = (\zeta_1, \zeta_2, \zeta_3)^T$, and noise matrix $\boldsymbol{G} = \begin{pmatrix} \boldsymbol{G_1} \\ \boldsymbol{G_2} \end{pmatrix}_{2\times 3}$, with rows

$$\boldsymbol{G_1} = \begin{pmatrix} \sqrt{a_{XW}x + b_{XW}xw + c_{XW}xy + a_{WX}w + b_{WX}xw + c_{WX}yw} \\ 0 \\ \sqrt{a_{XY}x + 2b_{XY}xy + c_{XY}xw + a_{XY}y + c_{XY}yw} \end{pmatrix}^T, \quad \boldsymbol{G_2} = \begin{pmatrix} 0 \\ \sqrt{a_{XW}y + b_{XW}yw + c_{XW}xy + a_{WX}w + b_{WX}yw + c_{WX}xw} \\ -\sqrt{a_{XY}x + 2b_{XY}xy + c_{XY}xw + a_{XY}y + c_{XY}yw} \end{pmatrix}^T, \tag{SI.1.3}$$

where $w = 1 - x - y$. Compactly, we can rewrite our system in the form,

$$\frac{dx}{dt} = \boldsymbol{s} \cdot \begin{pmatrix} x \\ y \\ w \end{pmatrix} + \begin{pmatrix} x \\ y \\ w \end{pmatrix} \cdot R \begin{pmatrix} x \\ y \\ w \end{pmatrix} + \eta_x(t), \quad \frac{dy}{dt} = \boldsymbol{s} \cdot \begin{pmatrix} y \\ x \\ w \end{pmatrix} + \begin{pmatrix} y \\ x \\ w \end{pmatrix} \cdot R \begin{pmatrix} y \\ x \\ w \end{pmatrix} + \eta_y(t),$$

corresponding to equation (1) of the manuscript with

$$\boldsymbol{s} = \begin{pmatrix} -a_{XY} - a_{XW} \\ a_{XY} \\ a_{WX} \end{pmatrix} \qquad R = \begin{pmatrix} 0 & -c_{XW} & -c_{XY} - b_{XW} \\ 0 & 0 & c_{XY} \\ b_{WX} & c_{WX} & 0 \end{pmatrix}. \tag{SI.1.4}$$

The noise terms are given by,

$$\eta_x(t) = \sqrt{N^{-1}} \left[G_{11}\zeta_1(t) + G_{13}\zeta_3(t) \right], \tag{SI.1.5}$$

$$\eta_y(t) = \sqrt{N^{-1}} \left[G_{22}\zeta_2(t) - G_{13}\zeta_3(t) \right]. \tag{SI.1.6}$$

It will be convenient to explore the symmetries of the system and change variables to $z = (X - Y)/N$ and $u = x + y = 1 - w$. Applying Itô's Lemma, it follows that $dz_t = dx_t - dy_t$ and $du_t = dx_t + dy_t$, for dx_t and dy_t given by Eq. (SI.1.2). Hence,

$$\begin{aligned} \frac{dz}{dt} =& - (a_{XW} + 2a_{XY})z + (b_{WX} - b_{XW} - 2c_{XY} - c_{WX})z(1-u) \\ &+ \frac{1}{N}\{G_{11}\eta_1 - G_{22}\eta_2 + 2G_{13}\eta_3\}, \\ \frac{du}{dt} =& - a_{XW}u + 2a_{WX}(1-u) - \frac{c_{XW}}{2}(u^2 - z^2) + (b_{WX} - b_{WX} + c_{WX})u(1-u) \\ &+ \frac{1}{N}\{G_{11}\eta_1 + G_{22}\eta_2\}. \end{aligned} \tag{SI.1.7}$$

Before going into the stochastic dynamics, we will focus on characterising the possible behaviours of the system in the deterministic limit of $N \to \infty$. The fixed points of the system are defined by the intersections of the nullclines defined by Eqs. (SI.1.7). There is always a

fixed point at $z = 0$ inside the simplex, and possibly two more may be found, given by

$$z_{\pm} = \sqrt{\Delta}, \qquad \Delta = (1-\alpha)^2 - \frac{4[a_{WX}\alpha + (c_{XY} + c_{WX})\alpha(1-\alpha)]}{c_{XW}}, \tag{SI.1.8}$$

where $\alpha = \frac{2a_{XY}+a_{XW}}{b_{WX}-b_{XW}-c_{WX}-2c_{XY}}$. The conditions for the existence of these points inside the simplex are $0 \leq \alpha \leq 1$ and $0 \leq \Delta \leq 1$, in which case the polarised fixed points $z_{\pm}$ are always stable. Otherwise, the fixed point with $z = 0$ is stable.

The behaviours of the model can be summarised in two categories based on the intersection of the nullclines defined by Eqs. (SI.1.7): (i) the nullclines intersect three times inside the simplex at $z = 0$ and $z_{\pm}$. $z_{\pm}$ are stable. (ii) the nullclines intersect only once at $z = 0$, or thrice, but $z_{\pm}$ is outside the simplex. $z = 0$ is stable. In Fig. SI.1 we show the model's possible behaviours.

To determine the zones of mono and bistability, we computed the fixed points of the model. We can assume the simplifying assumption that a_{XY}, c_{XY}, and c_{WX} are orders of magnitude smaller than the remaining parameters. The reason is that a_{XY} is a spontaneous transition from $X \to Y$, which has been observed to be much rarer in both the locust and voting experiments than $X/Y \leftrightarrow W$ transitions. Similarly, c_{XY} is a transition in which a voter changes to an opposite opinion after interacting with a neutral agent, while c_{WX} corresponds to a neutral agent taking opinion X after interacting with another with opinion Y. Both of these are expected (and observed) to be much rarer than the remaining interactions. In this case, we can focus on the two parameters α, defined previously, and $\kappa = \frac{a_{WX}}{c_{XW}}$, such that the fixed points are

$$z^*_{\pm} = \sqrt{(1-\alpha)^2 - 4\alpha\kappa}, \tag{SI.1.9}$$

setting $a_{XY} = c_{XY} = c_{WX} = 0$ in Eq. (SI.1.8). Fig. SI.2 shows the regions of mono and bistability and the location of our voting game experiments in the parameter space (see SI Section SI.5 and SI.6 for details), showing that the voting game experiments are mostly located near a boundary region between mono and bistability. Fig. SI.1 also shows that the deterministic trajectories of the system follow the flow field, converging quickly to a lower dimensional manifold before moving towards the fixed points. Similarly, in the finite-size model, the system fluctuates around the slow manifold as it transitions between consensus system states (see Fig. SI.3). This suggests a separation of timescales we explore in the following sections to reduce the system's dimensionality and obtain analytical approximations for the stationary probability distribution and mean transition times between consensus system states.

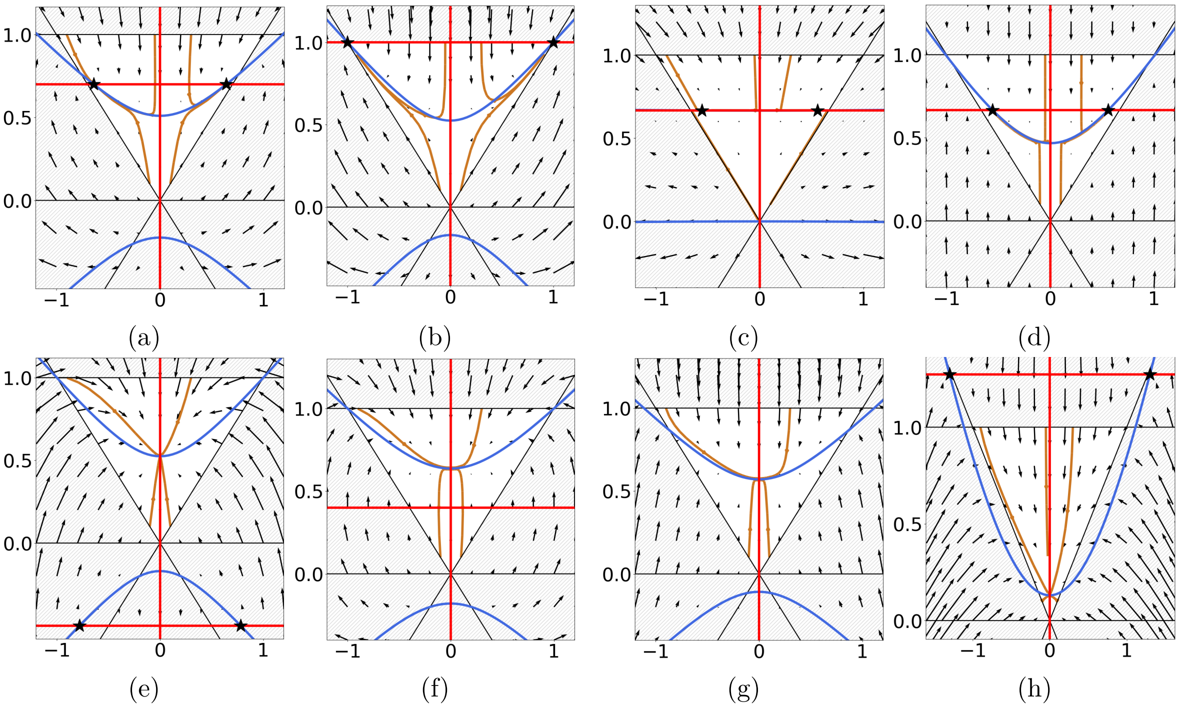

Figure SI.1: Our general model captures a range of qualitatively distinct deterministic behaviours of consensus dynamics, revealing how parameter choices determine whether groups exhibit bistability with polarized consensus or converge to neutrality. The arrows indicate the direction and strength of the flow field at certain points. The orange curves are trajectories of the system in the phase plane (z, u) for different initial conditions. (a-d) The nullclines $dz/dt = 0$ (i.e., $z = 0$, $1 - u = \alpha$, in red) and $du/dt = 0$ (in blue) intersect three times inside the simplex (unshaded area). The two polarised fixed points are always stable (stars). (a) Parameters: $a_{XY} = 0, b_{XY} = 0, c_{XY} = 0, a_{XW} = 0.6, b_{XW} = 3, c_{XW} = 3, a_{WX} = 0.2, b_{WX} = 5, c_{WX} = 0$. (b) Fixed points at the boundary. Parameters: $a_{XY} = 0, b_{XY} = 1, c_{XY} = 0, a_{XW} = 0, b_{XW} = 3, c_{XW} = 5, a_{WX} = 0.2, b_{WX} = 5, c_{WX} = 0$. (c) The trajectories converge to the line $1 - u = \alpha$. Parameters: $a_{XY} = 0, b_{XY} = 0, c_{XY} = 0, a_{XW} = 10, b_{XW} = 1, c_{XW} = 0.1, a_{WX} = 0.01, b_{WX} = 31, c_{WX} = 0$. (d) The trajectories converge to the nullcline $du/dt = 0$. Parameters: $a_{XY} = 0, b_{XY} = 0, c_{XY} = 0, a_{XW} = 0.1, b_{XW} = 0.01, c_{XW} = 10, a_{WX} = 1, b_{WX} = 0.31, c_{WX} = 0$. (e-f) The nullclines do not intersect more than once inside the simplex (f) or intersect outside the simplex (e, h). The fixed point with $z = 0$ is stable. (e) Nullclines intersect at $z < 0$. Parameters: $a_{XY} = 1.5, b_{XY} = 1, c_{XY} = 0, a_{XW} = 0, b_{XW} = 3, c_{XW} = 5, a_{WX} = 0.2, b_{WX} = 5, c_{WX} = 0$. (f) The nullcline $1 - u = \alpha$ does not intersect the hyperbolas. Parameters: $a_{XY} = 0.6, b_{XY} = 0, c_{XY} = 0, a_{XW} = 0, b_{XW} = 3, c_{XW} = 3, a_{WX} = 0.2, b_{WX} = 5, c_{WX} = 0$. (g) $\alpha \to \infty$. Parameters: $a_{XY} = 0, b_{XY} = 0, c_{XY} = 0, a_{XW} = 0.6, b_{XW} = 3, c_{XW} = 5, a_{WX} = 0.2, b_{WX} = 5, c_{WX} = 2$. (h) Nullclines intersect at $z > 1$. Parameters: $a_{XY} = 0, b_{XY} = 0, c_{XY} = 0.1, a_{XW} = 0.6, b_{XW} = 3, c_{XW} = 5, a_{WX} = 0.2, b_{WX} = 1, c_{WX} = 0$.

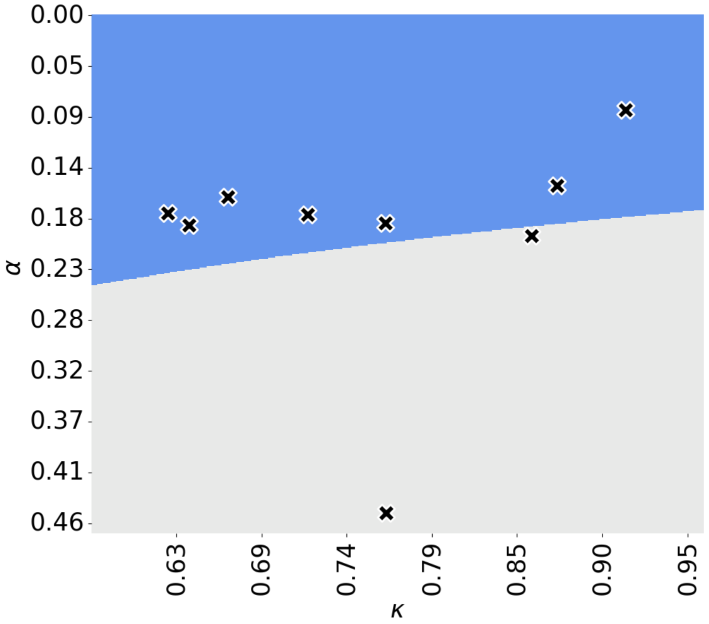

Figure SI.2: Voting experiments cluster near the theoretical boundary between mono- and bistability. Phase plot for the voting game experiments, showing zones of mono (grey) and bistability (blue). The crosses show the location of different voting experiments in this phase space (see SI Sections SI.5 and SI.6 for details). For other 3 experiments, either $\alpha > 1$ or $\alpha < 0$, indicating monostability. While most of the experiments show bistability in the deterministic limit, they are close to the boundary for monostability.

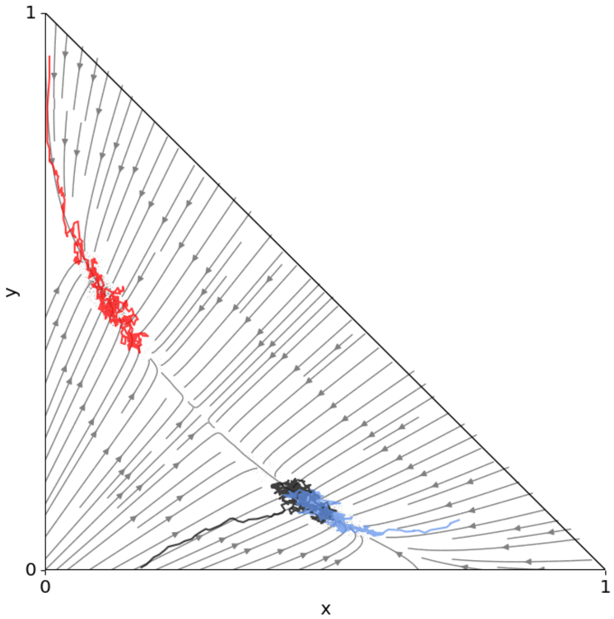

Figure SI.3: Consensus dynamics in the simplex rapidly collapse onto a low-dimensional slow manifold, where fluctuations drive transitions between consensus states. Grey arrows show the deterministic flow field given by the drift in Eq. (SI.1.2). Trajectories of stochastic simulations of the system (colours) quickly converge to a slow manifold following the directions of the flow field. Fluctuations push the system outside the submanifold, only for it to be carried back by the outer flow field.

SI.1.1 Slow manifold dynamics

In the approach defined by Katzenberger [1990], the system under study has the form

$$\frac{\mathrm{d}\boldsymbol{x}(t)}{\mathrm{d}t} = \mathbf{f}(\boldsymbol{x}) + \varepsilon \mathbf{h}(\boldsymbol{x}) + \sqrt{\mu}\mathbf{G}(\boldsymbol{x})\boldsymbol{\eta}(t), \tag{SI.1.10}$$

where ε is a small parameter that gives the slower timescale, and μ is the strength of the noise. Furthermore, the function $\mathbf{f}(\boldsymbol{x})$ defines the submanifold to which the system converges due to the fast timescale, i.e., $\mathbf{f}(\tilde{\boldsymbol{x}}) = \mathbf{0}$ for all points $\tilde{\boldsymbol{x}}$ in the submanifold, $\tilde{\boldsymbol{x}} \in \Gamma$.

If ε and μ are small, then the system is quickly carried to the manifold Γ by the outer flow $\mathbf{f}$. After this initial transient, stochastic fluctuations carry the system away from the manifold, only for it to return rapidly via the paths described by the flow $\mathbf{f}$. The goal is to describe the dynamics of the variable $\tilde{\boldsymbol{x}}$ in the slow manifold.

Parsons and Rogers [2017] consider the deterministic initial value problem,

$$\begin{cases} \frac{\mathrm{d}\boldsymbol{\xi}_{\boldsymbol{x}}(t)}{\mathrm{d}t} = \mathbf{f}(\boldsymbol{\xi}_{\boldsymbol{x}}(t)), \\ \boldsymbol{\xi}_{x}(0) = \boldsymbol{x}, \end{cases} \tag{SI.1.11}$$

and define the endpoint of the deterministic trajectories

$$\boldsymbol{\pi}(\boldsymbol{x}) = \lim_{t\to\infty} \boldsymbol{\xi}_x(t). \tag{SI.1.12}$$

Since all deterministic trajectories end at Γ, $\boldsymbol{\pi}$ is restricted to the manifold. Furthermore, taking the current position of the random variable $\boldsymbol{x}$ with dynamics given by Eq. (SI.1.10), $\boldsymbol{\pi}(\boldsymbol{x})$ will also be a random variable which tracks the projection of $\boldsymbol{x}$ into Γ. The authors then apply Itô's formula to obtain,

$$\frac{\mathrm{d}\pi_i}{\mathrm{d}t} = \varepsilon \sum_j \frac{\partial \pi_i}{\partial x_j} h_j(\boldsymbol{x}) + \frac{\mu}{2} \sum_{s,j,k} G_{js}(\boldsymbol{x}) G_{ks}(\boldsymbol{x}) \frac{\partial^2 \pi_i}{\partial x_i \partial x_j} + \sqrt{\mu} \sum_{s,j} G_{js}(\boldsymbol{x}) \frac{\partial \pi_i}{\partial x_j} \eta_s(t).$$

The above expression is not closed, since to write π as a function of t it is necessary to have full knowledge of $\boldsymbol{x}(t)$. To circumvent this problem, the authors consider the variable $\tilde{\boldsymbol{x}} \in \Gamma$ to be a close approximation of both $\boldsymbol{x}$ and $\boldsymbol{\pi}(\boldsymbol{x})$. This assumption is justified if the fluctuations do not carry $\boldsymbol{x}$ too far away from the manifold Γ each time. Finally, the authors substitute this into the previous equation, obtaining

$$\frac{\mathrm{d}\tilde{x}_i}{\mathrm{d}t} = \varepsilon \sum_j \mathbf{P}_{ij}(\tilde{\boldsymbol{x}}) h_j(\tilde{\boldsymbol{x}}) + \frac{\mu}{2} \sum_{s,j,k} G_{js}(\tilde{\boldsymbol{x}}) G_{ks}(\tilde{\boldsymbol{x}}) Q_{ijk}(\tilde{\boldsymbol{x}}) + \sqrt{\mu} \sum_{s,j} \mathbf{P}_{ij}(\tilde{\boldsymbol{x}}) G_{js}(\tilde{\boldsymbol{x}}) \eta_s(t),$$

where

$$\mathbf{P}_{ij} = \left.\frac{\partial \pi_i}{\partial x_j}\right|_{\boldsymbol{x}=\tilde{\boldsymbol{x}}}, \qquad Q_{ijk} = \left.\frac{\partial^2 \pi_i}{\partial x_j \partial x_k}\right|_{\boldsymbol{x}=\tilde{\boldsymbol{x}}}.$$

Details of the derivation of the previous equations and examples can be found in [Parsons and Rogers, 2017].

Following this approach, our first step is to determine all submanifolds permitted by the dynamics of your system by analysing the deterministic part of Eq. (SI.1.2). After determining the possible manifolds, we can then derive the lower-dimensional stochastic dynamics in the manifolds. For completeness and to provide a better perspective of our results and their impact, we rewrite the drift more generally as

$$\begin{aligned} \dot{x} &= Ax + Bxy + Cxw + Dy + Ew + Fyw, \\ \dot{y} &= Dx + Bxy + Fxw + Ay + Ew + Cyw, \end{aligned} \tag{SI.1.13}$$

where $w = 1 - x - y$. The coefficients relate to the parameters s, r and c as

$$\begin{aligned} A &= -a_{XY} - a_{XW}, & B &= -c_{XW}, & C &= -c_{XY} - b_{XW} + b_{WX}, \\ D &= a_{XY}, & E &= a_{WX}, & F &= c_{XY} + c_{WX}. \end{aligned}$$

In what follows, we demonstrate the existence of three possible straight lines and a family of curved submanifolds of fixed points, obtainable if no assumptions are made on the coefficients A, B, C, D, E, F. Then, we show that the relationship between the coefficients and the rates a_{XY}, b_{XY}, c_{XY} restricts the choice of physical manifolds to either a straight line (type I) or a curved (type II).

SI.1.2 Straight-line manifolds (type I)

We look for fixed-point solutions of our system of equations, (SI.1.13), which lie in a lower dimensional manifold. We first look for fixed-point solutions of the form

$$\begin{aligned} \dot{x} &= v_1 f(x, y, w) = 0, \\ \dot{y} &= v_2 f(x, y, w) = 0, \end{aligned} \tag{SI.1.14}$$

for some $v_1, v_2 \in \{x, y, w\}$ and function $f(x, y, w)$ we want to find. Because $\dot{x}$ is at most quadratic, $f(x, y, w)$ can be at most linear on x, y, w. Hence, our manifolds are planes, which become lines when restricted to the plane $w = 1 - x - y$.

Manifold I.1: First, we take $v_1 = x$, $v_2 = y$. Hence,

$$\begin{aligned} \dot{x} &= x(A + By + Cw) + Dy + Ew + Fyw = 0, \\ \dot{y} &= y(A + Bx + Cw) + Dx + Ew + Fxw = 0. \end{aligned}$$

Thus, to get a submanifold of fixed points of the form given in equations (SI.1.14) we need $B = D = E = F = 0$, giving $f(x, y, w) = A + Cw = 0$. This gives the submanifold

$A + Cw = 0$, or $w = -\frac{A}{C}$ constant. Assuming fixed population size, $w = 1 - x - y$, the number of neutral actors is constant. From the relationship between the parameters and rates, $A \leq 0$, while C can be either positive or negative, meaning that the feasibility of this submanifold is dependent on parameter values. Note that, in this case, $\frac{\mathrm{d}y}{\mathrm{d}x} = \frac{y}{x}$. Hence, the fast-flow lines are of the form $y = Kx$, for $K \in \mathbb{R}$.

Manifold I.2: Taking $v_1 = y$, $v_2 = x$,

$$\begin{aligned}\dot{x} &= y(D + Bx + Fw) + Ax + Ew + Cxw = 0,\\ \dot{y} &= x(D + By + Fw) + Ay + Ew + Cyw = 0.\end{aligned}$$

Similarly, we need $A = B = C = E = 0$. Therefore, the manifold is $D + Fw = 0$, or $w = 1 - x - y = -\frac{D}{F}$. Since $D, F \geq 0$, it follows that $w < 0$, rendering this submanifold not physically possible. Although this submanifold also gives the number of neutral actors as a constant of the system, the fast-flow lines are different. In this case, $\frac{\mathrm{d}y}{\mathrm{d}x} = \frac{x}{y}$, i.e, $x^2 - y^2 = K$ for $K \in \mathbb{R}$.

Manifold I.3: Taking $v_1 = w$, $v_2 = w$,

$$\begin{aligned}\dot{x} &= w(E + Cx + Fy) + Ax + Bxy + Dy = 0,\\ \dot{y} &= w(E + Fx + Cy) + Dx + Bxy + Ay = 0.\end{aligned}$$

For this, we need $A = B = D = 0$ and $C = F$. The manifold is then $E + Fx + Fy = 0$, or $w = 1 - x - y = 1 + \frac{E}{F}$. Since $E, F \geq 0$, it follows that $w \geq 1$, rendering this submanifold not physically attainable in our system either. The fast-flow lines are now $\frac{\mathrm{d}y}{\mathrm{d}x} = 1$, i.e., $y = x + K$, for $K \in \mathbb{R}$.

Any other combination of $v_1, v_2 \in \{x, y, w\}$ is a special case of one of the other manifolds.

SI.1.3 Curved manifolds (type II)

We now look for fixed-point manifold solutions in the system when $\dot{x} = \dot{y}$, i.e.,

$$\begin{aligned}\dot{x} &= Ax + Bxy + Cxw + Dy + Ew + Fyw = 0,\\ \dot{y} &= Dx + Bxy + Fxw + Ay + Ew + Cyw = 0.\end{aligned} \tag{SI.1.15}$$

To have $\dot{x} = \dot{y}$, we need $A = D$ and $C = F$. Substituting $w = 1 - x - y$, into Eqs. (SI.1.13), the resulting manifold, in terms of the variables x and y, is the conic curve

$$Fx^2 - (B - 2F)xy + Fy^2 - (A - E + F)x - (A - E + F)y - E = 0. \tag{SI.1.16}$$

If $(B - 2F)^2 - 4F^2 \leq 0$, the manifold is an ellipse. If $(B - 2F)^2 - 4F^2 = 0$, it is a parabola, and if $(B - 2F)^2 - 4F^2 \geq 0$, it is a hyperbola. Additionally, if $(B - 2F) = 0$, then it is

a circle. In fact, if $B = 0$, then using $x + y = 1 - w$ in equation (SI.1.13), it follows that $Ax + Fyw + Ay + Fxw + Ew = A(1 - w) + Fw(1 - w) + Ew = 0$, which is a quadratic equation for w that can be solved to obtain

$$w_{\pm} = \frac{(F + E - A) \pm \sqrt{(F + E - A)^2 + 4AF}}{2F}.$$

Hence, provided that the solutions are real we have that, $1 - x - y = w_{\pm}$, so our manifold corresponds to two parallel straight lines rather than parabolas. Note that, this is true only because we assumed fixed population size, i.e., $w = 1 - x - y$.

We note that $(B - 2F)^2 - 4F^2 = B^2 - 4BF = B(B - 4F)$. Since $B = -c_{XW} \leq 0$ and $F = c_{XY} + c_{WX} \geq 0$, it follows that $(B - 2F)^2 \geq 0$ and the only physically curved manifold is a hyperbola.

In summary, imposing no restrictions on the coefficients A, B, C, D, E and F, and only assuming symmetry of X and Y and linear responses to the state of the system, there are three line manifolds with constant w, but different fast/slow parameters and flows, and a family of curved manifolds which can be any conic section. However, once relationships between the coefficients and the rates are considered. Only one straight-line manifold and a hyperbola become physically attainable in our system. We highlight that the other manifolds could be achieved if rates depend on the number of particles in the system – or are allowed to be negative within some constraints not to render transition rates negative.

SI.1.4 General straight submanifold dynamics

Now that we have found our two physical submanifolds, we can take the next step in the approach used by Parsons and Rogers [2017] and restrict the stochastic dynamics onto the slow manifolds to obtain a lower-dimensional description of the system. For the straight-line manifold, we require $B = D = E = F = 0$. Let $r' = b_{WX} - b_{XW}$, we rescale time by $t \mapsto r't$. Define, $\frac{2a_{WX}}{r'} = \varepsilon\bar{\beta}_1$, $\frac{2a_{XY}}{r'} = \varepsilon\bar{\beta}_2$, $\frac{c_{XW}}{2r'} = \varepsilon\bar{\gamma}_1$, $\frac{2c_{XY}}{r'} = \varepsilon\bar{\gamma}_3$, $\frac{c_{WX}}{r'} = \varepsilon\bar{\gamma}_2$ and $\frac{a_{XW}}{r'} = \bar{\alpha}$. Note that α defined in Eq. (SI.1.8) corresponds to $\bar{\alpha}$ plus a correction of order ε, so in the limit $\varepsilon \to 0$, α and $\bar{\alpha}$ are equivalent. Additionally, $\kappa = \frac{\varepsilon\bar{\beta}_1}{4\varepsilon\bar{\gamma}_1}$, for κ was defined in Eq. (SI.1.9).

We note that the over bar notation represents parameters defined for the straight manifold and not an expectation. In the next section, we will define parameters for the hyperbolic manifold that have similar meaning, but will use a hat notation to show those are specific to the curved manifold.

We can then write our system in the form

$$\frac{\mathrm{d}\boldsymbol{z}(t)}{\mathrm{d}t} = \boldsymbol{f}(\boldsymbol{z}) + \varepsilon\boldsymbol{h}(\boldsymbol{z}) + \sqrt{\mu}\bar{\boldsymbol{G}}(\boldsymbol{z})\boldsymbol{\eta}(t),$$

with

$$\boldsymbol{f}(\boldsymbol{z}) = \begin{pmatrix} z\{1-u-\bar{\alpha}\} \\ u\{1-u-\bar{\alpha}\} \end{pmatrix},$$

$$\boldsymbol{h}(\boldsymbol{z}) = \begin{pmatrix} z\{\bar{\beta}_2 - (\bar{\gamma}_3 + \bar{\gamma}_2)(1-u)\} \\ \{\bar{\beta}_1(1-u) - \bar{\gamma}_1(u^2 - z^2) + \bar{\gamma}_2 u(1-u)\} \end{pmatrix},$$

for $\boldsymbol{z} = (z, u)$, and

$$\bar{\boldsymbol{G}}(\boldsymbol{z}) = \begin{pmatrix} G_{11} & -G_{22} & 2G_{13} \\ G_{11} & G_{22} & 0 \end{pmatrix},$$

for G_{11}, G_{22} and G_{13} defined in Eq. (SI.1.3). Following the method by Parsons and Rogers [2017], the submanifold is defined by the points $(\tilde{z}, \tilde{u})$ in the simplex such that

$$1 - \tilde{u} - \bar{\alpha} = 0 \Rightarrow \tilde{u} = 1 - \bar{\alpha}. \tag{SI.1.17}$$

In the limit $\varepsilon \to 0$, $dz/du \to z/u$. Hence, the outer flow consists of radial lines $u = Kz$, $K \in \mathbb{R} \setminus (-1, 1)$. It is straightforward to write the projection of a point $\boldsymbol{z} = (z, u)$ into the manifold by the outer flow,

$$\boldsymbol{\pi}(\boldsymbol{z}) = \begin{pmatrix} (1-\bar{\alpha})\frac{z}{u} \\ (1-\bar{\alpha}) \end{pmatrix}. \tag{SI.1.18}$$

We may choose to describe our one-dimensional manifold by its coordinate $\tilde{z}$. The derivatives of $\pi_1(z, u)$ are then,

$$\frac{\partial \pi_1}{\partial z} = 1, \quad \frac{\partial \pi_1}{\partial u} = -\frac{z}{1-\bar{\alpha}}, \quad \frac{\partial^2 \pi_1}{\partial z^2} = 0, \quad \frac{\partial^2 \pi_1}{\partial z \partial u} = -\frac{1}{(1-\bar{\alpha})}, \quad \frac{\partial^2 \pi_1}{\partial u^2} = \frac{2z}{(1-\bar{\alpha})^2}.$$

Therefore,

$$\begin{aligned} \frac{\mathrm{d}\tilde{z}}{\mathrm{d}t} &= \varepsilon \tilde{z} \left\{ \bar{\beta}_2 - (\bar{\gamma}_3 + 2\bar{\gamma}_2) - \frac{\bar{\beta}_1 \bar{\alpha}}{(1-\bar{\alpha})} + \frac{\bar{\gamma}_1}{(1-\bar{\alpha})}[(1-\bar{\alpha})^2 - \tilde{z}^2] \right\} \\ &+ \frac{\varepsilon}{N} \frac{\tilde{z}}{(1-\bar{\alpha})^2} \left\{ \bar{\beta}_1 \bar{\alpha} + \bar{\gamma}_1[(1-\bar{\alpha})^2 - \tilde{z}^2] + 2\bar{\gamma}_2(1-\bar{\alpha}) \right\} \\ &+ \frac{1}{\sqrt{N}} \left\{ \frac{(1-\bar{\alpha}-\tilde{z})}{(1-\bar{\alpha})} G_{11}\eta_1(t) - \frac{(1-\bar{\alpha}+\tilde{z})}{(1-\bar{\alpha})} G_{22}\eta_2(t) + 2G_{13}\eta_3(t) \right\}. \end{aligned} \tag{SI.1.19}$$

Hence, we have a complete description of the dynamics on the submanifold in terms of a single variable $\tilde{z}$. Observe the emergence of effective three-body interactions in the slower dynamics (cubic terms) despite the absence of three-body interactions in the model.

We can derive its stationary probability distribution (SPD). Firstly, we write the equation in the form

$$\frac{d\tilde{z}}{dt} = F(\tilde{z}) + \sqrt{2D(\tilde{z})}\eta(t), \tag{SI.1.20}$$

with

$$
\begin{aligned}
F(\tilde{z}) =& \tilde{z}\{-c_1 + [(1-\bar{\alpha})^2 - \tilde{z}^2]c_2\},\\
D(\tilde{z}) =& [(1-\bar{\alpha})^2 - \tilde{z}^2]d_1,
\end{aligned}
\tag{SI.1.21}
$$

and coefficients

$$
\begin{aligned}
c_1 &= \frac{\varepsilon\bar{\beta}_1\bar{\alpha}}{(1-\bar{\alpha})} - \varepsilon\bar{\beta}_2 + \varepsilon\bar{\gamma}_3 + 2\varepsilon\bar{\gamma}_2 - \frac{\varepsilon}{N}\frac{1}{(1-\bar{\alpha})^2}\left\{\bar{\beta}_1\bar{\alpha} + 2\bar{\gamma}_2(1-\bar{\alpha})\right\},\\
c_2 &= \frac{\varepsilon\bar{\gamma}_1}{(1-\bar{\alpha})}\left(1 + \frac{1}{N(1-\bar{\alpha})}\right),\\
d_1 &= \frac{1}{2N}\left(\frac{\bar{\alpha}(1+\bar{\delta})}{(1-\bar{\alpha})} + 2\bar{\xi}\right),
\end{aligned}
\tag{SI.1.22}
$$

for $\bar{\delta} = \frac{b_{WX}+b_{XW}}{b_{WX}-b_{XW}}$ and $\bar{\xi} = \frac{b_{XY}}{b_{WX}-b_{XW}}$.

The stationary probability distribution is

$$
p_s(\tilde{z}) = \frac{C}{D(\tilde{z})}\exp\left\{\int_{-1}^{\tilde{z}}\frac{F(y)}{D(y)}\mathrm{d}y\right\},
\tag{SI.1.23}
$$

where C is a normalisation constant. In this case, we are able to solve the integral, leading to

$$
p_s(\tilde{z}) = C[(1-\bar{\alpha})^2 - \tilde{z}^2]^{\frac{c_1}{2d_1}-1}\exp\left\{-\frac{c_2}{2d_1}[(1-\bar{\alpha})^2 - \tilde{z}^2]\right\},
\tag{SI.1.24}
$$

for $\tilde{z} \in [\bar{\alpha}-1, 1-\bar{\alpha}]$. Correspondingly, the stationary probability distribution can be written as $p_s(\tilde{z}) \propto \exp\left\{-\bar{\Phi}(\tilde{z})\right\}$, where the potential, $\bar{\Phi}(\tilde{z})$, is given by

$$
\bar{\Phi}(\tilde{z}) = -\left(\frac{c_1}{2d_1} - 1\right)\log\left[(1-\bar{\alpha})^2 - \tilde{z}^2\right] + \frac{c_2}{2d_1}[(1-\bar{\alpha})^2 - \tilde{z}^2].
$$

Differentiating $\bar{\Phi}(\tilde{z})$ and equating to zero gives the maxima ($\tilde{z}_\pm$) and minimum ($\tilde{z}_u$) of the SPD,

$$
\tilde{z}_\pm = \pm\sqrt{(1-\bar{\alpha})^2 - \frac{c_1}{c_2} + \frac{2d_1}{c_2}}, \qquad \tilde{z}_u = 0.
\tag{SI.1.25}
$$

These match with the deterministic fixed points found in Eq. (SI.1.8) when $d_1 = 0$, i.e., when $N \to \infty$, as expected.

Note that, depending on the population size, N, the values of $\tilde{z}_\pm$ are not within the interval $(\bar{\alpha}-1, 1-\bar{\alpha})$, in which case the SPD is U-shaped, and the favourable system states are at the edges of the interval. However, if $\frac{c_1}{2d_1} - 1 > 0$, then $\tilde{z}_\pm$ is in the interval $(\bar{\alpha}-1, 1-\bar{\alpha})$. Fig. SI.4 illustrates this picture. This is the same condition that guarantees that the term $[(1-\bar{\alpha})^2 - \tilde{z}^2]^{\frac{c_1}{2d_1}-1}$ in Eq. (SI.1.24) does not diverge at $\tilde{z} = \pm(1-\bar{\alpha})$.

By computing the mean switching time [Gardiner, 1985] between the two favourable states

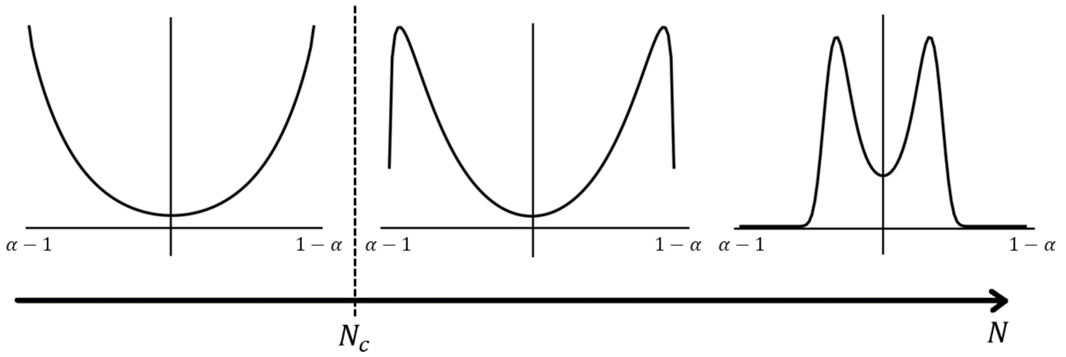

Figure SI.4: The behaviour of the stationary probability distribution as N increases. If N_c is the value of N such that $\frac{c_1}{2d_1} - 1 = 0$, then for $N < N_c$, the noise pushes the favourable system states to $z = \pm(1-\alpha)$ and the SPD is 'U-shaped'. For $N > N_c$, a transition occurs, and the SPD becomes 'M-shaped', with $z_\pm$ inside the simplex.

of the system, we can obtain insight into how frequently fluctuations can lead to swaps in consensus. The mean switching time is given by

$$\bar{\tau} = 2\tau(\tilde{z}_-) = 2\int_{\tilde{z}_-}^{\tilde{z}_u} \frac{1}{D(z)p_s(z)} \int_{\bar{\alpha}-1}^{z} p_s(y)\mathrm{d}y\mathrm{d}z, \quad \text{(SI.1.26)}$$

where $\tilde{z}_-$ and $\tilde{z}_u$ are given as above, and $p_s(\tilde{z})$ and $D(\tilde{z})$ are as in Eq. (SI.1.25) and Eq. (SI.1.21), respectively. While this integral is not trivial to solve, it can be evaluated numerically as in Figure 1 (g) (iii) of the main text.

SI.1.5 General curved submanifold dynamics

The conditions for the curved submanifold are $A = D$ and $C = F$. This implies that $\frac{a_{XW}+2a_{XY}}{a_{WX}} = \varepsilon\hat{\alpha}$ and $\frac{b_{WX}-b_{XW}-2c_{XY}-c_{WX}}{a_{WX}} = \varepsilon\hat{\beta}$, for an infinitesimal small ε. Note that $\alpha = \frac{\varepsilon\hat{\alpha}}{\varepsilon\hat{\beta}}$, for α defined in Eq. (SI.1.8), does not need to be infinitesimal.

If we rescale time, $t \mapsto a_{WX}t$, we can then write our system in the form

$$\frac{\mathrm{d}\boldsymbol{z}(t)}{\mathrm{d}t} = \boldsymbol{f}(\boldsymbol{z}) + \varepsilon\boldsymbol{h}(\boldsymbol{z}) + \sqrt{N^{-1}}\hat{\boldsymbol{G}}(\boldsymbol{z})\boldsymbol{\eta}(t),$$

with

$$\boldsymbol{f}(\boldsymbol{z}) = \begin{pmatrix} 0 \\ 2\{(1-u) - \frac{\hat{\gamma}}{4}(u^2 - z^2) + \hat{\delta}u(1-u)\} \end{pmatrix}, \qquad \boldsymbol{h}(\boldsymbol{z}) = \begin{pmatrix} z\{-\hat{\alpha} + \hat{\beta}(1-u)\} \\ -\hat{\alpha}_{XW}u \end{pmatrix},$$

for $\boldsymbol{z} = (z, u)$, $\hat{\gamma} = \frac{c_{XW}}{a_{WX}}$ (equivalent to κ^{-1} in Eq. (SI.1.9)), $\hat{\delta} = \frac{b_{WX}-b_{XW}+c_{WX}}{2a_{WX}}$, $\varepsilon\hat{\alpha}_{XW} = \frac{a_{XW}}{a_{WX}}$, and

$$\hat{\boldsymbol{G}}(\boldsymbol{z}) = \begin{pmatrix} G_{11} & -G_{22} & 2G_{13} \\ G_{11} & G_{22} & 0 \end{pmatrix},$$

for $\boldsymbol{G_1}$ and $\boldsymbol{G_2}$ defined in Eq. (SI.1.3). Following the method by Parsons and Rogers [2017], the submanifold is defined by the points $(\tilde{z}, \tilde{u})$ such that $1 - \tilde{u} - \frac{\hat{\gamma}}{4}(\tilde{u}^2 - \tilde{z}^2) + \hat{\delta}\tilde{u}(1 - \tilde{u}) = 0$ or

$$\tilde{u} = \tfrac{1}{\hat{\gamma}+4\hat{\delta}} \left\{ 2(\hat{\delta} - 1) + \sqrt{4(\hat{\delta} - 1)^2 + (\hat{\gamma} + 4\hat{\delta})(4 + \hat{\gamma}\tilde{z}^2)} \right\}. \tag{SI.1.27}$$

In the limit $\varepsilon \to 0$, $dz/dt \to 0$. The outer flow is then parallel to the lines $z = z_0$, $z_0 \in [-1, 1]$. It is straightforward to write the projection of a point $\boldsymbol{z} = (z, u)$ into the manifold by the outer flow,

$$\boldsymbol{\pi}(\boldsymbol{z}) = \begin{pmatrix} z \\ \tfrac{1}{\hat{\gamma}+4\hat{\delta}} \left\{ 2(\hat{\delta} - 1) + \sqrt{4(\hat{\delta} - 1)^2 + (\hat{\gamma} + 4\hat{\delta})(4 + \hat{\gamma}\tilde{z}^2)} \right\} \end{pmatrix}.$$

We may choose to describe our one-dimensional manifold by its coordinate $\tilde{z}$. The derivatives of $\pi_1(z, u)$ are then,

$$\frac{\partial \pi_1}{\partial z} = 1, \qquad \frac{\partial \pi_1}{\partial u} = 0, \qquad \frac{\partial^2 \pi_1}{\partial z^2} = \frac{\partial^2 \pi_1}{\partial u^2} = \frac{\partial^2 \pi_1}{\partial z \partial u} = 0.$$

Therefore, $\boldsymbol{P}_1 = (1, 0)^T$, and $\boldsymbol{Q} = 0$. Thus, the dynamics over the manifold are

$$\begin{aligned} \frac{\mathrm{d}\tilde{z}}{\mathrm{d}t} =& \varepsilon\tilde{z} \left\{ \hat{\beta} - \tfrac{\hat{\beta}}{\hat{\gamma}+4\hat{\delta}} \left[2(\hat{\delta} - 1) + \sqrt{4(\hat{\delta} - 1)^2 + (\hat{\gamma} + 4\hat{\delta})(4 + \hat{\gamma}\tilde{z}^2)} \right] - \hat{\alpha} \right\} \\ &+ \frac{1}{\sqrt{N}} \{ G_{11}\eta_1(t) - G_{22}\eta_2(t) + 2G_{13}\eta_3(t) \}. \end{aligned} \tag{SI.1.28}$$

Hence, we have a complete description of the dynamics on the submanifold in terms of a single variable $\tilde{z}$. Writing

$$\frac{d\tilde{z}}{dt} = F(\tilde{z}) + \sqrt{2D(\tilde{z})}\eta(t), \tag{SI.1.29}$$

with $F(\tilde{z}) = \tilde{z}\{\varepsilon\hat{\beta}[1 - \tilde{u}(\tilde{z})] - \varepsilon\hat{\alpha}\}$ and $D(\tilde{z}) = \hat{d}_1[1 - \tilde{u}(\tilde{z})] + \hat{d}_2\tilde{u}(\tilde{z})[1 - \tilde{u}(\tilde{z})]$ for

$$\begin{aligned} \hat{d}_1 &= \frac{2}{N} \left(1 + \frac{2\hat{\xi}}{\hat{\gamma}} \right), \\ \hat{d}_2 &= \frac{1}{N} \left(\left[1 + \frac{4\hat{\xi}}{\hat{\gamma}} \right] \hat{\delta} + \hat{\vartheta} + \hat{\varsigma} \right), \end{aligned} \tag{SI.1.30}$$

where $\hat{\xi} = \frac{b_{XY}}{a_{WX}}$, $\hat{\vartheta} = \frac{2c_{XY}}{a_{WX}}$ and $\hat{\varsigma} = \frac{b_{WX}+b_{XW}+c_{WX}}{2a_{WX}}$. Note that $\tilde{u}(\tilde{z})$ is the variable defined over the submanifold in Eq. (SI.1.27).

As before, the stationary probability distribution is

$$p_s(\tilde{z}) = \frac{C}{D(\tilde{z})} \exp\left\{ \int_{-1}^{\tilde{z}} \frac{F(y)}{D(y)} \mathrm{d}y \right\},$$

where C is a normalisation constant. We compute,

$$\int_{-1}^{z} \frac{F(y)}{D(y)} \mathrm{d}y = \int_{-1}^{z} \frac{\varepsilon\hat{\beta}y}{\hat{d}_1 + \hat{d}_2\tilde{u}(y)} \mathrm{d}y - \int_{-1}^{z} \frac{\varepsilon\hat{\alpha}y}{(\hat{d}_1 + \hat{d}_2\tilde{u}(y))(1-\tilde{u}(y))} \mathrm{d}y.$$

Changing variables,

$$\int \frac{\varepsilon\hat{\beta}y}{\hat{d}_1 + \hat{d}_2\tilde{u}(y)} \mathrm{d}y = \varepsilon\hat{\beta} \int \frac{\tilde{u}(1+\frac{4\hat{\delta}}{\hat{\gamma}}) - \frac{2}{\hat{\gamma}}(\hat{\delta}-1)}{\hat{d}_1 + \hat{d}_2\tilde{u}} \mathrm{d}\tilde{u},$$

$$\int \frac{\varepsilon\hat{\alpha}y}{(\hat{d}_1 + \hat{d}_2\tilde{u}(y))(1-\tilde{u}(y))} \mathrm{d}y = \varepsilon\hat{\alpha} \int \frac{\tilde{u}(1+\frac{4\hat{\delta}}{\hat{\gamma}}) - \frac{2}{\hat{\gamma}}(\hat{\delta}-1)}{(\hat{d}_1 + \hat{d}_2\tilde{u})(1-\tilde{u})} \mathrm{d}\tilde{u}.$$

Performing the integrations and combining terms we find that the stationary probability distribution is given by $p_s(\tilde{z}) = C\exp\left\{-\hat{\Phi}(\tilde{z})\right\}$, for some constant C, where this time the potential, $\hat{\Phi}(\tilde{z})$, is given by

$$\begin{aligned}\hat{\Phi}(\tilde{z}) = &\left\{\left[\hat{d}_1\left(1+\frac{4\hat{\delta}}{\hat{\gamma}}\right) + \frac{2\hat{d}_2(\hat{\delta}-1)}{\hat{\gamma}}\right]\left[\frac{\varepsilon\hat{\beta}}{\hat{d}_2^2} - \frac{\varepsilon\hat{\alpha}}{\hat{d}_2(\hat{d}_1+\hat{d}_2)}\right] + 1\right\}\log[\hat{d}_1 + \hat{d}_2\tilde{u}(\tilde{z})] \\ &- \left[\frac{\varepsilon\hat{\alpha}}{\hat{d}_1+\hat{d}_2}\left(1+\frac{2(\hat{\delta}+1)}{\hat{\gamma}}\right) - 1\right]\log[1-\tilde{u}(\tilde{z})] - \frac{\varepsilon\hat{\beta}}{\hat{d}_2}\left(1+\frac{4\hat{\delta}}{\hat{\gamma}}\right)\tilde{u}(\tilde{z}), \\ = &-\hat{\phi}_1\tilde{u}(\tilde{z}) + \hat{\phi}_2\log[\hat{d}_1 + \hat{d}_2\tilde{u}(\tilde{z})] - \hat{\phi}_3\log[1-\tilde{u}(\tilde{z})]. \end{aligned} \tag{SI.1.31}$$

The extrema of the SPD are the solutions of

$$\frac{d\hat{\Phi}(\tilde{z})}{d\tilde{z}} = \left(-\hat{\phi}_1 + \frac{\hat{\phi}_2\hat{d}_2}{\hat{d}_1+\hat{d}_2\tilde{u}} - \frac{\hat{\phi}_3}{1-\tilde{u}}\right)\frac{d\tilde{u}}{d\tilde{z}} = 0. \tag{SI.1.32}$$

Note that $d\tilde{u}/d\tilde{z} = 0 \Rightarrow \tilde{z} = 0$, while equating the terms in brackets to 0 gives another two solutions for $\tilde{z}$.

In both our applications to voting and locusts experimental data (see SI Sections SI.5 and SI.3, respectively), we observe c_{XY} and c_{WX} to be small, and consequently, $\hat{\delta}, \hat{\vartheta}, \hat{\varsigma}$ are of order ε. Additionally, the locust case has $\hat{\xi} = 0$, and consequently $\hat{d}_2 = 0$. The SPD to first-order is then,

$$p_s(\tilde{z}) \propto \left\{\hat{\gamma} + 2 - \sqrt{4+4\hat{\gamma}+\hat{\gamma}^2\tilde{z}^2}\right\}^{\frac{\varepsilon\hat{\alpha}N(2+\hat{\gamma})}{2\hat{\gamma}+4\hat{\xi}}-1} \exp\left\{\frac{\varepsilon\hat{\beta}N\hat{\gamma}\tilde{z}^2}{4\hat{\gamma}+8\hat{\xi}} + \frac{\varepsilon\hat{\alpha}N\sqrt{4+4\hat{\gamma}+\hat{\gamma}^2\tilde{z}^2}}{2\hat{\gamma}+4\hat{\xi}}\right\}. \tag{SI.1.33}$$

The extrema of the SPD are at $\tilde{z}_u = 0$ and

$$\tilde{z}_{\pm} = \pm\frac{1}{\sqrt{2}}\Bigg\{ (1-\alpha)^2 - \frac{4}{\hat{\gamma}^2} - 4\frac{(\alpha+1)}{\hat{\gamma}} + \frac{2(\hat{\gamma}+\hat{\gamma}_1)}{N\varepsilon\hat{\beta}\hat{\gamma}} + \left(1-\alpha+\frac{2}{\hat{\gamma}}\right)\sqrt{\left(1-\alpha+\frac{2}{\hat{\gamma}}\right)^2 + 4\frac{(\hat{\gamma}+\hat{\gamma}_1)}{N\varepsilon\hat{\beta}\hat{\gamma}}}\Bigg\}^{\frac{1}{2}}, \tag{SI.1.34}$$

for $\alpha = \frac{\varepsilon\hat{\alpha}}{\varepsilon\hat{\beta}}$, as noted previously. The fixed points $\tilde{z}_{\pm}$ converge to the fixed points of the drift when $N \to \infty$, agreeing with the result of Eq. (SI.1.9). As with the straight line manifold, if $N > N_c = \frac{\hat{\gamma}+\hat{\gamma}_1}{\varepsilon\hat{\alpha}(2+\hat{\gamma})}$, then the two maxima are in the interval $(-1, 1)$ (see Fig. SI.4). Otherwise, they are outside.

In this case, the potential $\hat{\Phi}(\tilde{z})$ becomes

$$\hat{\Phi}(\tilde{z}) = -\left(\frac{N\varepsilon\hat{\alpha}(2+\hat{\gamma})}{\hat{\gamma}+\hat{\gamma}_1} - 1\right)\log\left[\hat{\gamma}+2-\sqrt{4+4\hat{\gamma}+\hat{\gamma}^2 z^2}\right] - \frac{N\hat{\gamma}\varepsilon\hat{\beta}z^2}{2(\hat{\gamma}+\hat{\gamma}_1)} - \frac{N\varepsilon\hat{\alpha}}{(\hat{\gamma}+\hat{\gamma}_1)}\sqrt{4+4\hat{\gamma}+\hat{\gamma}^2 z^2}. \tag{SI.1.35}$$

The mean switching time is again given by

$$\hat{\tau} = 2\tau(\tilde{z}_-) = 2\int_{\tilde{z}_-}^{\tilde{z}_u} \frac{1}{D(z)p_s(z)} \int_{-1}^{z} p_s(y)\mathrm{d}y\mathrm{d}z, \tag{SI.1.36}$$

where $\tilde{z}_-$ and $\tilde{z}_u$ are given as above. While this integral is not trivial, it can be evaluated numerically as shown in 1 (g) (iii) of the main text.

SI.2 Spatial locust model

In the previous section, we have introduced a general mathematical framework in which collective decisions are made through interactions between agents. This mathematical framework ignores any spatial structure or correlations that could influence decisions locally. Nonetheless, for locusts in an arena, it can be argued that their decision to move clockwise, anticlockwise, or remain stationary can be influenced by local effects, such as aggregates of stopped locusts blocking their way, or whether they can only sense their nearest neighbours. With this in mind, Sections SM.2 and SM.4 have the purpose of convincing the reader that, at least for the dynamics of the experiments with locusts we are analysing, spatial effects can be neglected on the benefit of using the same space-free modelling approach of Section 1, which is much easier to analyse.

This is done by first deriving a spatial model and showing that the spatial model does not produce patterns (or aggregates) of particles, and deriving conditions for a well-mixed limit, namely, that the locusts move fast when compared to the timescale of the reactions, meaning

that they observe many other locusts before interacting with one at random. This is the goal of this section. In Section SM.4, we present data on the interaction radius of the locusts, their speed of propagation, and spatial correlations that confirm that the locust experiments are in a parameter regime for which the non-spatial model is a good approximation of the dynamics of the locust system.

In terms of spatial modelling of locusts in the annular arena, previous modelling approaches have considered the use of self-propelled particle models [Czirók et al., 1999, Yates et al., 2011, Bode et al., 2010]. Our model will be based on the $\lambda - \rho$ approach [Erban et al., 2007], in which there is an interaction radius ρ for each particle, but the interactions are not sure to happen every time two particles are within the interaction radius of each other. Rather, interactions occur with some rate λ. We will show that the well-mixed model is recovered in the limit that the interaction range tends to the size of the arena or when the speed of propagation of the locusts is fast compared to the timescale of the interactions. Importantly, we show that the spatially homogeneous distribution is stable, meaning no significant spatial patterns arise.

A note on notation: The notation in Section SI.2 contrasts with the remaining of the document to match the more familiar notation of x and y as spatial coordinates. We use R to denote an interaction radius, and r, ℓ and s to represent the proportions of clockwise, anticlockwise and stopped locusts, respectively.

SI.2.1 Movement dynamics

In our previous well-mixed model, we described the system by the number of "right" and "left" moving locusts. Spatial dynamics were neglected, meaning that every locust can interact with any other locust in the arena. To test whether spatial dynamics are crucial for the dynamics of the locust system, we will propose and study a spatial model.

Following our previous modelling choice, we take the movement of the locusts to the left or right to be deterministic and the changes in direction to be stochastic. This corresponds to a 'run-and-tumble' model [Rosser et al., 2013, Martens et al., 2012], often used to model the movement of animals [Codling et al., 2008], microorganisms [Berg, 2004, Sowa and Berry, 2008].

The usual framework to analyse systems that combine movement and stochastic interactions within a radius employs the definition and analysis of a functional master equation [Minors et al., 2018, Constable et al., 2016]. In this approach, we describe the dynamics of random fields $\{\phi^g(x,t)\}$ that give the spatial distribution of particles of different types, $\{g\}$.

Consider a system of N particles that move in a ring of length L. Let $x_i(t)$ be the position

of a particle $i \in \{1, \dots, N\}$, and

$$\phi(x,t) = \frac{1}{N} \sum_{i=1}^{N} \delta(x - x_i(t)), \tag{SI.2.1}$$

where $\delta(x)$ is the Dirac delta distribution, i.e., $\phi(x,t)$ is the density of particles at position x at time t. Similar to the non-spatial case, we can describe our system by a master equation, in this case, a functional master equation

$$\partial_t P(\phi) = N \int Q(\phi, x) P(\phi) \, \mathrm{d}x, \tag{SI.2.2}$$

where $P(\phi)$ is the probability of some spatial configuration $\phi : \phi(x)$, and $Q(\phi, x)$ is the field-equivalent to propensity functions.

In this picture, the particle jumps instantaneously from x to $x + \Delta x$ at time $t + \Delta t$. From a slightly different but equivalent perspective, the particle at x is annihilated, and a new particle is created at $x + \Delta x$ at time $t + \Delta t$. In the limit $\Delta t \to 0$, we obtain continuous motion. As in 'run-and-tumble' models, the particle might stochastically change direction, either spontaneously or as the result of interactions with other particles, which then changes the sign of Δx, i.e., $\Delta x \mapsto -\Delta x$.

Mathematically, $Q(\phi, x)$ will look like

$$Q(\phi, x) = \int (\Delta_x^- \Delta_y^+ - 1) K(x - y) \phi(y) \, \mathrm{d}y, \tag{SI.2.3}$$

where $\Delta_x^\pm$ are the creation/annihilation operators acting at position x and $K(x - y)$ is the transition kernel from going from y to x. The $\Delta_x^\pm$ operators, applied to a functional $F[\phi(x,t)]$, are defined as

$$\Delta_y^\pm F[\phi(x,t)] = F\left[\phi(x,t) \pm \frac{1}{N} \delta(x - y)\right]. \tag{SI.2.4}$$

For Brownian motion, the transition kernel is Gaussian. For deterministic movement, the positions are certain, corresponding to a delta distribution. If our particles move with speed v, they will move from x to $x + vh$ in a time interval h. In the limit that $h \to 0$, we get a continuous motion, i.e., our kernel is

$$K(x - y) = \lim_{h \to 0} \frac{1}{h} \delta([x + vh] - y). \tag{SI.2.5}$$

For finite but large N, we can perform a Kramers-Moyal (see, e.g., [Gardiner, 1985]) expansion of our operators

$$\Delta_x^\pm F[\phi(x,t)] = F[\phi(x,t)] \pm \frac{1}{N} \frac{\delta}{\delta \phi(x)} F[\phi(x,t)] + \frac{1}{2N^2} \frac{\delta^2}{\delta \phi(x)^2} F[\phi(x,t)] + \mathcal{O}(N^{-3}),$$

where $\frac{\delta}{\delta\phi}$ is the functional derivative with respect to the field ϕ. In the master equation, this becomes

$$\begin{aligned}\partial_t P(\phi) =& \int\int\left(\frac{\delta}{\delta\phi(y)} - \frac{\delta}{\delta\phi(x)}\right) K(x-y)\phi(y)P(\phi)\,\mathrm{d}y\,\mathrm{d}x \\ &+ \frac{1}{2N}\int\int\left(\frac{\delta}{\delta\phi(y)} - \frac{\delta}{\delta\phi(x)}\right)^2 K(x-y)\phi(y)\,\mathrm{d}y\,\mathrm{d}x.\end{aligned} \tag{SI.2.6}$$

Further analysis is better performed in Fourier space. Hence, let

$$\phi(x) = \sum_k \phi_k e^{ikx\frac{2\pi}{L}}, \quad \phi_k = \frac{1}{L}\int_0^L \phi(x)e^{-ikx\frac{2\pi}{L}}, \quad \frac{\delta}{\delta\phi(x)} = \frac{1}{2\pi}\sum_k \partial_{\phi_k} e^{-ikx\frac{2\pi}{L}}.$$

Focusing on the first integral,

$$\begin{aligned}&\int_0^L\int_0^L\left(\frac{\delta}{\delta\phi(y)} - \frac{\delta}{\delta\phi(x)}\right)K(x-y)\phi(y)P(\phi)\,\mathrm{d}y\,\mathrm{d}x \\ &= \frac{1}{L}\int_0^L\int_0^L\sum_{k,\ell}\partial_{\phi_k}\phi_\ell e^{i\ell x\frac{2\pi}{L}}\left(e^{-iky\frac{2\pi}{L}} - e^{-ikx\frac{2\pi}{L}}\right)K(x-y)P(\phi)\,\mathrm{d}y\,\mathrm{d}x, \\ &= \lim_{h\to 0}\frac{1}{Lh}\int_0^L\sum_{k,\ell}\partial_{\phi_k}\phi_\ell e^{i\ell x\frac{2\pi}{L}}\left(e^{-ik\frac{2\pi}{L}(x+vh)} - e^{-ikx\frac{2\pi}{L}}\right)P(\phi)\,\mathrm{d}x, \\ &= \lim_{h\to 0}\frac{1}{Lh}\int_0^L\sum_{k,\ell}\partial_{\phi_k}\phi_\ell e^{i(\ell-k)x\frac{2\pi}{L}}\left(-ikvh\tfrac{2\pi}{L} + \mathcal{O}(h^2)\right)P(\phi)\,\mathrm{d}x, \\ &= \sum_k \partial_{\phi_k}\left(-ikv\tfrac{2\pi}{L}\phi_k P(\phi)\right).\end{aligned}$$

Now, the second integral,

$$\begin{aligned}&\int_0^L\int_0^L\left(\frac{\delta}{\delta\phi(y)} - \frac{\delta}{\delta\phi(x)}\right)^2 K(x-y)\phi(y)P(\phi)\,\mathrm{d}y\,\mathrm{d}x \\ &= \frac{1}{L^2}\int_0^L\int_0^L\sum_{k,\ell,m}\partial_{\phi_k}\partial_{\phi_m}\phi_\ell e^{i\ell x\frac{2\pi}{L}}\left(e^{-iky\frac{2\pi}{L}} - e^{-ikx\frac{2\pi}{L}}\right)\left(e^{-imy\frac{2\pi}{L}} - e^{-imx\frac{2\pi}{L}}\right) \\ &\qquad\qquad \times K(x-y)P(\phi)\,\mathrm{d}y\,\mathrm{d}x, \\ &= \lim_{h\to 0}\frac{1}{L^2h}\int_0^L\sum_{k,\ell,m}\partial_{\phi_k}\partial_{\phi_m}\phi_\ell e^{i\ell x\frac{2\pi}{L}}\left(e^{-ik\frac{2\pi}{L}(x+vh)} - e^{-ik\frac{2\pi}{L}x}\right) \\ &\qquad\qquad \times\left(e^{-im\frac{2\pi}{L}(x+vh)} - e^{-im\frac{2\pi}{L}x}\right)P(\phi)\,\mathrm{d}x, \\ &= \lim_{h\to 0}\frac{1}{L^2h}\int_0^L\sum_{k,\ell,m}\partial_{\phi_k}\partial_{\phi_m}\phi_\ell e^{i\ell x\frac{2\pi}{L}}e^{-i(k+m)\frac{2\pi}{L}x}\left(-ikvh\tfrac{2\pi}{L} + \mathcal{O}(h^2)\right) \\ &\qquad\qquad \times\left(-imvh\tfrac{2\pi}{L} + \mathcal{O}(h^2)\right)P(\phi)\,\mathrm{d}x, \\ &= \lim_{h\to 0}\frac{h}{L^2}\left(\int_0^L\sum_{k,\ell,m}\partial_{\phi_k}\partial_{\phi_m}\phi_\ell e^{i\ell\frac{2\pi}{L}x}e^{-i(k+m)x}kmv^2\tfrac{4\pi^2}{L^2}P(\phi)\,\mathrm{d}x + \mathcal{O}(h)\right) = 0.\end{aligned}$$

Combining both integrals and going back to real space, our master equation becomes

$$\partial_t P(\phi) = \int \frac{\delta}{\delta\phi(x)} \left(v\frac{\partial}{\partial x}\phi(x)P(\phi) \right) \mathrm{d}x. \tag{SI.2.7}$$

Thus, particle movement will correspond to an advection term in the limit $N \to \infty$, while not contributing to any fluctuations.

SI.2.2 Locusts interactions

The interactions considered in a spatial model for the locust system are a subset of the general model described in Table SI.1 with the addition of the interaction $\mathcal{R} + \mathcal{L} \xrightarrow{r_6} 2\mathcal{S}$ which considers both moving locusts to stop as the result of a direct collision. The following chemical reactions represent the locust model interactions

$$\begin{array}{ccc}
\mathcal{R} \underset{r_2}{\overset{r_1}{\rightleftarrows}} \mathcal{S} & \mathcal{R}+\mathcal{S} \xrightarrow{r_3} 2\mathcal{S} & \mathcal{R}+\mathcal{S} \xrightarrow{r_4} 2\mathcal{R} \\
\mathcal{L} \underset{r_2}{\overset{r_1}{\rightleftarrows}} \mathcal{S} & \mathcal{L}+\mathcal{S} \xrightarrow{r_3} 2\mathcal{S} & \mathcal{L}+\mathcal{S} \xrightarrow{r_4} 2\mathcal{L} \\
\mathcal{R}+\mathcal{L} \xrightarrow{r_5} \mathcal{R}+\mathcal{S} & \mathcal{R}+\mathcal{L} \xrightarrow{r_5} \mathcal{S}+\mathcal{L} & \mathcal{R}+\mathcal{L} \xrightarrow{r_6} 2\mathcal{S},
\end{array}$$

where $\mathcal{R}$, $\mathcal{L}$ and $\mathcal{S}$ represent right-moving, left-moving and stopped locusts, respectively. This model relates to our general model in Section SI.1 by setting $\mathcal{R}$ and $\mathcal{L}$ to X and Y, and $\mathcal{S}$ to W with rates $a_{XY} = 0, b_{XY} = 0, c_{XY} = 0, a_{XW} = r_1, b_{XW} = r_3, c_{XW} = r_5, a_{WX} = r_2, b_{WX} = r_4, c_{OA} = 0$. The addition of r_6 does not affect the results significantly, playing a role similar to the parameter r_5. It has been added, however, for completeness of possible interactions.

While the spontaneous reactions $\mathcal{R}/\mathcal{L} \to \mathcal{S}$ and $\mathcal{S} \to \mathcal{R}/\mathcal{L}$ can happen regardless of the presence of neighbouring locusts, the two-body interactions require both interacting locusts to be within a radius R of each other.

In terms of creation/annihilation operators, the reaction $\mathcal{R} \xrightarrow{r_1} \mathcal{S}$ corresponds to a term $(\Delta_x^{s-}\Delta_x^{r+} - 1)r_1\phi^r(x)P(\phi)$ in our master equation, for which we ‘annihilate’ a particle of species r and ‘create’ another of species s at position x. Similarly, the reaction $\mathcal{R}+\mathcal{S} \to 2\mathcal{S}$ corresponds to the term

$$\int_0^L (\Delta_x^{s-}\Delta_x^{r+} - 1)r_3\phi^r(x)\phi^s(y)\mathbb{1}^R(x-y)P(\phi)\,\mathrm{d}y, \tag{SI.2.8}$$

where $\mathbb{1}^R(x-y)$ is 1 if $|x-y| \le R$ and 0 otherwise. Note that, while r_1 and r_2 are the same in both models, r_3 and the other rates will have to be adjusted in order to get the right limit

as $2R \to L$. In summary, our reactions can be written as,

$$
\begin{aligned}
Q_1(\phi, x) &= (\Delta_x^{s-}\Delta_x^{\ell+} - 1) r_1 \phi^\ell(x) + (\Delta_x^{s-}\Delta_x^{r+} - 1) r_1 \phi^r(x), \\
Q_2(\phi, x) &= (\Delta_x^{\ell-}\Delta_x^{s+} - 1) r_2 \phi^s(x) + (\Delta_x^{r-}\Delta_x^{s+} - 1) r_2 \phi^s(x),
\end{aligned}
$$

for the spontaneous reactions. The two-body interactions are then

$$
\begin{aligned}
Q_3(\phi, x) &= \int_0^L (\Delta_x^{s-}\Delta_x^{r+} - 1) r_3 \phi^r(x)\phi^s(y) \mathbb{1}^R(x-y)\,\mathrm{d}y \\
&\quad + \int_0^L (\Delta_x^{s-}\Delta_x^{\ell+} - 1) r_3 \phi^\ell(x)\phi^s(y) \mathbb{1}^R(x-y)\,\mathrm{d}y, \\
Q_4(\phi, x) &= \int_0^L (\Delta_x^{r-}\Delta_x^{s+} - 1) r_4 \phi^s(x)\phi^r(y) \mathbb{1}^R(x-y)\,\mathrm{d}y \\
&\quad + \int_0^L (\Delta_x^{\ell-}\Delta_x^{s+} - 1) r_4 \phi^s(x)\phi^\ell(y) \mathbb{1}^R(x-y)\,\mathrm{d}y, \\
Q_5(\phi, x) &= \int_0^L (\Delta_x^{s-}\Delta_x^{r+} - 1) r_5 \phi^r(x)\phi^\ell(y) \mathbb{1}^R(x-y)\,\mathrm{d}y \\
&\quad + \int_0^L (\Delta_x^{s-}\Delta_x^{\ell+} - 1) r_5 \phi^\ell(x)\phi^r(y) \mathbb{1}^R(x-y)\,\mathrm{d}y, \\
Q_6(\phi, x) &= \int_0^L (\Delta_x^{s-}\Delta_y^{s-}\Delta_x^{r+}\Delta_y^{\ell+} - 1) r_6 \phi^r(x)\phi^\ell(y) \mathbb{1}^R(x-y)\,\mathrm{d}y.
\end{aligned}
$$

In order to derive a functional Fokker-Planck equation we perform a Kramers-Moyal expansion of our operators, which gives

$$
\Delta_x^{g\pm} = 1 \pm \frac{1}{N}\frac{\delta}{\delta\phi^g(x)} + \frac{1}{2N^2}\frac{\delta^2}{\delta\phi^g(x)^2} + \mathcal{O}(N^{-3}), \qquad g \in \{r, \ell, s\}. \tag{SI.2.9}
$$

Up to second-order, we find

$$
\begin{aligned}
Q_1(\phi, x) = &\ \frac{1}{N}\left(\frac{\delta}{\delta\phi^\ell(x)} - \frac{\delta}{\delta\phi^s(x)}\right) r_1\phi^\ell(x) + \frac{1}{N}\left(\frac{\delta}{\delta\phi^r(x)} - \frac{\delta}{\delta\phi^s(x)}\right) r_1\phi^r(x) \\
&+ \frac{1}{2N^2}\left(\frac{\delta}{\delta\phi^\ell(x)} - \frac{\delta}{\delta\phi^s(x)}\right)^2 r_1\phi^\ell(x) + \frac{1}{2N^2}\left(\frac{\delta}{\delta\phi^r(x)} - \frac{\delta}{\delta\phi^s(x)}\right)^2 r_1\phi^r(x), \\
Q_2(\phi, x) = &\ \frac{1}{N}\left(\frac{\delta}{\delta\phi^s(x)} - \frac{\delta}{\delta\phi^r(x)}\right) r_2\phi^s(x) + \frac{1}{N}\left(\frac{\delta}{\delta\phi^s(x)} - \frac{\delta}{\delta\phi^\ell(x)}\right) r_2\phi^s(x) \\
&+ \frac{1}{2N^2}\left(\frac{\delta}{\delta\phi^s(x)} - \frac{\delta}{\delta\phi^r(x)}\right)^2 r_2\phi^s(x) + \frac{1}{2N^2}\left(\frac{\delta}{\delta\phi^s(x)} - \frac{\delta}{\delta\phi^\ell(x)}\right)^2 r_2\phi^s(x),
\end{aligned}
$$

$$
\begin{aligned}
Q_3(\phi,x) &= \frac{1}{N}\left(\frac{\delta}{\delta\phi^r(x)}-\frac{\delta}{\delta\phi^s(x)}\right)\int_0^L r_3\phi^r(x)\phi^s(y)\mathbb{1}^R(x-y)\,\mathrm{d}y \\
&+\frac{1}{2N^2}\left(\frac{\delta}{\delta\phi^r(x)}-\frac{\delta}{\delta\phi^s(x)}\right)^2\int_0^L r_3\phi^r(x)\phi^s(y)\mathbb{1}^R(x-y)\,\mathrm{d}y \\
&+\frac{1}{N}\left(\frac{\delta}{\delta\phi^\ell(x)}-\frac{\delta}{\delta\phi^s(x)}\right)\int_0^L r_3\phi^\ell(x)\phi^s(y)\mathbb{1}^R(x-y)\,\mathrm{d}y \\
&+\frac{1}{2N^2}\left(\frac{\delta}{\delta\phi^\ell(x)}-\frac{\delta}{\delta\phi^s(x)}\right)^2\int_0^L r_3\phi^\ell(x)\phi^s(y)\mathbb{1}^R(x-y)\,\mathrm{d}y, \\
Q_4(\phi,x) &= \frac{1}{N}\left(\frac{\delta}{\delta\phi^s(x)}-\frac{\delta}{\delta\phi^r(x)}\right)\int_0^L r_4\phi^s(x)\phi^r(y)\mathbb{1}^R(x-y)\,\mathrm{d}y \\
&+\frac{1}{2N^2}\left(\frac{\delta}{\delta\phi^s(x)}-\frac{\delta}{\delta\phi^r(x)}\right)^2\int_0^L r_4\phi^s(x)\phi^r(y)\mathbb{1}^R(x-y)\,\mathrm{d}y \\
&+\frac{1}{N}\left(\frac{\delta}{\delta\phi^s(x)}-\frac{\delta}{\delta\phi^\ell(x)}\right)\int_0^L r_4\phi^s(x)\phi^\ell(y)\mathbb{1}^R(x-y)\,\mathrm{d}y \\
&+\frac{1}{2N^2}\left(\frac{\delta}{\delta\phi^s(x)}-\frac{\delta}{\delta\phi^\ell(x)}\right)^2\int_0^L r_4\phi^s(x)\phi^\ell(y)\mathbb{1}^R(x-y)\,\mathrm{d}y, \\
Q_5(\phi,x) &= \frac{1}{N}\left(\frac{\delta}{\delta\phi^r(x)}-\frac{\delta}{\delta\phi^s(x)}\right)\int_0^L r_5\phi^r(x)\phi^\ell(y)\mathbb{1}^R(x-y)\,\mathrm{d}y \\
&+\frac{1}{2N^2}\left(\frac{\delta}{\delta\phi^r(x)}-\frac{\delta}{\delta\phi^s(x)}\right)^2\int_0^L r_5\phi^r(x)\phi^\ell(y)\mathbb{1}^R(x-y)\,\mathrm{d}y \\
&+\frac{1}{N}\left(\frac{\delta}{\delta\phi^\ell(x)}-\frac{\delta}{\delta\phi^s(x)}\right)\int_0^L r_5\phi^r(x)\phi^\ell(y)\mathbb{1}^R(x-y)\,\mathrm{d}y \\
&+\frac{1}{2N^2}\left(\frac{\delta}{\delta\phi^\ell(x)}-\frac{\delta}{\delta\phi^s(x)}\right)^2\int_0^L r_5\phi^r(x)\phi^\ell(y)\mathbb{1}^R(x-y)\,\mathrm{d}y.
\end{aligned}
$$

For $Q_6(\phi,x)$, the leading order term is

$$
\begin{aligned}
\int Q_6(\phi,x)P(\phi)\,\mathrm{d}x = \frac{1}{N}\int_0^L\int_{-\pi}^{\pi}&\left(\frac{\delta}{\delta\phi^r(x)}-\frac{\delta}{\delta\phi^s(x)}+\frac{\delta}{\delta\phi^\ell(y)}-\frac{\delta}{\delta\phi^s(y)}\right) \\
&\times r_6\phi^r(x)\phi^\ell(y)\mathbb{1}^R(x-y)P(\phi)\,\mathrm{d}y\,\mathrm{d}x.
\end{aligned}
$$

Expanding terms,

$$
\begin{aligned}
\int Q_6(\phi,x)P(\phi)\,\mathrm{d}x &= \frac{1}{N}\int_0^L\frac{\delta}{\delta\phi^r(x)}\left\{r_6\phi^r(x)\int_0^L\phi^\ell(y)\mathbb{1}^R(x-y)P(\phi)\,\mathrm{d}y\right\}\mathrm{d}x \\
&-\frac{1}{N}\int_0^L\frac{\delta}{\delta\phi^s(x)}\left\{r_6\phi^r(x)\int_0^L\phi^\ell(y)\mathbb{1}^R(x-y)P(\phi)\,\mathrm{d}y\right\}\mathrm{d}x \\
&+\frac{1}{N}\int_0^L\int_0^L\frac{\delta}{\delta\phi^\ell(y)}r_6\phi^r(x)\phi^\ell(y)\mathbb{1}^R(x-y)P(\phi)\,\mathrm{d}y\,\mathrm{d}x \\
&-\frac{1}{N}\int_0^L\int_0^L\frac{\delta}{\delta\phi^s(y)}r_6\phi^r(x)\phi^\ell(y)\mathbb{1}^R(x-y)P(\phi)\,\mathrm{d}y\,\mathrm{d}x.
\end{aligned}
$$

In the last two integrals, we can simply make the change $x\leftrightarrow y$ and change the order of

integration to obtain

$$\int Q_6(\phi,x)P(\phi)\,\mathrm{d}x = \frac{1}{N}\int_0^L \left(\frac{\delta}{\delta\phi^r(x)} - \frac{\delta}{\delta\phi^s(x)}\right)\left\{r_6\phi^r(x)\int_0^L \phi^\ell(y)\mathbb{1}^R(x-y)P(\phi)\,\mathrm{d}y\right\}\mathrm{d}x$$
$$+\frac{1}{N}\int_0^L \left(\frac{\delta}{\delta\phi^\ell(x)} - \frac{\delta}{\delta\phi^s(x)}\right)\left\{r_6\phi^\ell(x)\int_0^L \phi^r(y)\mathbb{1}^R(x-y)P(\phi)\,\mathrm{d}y\right\}\mathrm{d}x.$$

Multiplying the functional master equation (SI.2.2) by $\phi^g(x), g \in \{r,\ell,s\}$, integrating over $\delta\phi^\ell\delta\phi^r\delta\phi^s$, and using a mean-field approximation, we get equations for $\partial_t\phi^g$. In the limit $N\to\infty$, the equations become

$$\partial_t\phi^\ell = -v\partial_x\phi^\ell + r_2\phi^s - r_1\phi^\ell + \phi^s\int r_4\phi^\ell(y)\mathbb{1}^R(x-y)\,\mathrm{d}y \tag{SI.2.10a}$$
$$-\phi^\ell\left(\int r_3\phi^s(y)\mathbb{1}^R(x-y)\,\mathrm{d}y + \int (r_5+r_6)\phi^r(y)\mathbb{1}^R(x-y)\,\mathrm{d}y\right),$$
$$\partial_t\phi^r = v\partial_x\phi^r + r_2\phi^s - r_1\phi^r + \phi^s\int r_4\phi^r(y)\mathbb{1}^R(x-y)\,\mathrm{d}y \tag{SI.2.10b}$$
$$-\phi^r\left(\int r_3\phi^s(y)\mathbb{1}^R(x-y)\,\mathrm{d}y + \int (r_5+r_6)\phi^\ell(y)\mathbb{1}^R(x-y)\,\mathrm{d}y\right),$$
$$\partial_t\phi^s = r_1(\phi^\ell+\phi^r) - 2r_2\phi^s - \phi^s\left(\int r_4(\phi^\ell+\phi^r)(y)\mathbb{1}^R(x-y)\,\mathrm{d}y\right) \tag{SI.2.10c}$$
$$+\phi^\ell\left(\int (r_5+r_6)\phi^r(y)\mathbb{1}^R(x-y)\,\mathrm{d}y\right) + \phi^r\left(\int (r_5+r_6)\phi^\ell(y)\mathbb{1}^R(x-y)\,\mathrm{d}y\right)$$
$$+(\phi^\ell+\phi^r)\left(\int r_3\phi^s(y)\mathbb{1}^R(x-y)\,\mathrm{d}y\right).$$

We will now analyse this set of equations to understand under which circumstances the spatial model can be accurately approximated by its well-mixed limit to obtain analytical predictions of the stationary probability distribution and mean switching times as demonstrated in Section SI.1.

SI.2.3 Well-mixed limits

The general model introduced in Section SI.1 considers the dynamics of well-mixed populations. While we have argued that the locust system is an example of a collective decision-making process, it remains to show that its dynamics can be approximated by the description of Section SI.1. This is the focus of the remainder of Section SI.2.

The most obvious limit that takes us from the spatial model to the well-mixed model is to allow $2R \to L$, where L is the size of the annular arena. In this case,

$$\int f(y)\mathbb{1}^R(x-y)\,\mathrm{d}y = \int f(y)\,\mathrm{d}y. \tag{SI.2.11}$$

Hence,

$$\begin{aligned}
\partial_t \phi^\ell &= -v\partial_x \phi^\ell + r_2\phi^s - r_1\phi^\ell - \phi^\ell\{r_3 s(t) + (r_5+r_6)r(t)\} + \phi^s r_4 \ell(t),\\
\partial_t \phi^r &= v\partial_x \phi^r + r_2\phi^s - r_1\phi^r - \phi^r\{r_3 s(t) + (r_5+r_6)\ell(t)\} + \phi^s r_4 r(t),\\
\partial_t \phi^s &= r_1(\phi^\ell + \phi^r) - 2r_2\phi^s - \phi^s\{r_4[\ell(t)+r(t)]\} + (\phi^\ell+\phi^r) r_3 s(t)\\
&\quad + 2(r_5+r_6)\{\phi^\ell r(t) + \phi^r \ell(t)\},
\end{aligned}$$

where $g(t) = \int_0^L \phi^g(x,t)\,\mathrm{d}x$ for $g \in \{r, \ell, s\}$. Integrating the above equations over the whole arena, and using the periodic boundary conditions, $\phi^g(L,t) = \phi^g(0,t)$, gives

$$\begin{aligned}
\frac{\mathrm{d}\ell}{\mathrm{d}t} &= r_2 s - r_1\ell + (r_4 - r_3)\ell s - (r_5+r_6)\ell r,\\
\frac{\mathrm{d}r}{\mathrm{d}t} &= r_2 s - r_1 r + (r_4 - r_3) r s - (r_5+r_6) r\ell,\\
\frac{\mathrm{d}s}{\mathrm{d}t} &= r_1(\ell + r) - 2r_2 s - (r_4 - r_3)s(\ell + r) + 2(r_5+r_6)\ell r,
\end{aligned}$$

which are the same equations obtained for the well-mixed model in Section SI.3.

In this first case, the well-mixed limit means that every particle interacts with every other particle in the system since the interaction radius encompasses the whole arena. Nonetheless, well-mixedness can also arise in the limit where the particles encounter each other several times before interacting, so the probability of interacting with any of them is approximately uniform. This corresponds to the limit where the speed of propagation, v, is large, while the rates of interaction r_k are small, leading to a separation of timescales: the fast timescale of the movement of the particles around the arena and the slow timescale of the interactions.

To make this formal, let us rescale space, $x = x'L$, and time, $t' = \bar{r}t$, for $\bar{r} = \max_i\{r_i\}$. These definitions mean x' and t' are dimensionless. We define the dimensionless parameter $\varepsilon = \frac{\bar{r}L}{v}$. Let $\alpha_i = \frac{r_i}{\bar{r}}$ be dimensionless rescaled rates. Consider the following expansion for our variables $\phi^g(x,t) = \sum_{i=0}^{\infty} \varepsilon^i \phi_i^g(x,t)$, i.e., we expand our variables in powers or ε.

Substituting this in Eq. (SI.2.10) gives

$$\varepsilon \frac{\partial}{\partial t'}\left\{\phi_0^\ell + \varepsilon\phi_1^\ell + \dots\right\} = -\partial_{x'}(\phi_0^\ell + \varepsilon\phi_1^\ell + \dots) + \varepsilon\alpha_2(\phi_0^s + \varepsilon\phi_1^s) + \dots \tag{SI.2.13}$$

We now solve the equations in orders of ε^i. At order ε^0,

$$0 = -\partial_{x'}\phi_0^\ell, \qquad\qquad 0 = \partial_{x'}\phi_0^r, \tag{SI.2.14}$$

i.e., $\phi_0^\ell(x,t) = \ell_0(t)$ and $\phi_0^r(x,t) = r_0(t)$. Although in zeroth order we do not get an equation

for ϕ_0^s, we can use the conservation rule

$$\int \phi^\ell(x,t) + \phi^r(x,t) + \phi^s(x,t)\,\mathrm{d}x = 1. \tag{SI.2.15}$$

This means that $\phi_i^\ell + \phi_i^s + \phi_i^r$ is conserved for every i. Therefore, $\phi_0^s(x,t) = s_0(t)$. Intuitively, the particles move so fast that it seems, to the other particles, as if there is a continuous homogeneous flow of particles, even of stopped ones, since in the frame of reference of the moving particles, the stopped particles are the ones moving.

Now, at order ε,

$$\begin{aligned}
\partial_t \phi_0^\ell &= -\partial_x \phi_1^\ell + \alpha_2 \phi_0^s - \alpha_1 \phi_0^\ell + \phi_0^s \int \alpha_4 \phi_0^\ell(y,t) \mathbb{1}^R(x-y)\,\mathrm{d}y \\
&\quad - \phi_0^\ell \left(\int \alpha_3 \phi_0^s(y,t) \mathbb{1}^R(x-y)\,\mathrm{d}y + \int (\alpha_5+\alpha_6)\phi_0^r(y,t)\mathbb{1}^R(x-y)\,\mathrm{d}y \right) \\
&= -\partial_x \phi_1^\ell + \alpha_2 \phi_0^s - \alpha_1 \phi_0^\ell + \alpha_4 \phi_0^s \frac{2R}{L} \ell_0(t) - \alpha_3 \phi_0^\ell \frac{2R}{L} s_0(t) - (\alpha_5+\alpha_6)\phi_0^\ell \frac{2R}{L} r_0(t).
\end{aligned}$$

To eliminate the dependency on ϕ_1^g we integrate over the whole arena,

$$\frac{\mathrm{d}\ell_0}{\mathrm{d}t} = \alpha_2 s_0(t) - \alpha_1 \ell_0(t) + \frac{2R}{L}\left\{ \alpha_4 s_0(t)\ell_0(t) - \alpha_3 \ell_0(t) s_0(t) - (\alpha_5+\alpha_6)\ell_0(t) r_0(t) \right\}.$$

Thus, as long as we scale our rates by $\frac{L}{2R}$, we obtain the same dynamics as in the well-mixed model for the zeroth-order terms. A similar procedure gives the well-mixed equations for $r_0(t)$ and $s_0(t)$

$$\begin{aligned}
\frac{\mathrm{d}r_0}{\mathrm{d}t} &= \alpha_2 s_0(t) - \alpha_1 r_0(t) + \frac{2R}{L}\left\{ \alpha_4 s_0(t) r_0(t) - \alpha_3 r_0(t) s_0(t) - (\alpha_5+\alpha_6)\ell_0(t) r_0(t) \right\}, \\
\frac{\mathrm{d}s_0}{\mathrm{d}t} &= -2\alpha_2 s_0(t) + \alpha_1 [r_0(t) + \ell_0(t)] - \frac{2R}{L}\left\{ (\alpha_4 - \alpha_3) s_0(t) [r_0(t) + \ell_0(t)] \right\} \\
&\quad + \frac{4R}{L}\left\{ (\alpha_5+\alpha_6)\ell_0(t) r_0(t) \right\}.
\end{aligned}$$

Hence, we have shown that the well-mixed model (see Section SI.3) is the first-order approximation when $\varepsilon \to 0$ (with appropriately rescaled parameters), and exact when $2R \to L$. Higher-order corrections can be obtained similarly.

We note that, while left and right movers interact twice as often as movers and stopped locusts – left and right movers meet twice per loop – the doubled number of encounters is balanced out by the meetings being half as long, as the effective speed between left and right is twice as large. Hence, the effective time that particles interact with each other is the same, regardless of the stance of the particles.

Now we have shown the two limits where the spatial model becomes well-mixed, we study the spatial model outside them to check if there are any significant spatial dynamics overlooked

by our non-spatial modelling approach.

SI.2.4 Homogeneous solutions

As Eq. (SI.2.10) contains several convolutions in physical space, it is easier to work in Fourier space. In this case, taking $v\frac{2\pi}{L} \mapsto v$, our equations become

$$\begin{aligned}\frac{\mathrm{d}\phi_k^\ell}{\mathrm{d}t} =& -ivk\phi_k^\ell + r_2\phi_k^s - r_1\phi_k^\ell - r_3 L\sum_m \mathbb{1}_m^R \phi_m^s \phi_{k-m}^\ell + r_4 L \sum_m \mathbb{1}_m^R \phi_m^\ell \phi_{k-m}^a \\ &- (r_5+r_6)L\sum_m \mathbb{1}_m^R \phi_m^r \phi_{k-m}^\ell, \qquad \text{(SI.2.16a)} \\ \frac{\mathrm{d}\phi_k^r}{\mathrm{d}t} =& -ivk\phi_k^r + r_2\phi_k^s - r_1\phi_k^r - r_3 L\sum_m \mathbb{1}_m^R \phi_m^s \phi_{k-m}^\ell + r_4 L \sum_m \mathbb{1}_m^R \phi_m^\ell \phi_{k-m}^a \\ &- (r_5+r_6)L\sum_m \mathbb{1}_m^R \phi_m^\ell \phi_{k-m}^r. \qquad \text{(SI.2.16b)}\end{aligned}$$

And a corresponding equation for ϕ_k^s. We now look for homogeneous solutions $\phi_k^\ell = \frac{\varphi^\ell}{L}\delta_{0,k}$, $\phi_k^r = \frac{\varphi^r}{L}\delta_{0,k}$, $\phi_k^s = \frac{(1-\varphi^\ell-\varphi^r)}{L}\delta_{0,k}$. For $k \neq 0$, the equations are trivially zero. For $k = 0$,

$$\begin{aligned}0 &= r_2(1-\varphi^\ell-\varphi^r) - r_1\varphi^\ell + (r_4-r_3)L\frac{2R}{L}\frac{\varphi^\ell}{L}(1-\varphi^\ell-\varphi^r) - (r_5+r_6)L\frac{2R}{L}\frac{\varphi^r}{L}\varphi^\ell, \\ 0 &= r_2(1-\varphi^\ell-\varphi^r) - r_1\varphi^r + (r_4-r_3)L\frac{2R}{L}\frac{\varphi^r}{L}(1-\varphi^\ell-\varphi^r) - (r_5+r_6)L\frac{2R}{L}\frac{\varphi^\ell}{L}\varphi^r,\end{aligned}$$

where $\mathbb{1}_0^R = \frac{2R}{L}$. Subtracting the first equation from the second,

$$r_1(\varphi^\ell-\varphi^r) - (r_4-r_3)\frac{2R}{L}(1-\varphi^\ell-\varphi^r)(\varphi^\ell-\varphi^r) = 0. \qquad \text{(SI.2.17)}$$

Hence, we get the solutions $\varphi^\ell = \varphi^r = \varphi$, which we can substitute in the earlier equations to find φ, or $\varphi^{s*} = (1-\varphi^\ell-\varphi^r) = \frac{r_1 L}{2R(r_4-r_3)}$. In the latter case,

$$\varphi_\pm^\ell = \frac{1}{2}\left((1-\varphi^{s*}) \pm \sqrt{(1-\varphi^{s*})^2 - 4\frac{r_1}{r_4-r_3}\frac{r_2}{r_5+r_6}\left(\frac{L}{2R}\right)^2}\right). \qquad \text{(SI.2.18)}$$

This result agrees with Eq. (SI.1.9) in the limit $2R \to L$, in which $\varphi^{s*} = \alpha$ and $\kappa = \frac{r_2}{r_5+r_6}$ following the mapping between parameters. Note that, although φ^ℓ might have real solutions in the well-mixed system ($2R \to L$), for sufficiently small R, real solutions cease to exist and the only steady state of the system is a disordered one $\varphi^\ell = \varphi^r$. That makes sense, as in the limit $R \to 0$ the particles cease to interact with each other and will only spontaneously switch between moving and stopping stances. We now check the linear stability of the steady states of the system to determine whether patterns can form.

Stability analysis

In this section, we do a linear stability analysis of the homogeneous steady states of the system. We show that the aligned system states are stable when they exist inside the simplex, while the disordered system state is stable when it does not. We also show that no spatial patterns can form, supporting our choice to study the simpler well-mixed model.

First, we propose a linear fluctuation ansatz [Van Kampen, 2007]:

$$\phi_k^\ell = \frac{\varphi^\ell}{L}\delta_{k,0} + \frac{\xi_k^\ell}{\sqrt{N}}, \quad \phi_k^r = \frac{\varphi^r}{L}\delta_{k,0} + \frac{\xi_k^r}{\sqrt{N}}, \quad \phi_k^s = \frac{(1-\varphi^\ell-\varphi^r)}{L}\delta_{k,0} - \frac{\xi_k^\ell}{\sqrt{N}} - \frac{\xi_k^r}{\sqrt{N}}.$$

Substituting these into the Fourier transformed equations (SI.2.16) with $r'' = r_5 + r_6$:

$$\begin{aligned}
\frac{\mathrm{d}\xi_0^\ell}{\mathrm{d}t} &= r_2(-\xi_0^\ell - \xi_0^r) - r_1\xi_0^\ell + r_4\left(\mathbb{1}_0^R\varphi^\ell(-\xi_0^\ell - \xi_0^r) + \mathbb{1}_0^R(1-\varphi^\ell-\varphi^r)\xi_0^\ell\right) \\
&\quad - r_3\left(\mathbb{1}_0^R(1-\varphi^\ell-\varphi^r)\xi_0^\ell + \mathbb{1}_0^R\varphi^\ell(-\xi_0^\ell - \xi_0^r)\right) - r''\left(\mathbb{1}_0^R\varphi^\ell\xi_0^r + \mathbb{1}_0^R\varphi^r\xi_0^\ell\right) + \mathcal{O}(\xi^2), \\
\frac{\mathrm{d}\xi_0^r}{\mathrm{d}t} &= r_2(-\xi_0^\ell - \xi_0^r) - r_1\xi_0^r + r_4\left(\mathbb{1}_0^R\varphi^r(-\xi_0^\ell - \xi_0^r) + \mathbb{1}_0^R(1-\varphi^\ell-\varphi^r)\xi_0^r\right) \\
&\quad - r_3\left(\mathbb{1}_0^R(1-\varphi^\ell-\varphi^r)\xi_0^r + \mathbb{1}_0^R\varphi^r(-\xi_0^\ell - \xi_0^r)\right) - r''\left(\mathbb{1}_0^R\varphi^r\xi_0^\ell + \mathbb{1}_0^R\varphi^\ell\xi_0^r\right) + \mathcal{O}(\xi^2). \\
\frac{\mathrm{d}\xi_k^\ell}{\mathrm{d}t} &= -ivk\xi_k^\ell + r_2(-\xi_k^\ell - \xi_k^r) - r_1\xi_k^\ell + r_4\left(\mathbb{1}_0^R\varphi^\ell(-\xi_k^\ell - \xi_k^r) + \mathbb{1}_k^R(1-\varphi^\ell-\varphi^r)\xi_k^\ell\right) \\
&\quad - r_3\left(\mathbb{1}_0^R(1-\varphi^\ell-\varphi^r)\xi_k^\ell + \mathbb{1}_k^R\varphi^\ell(-\xi_k^\ell - \xi_k^r)\right) - r''\left(\mathbb{1}_0^R\varphi^r\xi_k^\ell + \mathbb{1}_k^R\varphi^\ell\xi_k^r\right) + \mathcal{O}(\xi^2), \\
\frac{\mathrm{d}\xi_k^r}{\mathrm{d}t} &= ivk\xi_k^r + r_2(-\xi_k^\ell - \xi_k^r) - r_1\xi_k^r + r_4\left(\mathbb{1}_0^R\varphi^r(-\xi_k^\ell - \xi_k^r) + \mathbb{1}_k^R(1-\varphi^\ell-\varphi^r)\xi_k^r\right) \\
&\quad - r_3\left(\mathbb{1}_0^R(1-\varphi^\ell-\varphi^r)\xi_k^r + \mathbb{1}_k^R\varphi^r(-\xi_k^\ell - \xi_k^r)\right) - r''\left(\mathbb{1}_0^R\varphi^\ell\xi_k^r + \mathbb{1}_k^R\varphi^r\xi_k^\ell\right) + \mathcal{O}(\xi^2).
\end{aligned}$$

Hence, for each k we have a 2 variable system to check stability, i.e., for $k = 0$ we get the matrix

$$M^{(0)} = \begin{pmatrix} -r_2 - r_1 + \rho'\left(\varphi^s - \varphi^\ell\right) - \rho''\varphi^r & -r_2 - \varphi^\ell(\rho' + \rho'') \\ -r_2 - \varphi^r(\rho' + \rho'') & -r_2 - r_1 + \rho'\left(\varphi^s - \varphi^r\right) - \rho''\varphi^\ell \end{pmatrix},$$

where $\rho = \frac{2R}{L}$, $\rho' = (r_4 - r_3)\frac{2R}{L}, \varphi^s = 1 - \varphi^\ell - \varphi^r$, and $\rho'' = (r_5 + r_6)\frac{2R}{L}$.

For $k \neq 0$, we can write $M^{(k)} = M^{(0)} + E^{(k)}$, where

$$E^{(k)} = \begin{pmatrix} \rho r_4(1-\varphi^\ell-\varphi^r) + \rho r_3\varphi^\ell & \rho(r_3 - r'')\varphi^\ell \\ \rho(r_3 - r'')\varphi^r & \rho r_4(1-\varphi^\ell-\varphi^r) + \rho r_3\varphi^r \end{pmatrix} f_k + \begin{pmatrix} -ivk & 0 \\ 0 & ivk \end{pmatrix},$$

with

$$f_k = \frac{1}{\rho\pi k}\sin(k\pi\rho) - 1. \qquad \text{(SI.2.19)}$$

Note that $-2 < f_k \leq 0, \forall k \in \mathbb{R}$ with $f_k = 0 \iff k = 0$.

Disordered system state

For the solution $\varphi^\ell = \varphi^r = \varphi$, $M_{12}^{(k)} = M_{21}^{(k)}$, $\mathrm{Re}\Big\{M_{11}^{(k)}\Big\} = \mathrm{Re}\Big\{M_{22}^{(k)}\Big\} = M_R^{(k)}$,

$$\begin{aligned}
\mathrm{Tr}\, M^{(k)} &= M_{11}^{(k)} + M_{22}^{(k)} = 2M_R^{(k)}, \\
\det M^{(k)} &= M_R^2 + v^2k^2 - M_{12}^2, \\
\Delta &= 4M_R^2 - 4M_R^2 - 4v^2k^2 + 4M_{12}^2.
\end{aligned}$$

In particular, for $k = 0$, we get $\Delta = 4M_{12}^2$, hence, $\lambda_\pm^{\text{homo}} = M_R^{(0)} \pm M_{12}^{(0)}$, i.e, $\lambda_- = -r_1 + \rho'(1-2\varphi)$ and $\lambda_+ = 2M_{12}^{(0)} - r_1 + \rho'(1-2\varphi) = 2M_{12}^{(0)} + \lambda_-$, where $M_{12}^{(0)} = -r_2 - \varphi(\rho' + \rho'') < 0$. Consequently, λ_+ is negative whenever λ_- is negative. Due to the curvature of the manifold, the number of stopped locusts is larger at the disordered system state than at the aligned system states, if they exist. Consequently, $1 - 2\varphi \geq 1 - \varphi^r - \varphi^\ell = \frac{r_1}{\rho'}$ and $\lambda_- > 0$ if the aligned system state exists, otherwise $1 - 2\varphi < \frac{r_1}{\rho'}$, and $\lambda_- < 0$. Thus, the $k = 0$ mode of perturbations of the disordered solution is unstable if the aligned system state exists and stable if the disordered system state is the only physical fixed point.

Because $M_{12}^{(k)}$ is bounded, for large enough k, $\Delta(k)$ eventually becomes negative, and we get complex eigenvalues, meaning that our solutions are periodic (in time), and propagate with speed distinct to the locusts. It remains to see if the real part is negative or positive. Because $M_R^{(k)}$ has terms with both positive and negative signs, both cases are possible depending on parameter values. Note that, if the mode $k = 0$ of the disordered system state is stable, then $\lambda_- < 0 \Rightarrow M_R^{(0)} + |M_{12}^{(0)}| < 0 \Rightarrow M_R^{(0)} < 0$. Since $M_R^{(k)} \leq M_R^{(0)}$, all modes with complex eigenvalues are stable. It remains to show that the modes with real eigenvalues are also stable.

For that, note that

$$M_{12}^{(k)} = M_{21}^{(k)} = -r_2 - \varphi\left(r_4\rho - r_3\tfrac{1}{\pi k}\sin(k\pi\rho)\right) - r''\varphi\left(\tfrac{1}{\pi k}\sin(k\pi\rho)\right) < 0.$$

Furthermore,

$$M_R^{(k)} \leq M_{12}^{(k)} - r_1 + r_4\left(\tfrac{(1-2\varphi)}{\pi k}\sin(k\pi\rho)\right) - r_3\rho(1-2\varphi).$$

Hence,

$$\begin{aligned}
\lambda_\pm(k) &= M_R^{(k)} \pm \sqrt{M_{12}^2 - v^2k^2}, \\
&< M_R^{(k)} + |M_{12}^{(k)}|, \\
&\leq M_{12}^{(k)} - M_{12}^{(k)} - r_1 + (1-2\varphi)\left(\tfrac{r_4}{\pi k}\sin(k\pi\rho) - r_3\rho\right), \\
&\leq -r_1 + (1-2\varphi)\rho' < 0.
\end{aligned}$$

Since we showed earlier that $(1-2\varphi) < \frac{r_1}{\rho'}$ if the disordered system state is stable. Consequently, if the $k=0$ mode of the disordered system state is stable, all its modes are stable. Additionally, in the limit $k \to \infty$, $M_R = -r_2 - r_1 - \rho r_4 \varphi^\ell - \rho r_3(1-\varphi^\ell - \varphi^r) - \rho'' \varphi^r < 0$. Hence, the real part of the eigenvalues is always negative.

Note that we assume $r' = r_4 - r_3 > 0$ throughout, which is the necessary (but not sufficient) condition for the existence of the aligned steady state of the system inside the simplex. This is the parameter region of interest to observe the collective motion of locusts.

In summary, if $r' > 0$ and the $k=0$ mode of the disordered system state is stable, then all other modes are stable. If the $k=0$ mode of the disordered solution is unstable, the $k=0$ mode of the aligned system state becomes stable. In what follows, we check the stability of the higher modes of perturbations of the aligned system state.

Aligned system state

Our main interest is to know what happens to the aligned steady states of the system, hence, for $1-\varphi^\ell-\varphi^r = \frac{r_1}{\rho'}$, $\rho' > 0$,

$$\begin{aligned}
\mathrm{Tr}\Big\{M^{(0)}\Big\} &= -2r_2 - (\varphi^\ell+\varphi^r)(\rho'+\rho'') < 0, \\
\det\Big\{M^{(0)}\Big\} &= \rho'\rho''(\varphi^\ell-\varphi^r)^2 > 0, \\
\lambda_\pm^{(0)} &= -r_2 - \tfrac{1}{2}(\varphi^\ell+\varphi^r)(\rho'+\rho'') \\
&\quad \pm \tfrac{1}{2}\sqrt{\left[-2r_2-(\varphi^\ell+\varphi^r)(\rho'+\rho'')\right]^2 - 4\rho'\rho''(\varphi^\ell-\varphi^r)^2}.
\end{aligned}$$

Thus, the $k=0$ mode of the aligned steady state of the system (when it exists) is always stable. It remains to show that the higher order modes are stable if $k=0$ is stable. For the higher order modes of the aligned system state, the matrix $E^{(k)}$ becomes

$$E^{(k)} = \begin{pmatrix} \frac{r_1 r_4}{r'} + \rho r_3 \varphi^\ell & \rho(r_3 - r'')\varphi^\ell \\ \rho(r_3-r'')\varphi^r & \frac{r_1 r_4}{r'} + \rho r_3\varphi^r \end{pmatrix} f_k + \begin{pmatrix} -ivk & 0 \\ 0 & ivk \end{pmatrix}.$$

Hence,

$$\mathrm{Tr}\Big\{M^{(k)}\Big\} = \mathrm{Tr}\Big\{M^{(0)}\Big\} + \Big(2\tfrac{r_1 r_4}{r'} + \rho r_3(\varphi^\ell+\varphi^r)\Big) f_k < \mathrm{Tr}\Big\{M^{(0)}\Big\}. \tag{SI.2.20}$$

And,

$$\begin{aligned}
\det\Big\{M^{(k)}\Big\} &= \det\Big\{M^{(0)} + E^{(k)}\Big\} \\
&= (M_{11}^{(0)} + E_{11}^{(k)})(M_{22}^{(0)} + E_{22}^{(k)}) - (M_{12}^{(0)} + E_{12}^{(k)})(M_{21}^{(0)} + E_{21}^{(k)}), \\
&= \det\Big\{M^{(0)}\Big\} + \det\Big\{E^{(k)}\Big\} + M_{11}^{(0)}E_{22}^{(k)} + M_{22}^{(0)}E_{11}^{(k)} \\
&\quad - M_{12}^{(0)}E_{21}^{(k)} - M_{21}^{(0)}E_{12}^{(k)}.
\end{aligned} \tag{SI.2.21}$$

While $\mathrm{Tr}\Big\{M^{(k)}\Big\}$ is real, $\det\Big\{M^{(k)}\Big\}$ is complex, with

$$\begin{aligned}
\mathrm{Im}\Big\{\det\Big(M^{(k)}\Big)\Big\} &= \mathrm{Im}\Big\{\det\Big(E^{(k)}\Big)\Big\} + vk\left(M_{11}^{(0)} - M_{22}^{(0)}\right), \\
&= vk(\rho r_3 f_k - \rho' + \rho'')(\varphi^\ell - \varphi^r),
\end{aligned}$$

so that $\mathrm{Im}\Big\{\det\Big(M^{(k)}\Big)\Big\}$ is zero for the disordered system state, but not for the aligned system state. Hence, the eigenvalues of $M^{(k)}$ are not complex conjugates, in general. On the other hand, the real part of the determinant is

$$\begin{aligned}
&\mathrm{Re}\Big\{\det\Big(M^{(k)}\Big)\Big\} \\
&= \det\Big(M^{(0)}\Big) + \mathrm{Re}\Big\{\det\Big(E^{(k)}\Big)\Big\} + \mathrm{Re}\Big\{M_{11}^{(0)}E_{22}^{(k)} + M_{22}^{(0)}E_{11}^{(k)}\Big\} - M_{12}^{(0)}E_{21}^{(k)} - M_{21}^{(0)}E_{12}^{(k)}, \\
&= \det\Big(M^{(0)}\Big) + v^2k^2 + \left(\tfrac{r_1 r_4}{r'} + \rho r_3\varphi^\ell\right)\left(\tfrac{r_1 r_4}{r'} + \rho r_3\varphi^r\right) f_k^2 - \rho^2(r_3 - r'')^2\varphi^\ell\varphi^r f_k^2 \\
&\quad - (r_2 + \varphi^\ell\rho' + \rho''\varphi^r)(\tfrac{r_1 r_4}{r'} + \rho r_3\varphi^r) f_k - (r_2 + \varphi^r\rho' + \rho''\varphi^\ell)(\tfrac{r_1 r_4}{r'} + \rho r_3\varphi^\ell) f_k \\
&\quad + (r_2 + \varphi^\ell(\rho' + \rho''))\rho(r_3 - r'')\varphi^r f_k + (r_2 + \varphi^r(\rho' + \rho''))\rho(r_3 - r'')\varphi^\ell f_k, \\
&= \det\Big(M^{(0)}\Big) + v^2k^2 + \left[(\tfrac{r_1 r_4}{r'})^2 + \tfrac{r_1 r_4}{r'}\rho r_3(\varphi^\ell + \varphi^r) + 2\rho''\rho r_3\varphi^\ell\varphi^r\right] f_k^2 \\
&\quad - \tfrac{r_1 r_4}{r'}\left(2r_2 + (\rho' + \rho'')(\varphi^\ell + \varphi^r)\right) f_k - \rho''\rho r_3(\varphi^\ell - \varphi^r)^2 f_k \\
&\quad - \rho''[r_2(\varphi^\ell + \varphi^r) + 2\rho'\varphi^\ell\varphi^r + (2 + f_k)\rho''\varphi^\ell\varphi^r] f_k.
\end{aligned}$$

Since $2 < f_k \leq 0$, with $f_k = 0 \iff k = 0$, it follows that $\mathrm{Re}\Big\{\det\Big(M^{(k)}\Big)\Big\} \geq \det\Big(M^{(0)}\Big)$ with equality if and only if $k = 0$. Because $\det\Big\{M^{(k)}\Big\}$ is complex, this is not enough to guarantee stability, and we need to go a bit further.

Let $\lambda_1 = a_1 + ib_1$ and $\lambda_2 = a_2 + ib_2$ be the eigenvalues of $M^{(k)}$. Since $\mathrm{Tr}\Big\{M^{(k)}\Big\}$ is real,

$$\mathrm{Tr}\Big\{M^{(k)}\Big\} = \lambda_1 + \lambda_2 = (a_1 + a_2) + i(b_1 + b_2) \Rightarrow b_1 = -b_2 = b, \tag{SI.2.22}$$

$$\det\Big\{M^{(k)}\Big\} = \lambda_1 \cdot \lambda_2 = (a_1 a_2 + b^2) + ib(a_2 - a_1) \Rightarrow b \neq 0, \tag{SI.2.23}$$

for some $b \in \mathbb{R}$. Note that, $a_1 + a_2 = \mathrm{Tr}\Big\{M^{(k)}\Big\} \leq \mathrm{Tr}\Big\{M^{(0)}\Big\} < 0$, $(a_1 a_2 + b^2) =$

$\mathrm{Re}\Big\{\det\Big(M^{(k)}\Big)\Big\} \geq \det\Big(M^{(0)}\Big) > 0$ and $b(a_2 - a_1) = \mathrm{Im}\Big\{\det\Big(M^{(k)}\Big)\Big\}$. Hence, $a_1 a_2$ solves

$$\Big(a_1 a_2 - \mathrm{Re}\Big\{\det\Big(M^{(k)}\Big)\Big\}\Big)\left(4a_1 a_2 - \mathrm{Tr}\Big\{M^{(k)}\Big\}^2\right) = \mathrm{Im}\Big\{\det\Big(M^{(k)}\Big)\Big\}^2. \tag{SI.2.24}$$

Because $a_1 + a_2 < 0$, the higher order modes are stable if $a_1 a_2 > 0$. Considering k as a continuous variable, $\lambda_1(k)$, $\lambda_2(k)$ and $M^{(k)}$ are continuous functions of $k \in \mathbb{R}$. Hence, $\mathrm{Re}\{\lambda_1(k)\} = a_1(k)$ and $\mathrm{Re}\{\lambda_2(k)\} = a_2(k)$ are also continuous functions of k. Thus, a linearly unstable Fourier mode $k = k_c$ exists with either $a_1(k_c) > 0$ or $a_2(k_c) > 0$, only if there exists $k^* \in (0, k_c)$ such that $a_1(k^*) \cdot a_2(k^*) = 0$. Note that, while k_c is an integer, k^* does not need to be.

From Eq. (SI.2.24), we need to solve

$$\begin{aligned}
&\mathrm{Re}\Big\{\det\Big(M^{(k^*)}\Big)\Big\}\,\mathrm{Tr}\Big\{M^{(k^*)}\Big\}^2 = \mathrm{Im}\Big\{\det\Big(M^{(k^*)}\Big)\Big\}^2. \\
&\quad\implies \mathrm{Re}\Big\{\det\Big(M^{(k^*)}\Big)\Big\} = \frac{(\rho r_3 f_{k^*} - \rho' + \rho'')^2(\varphi^\ell - \varphi^r)^2}{(\mathrm{Tr}\{M^{(0)}\} + (2\frac{r_1 r_4}{r'} + \rho r_3(\varphi^\ell + \varphi^r))\, f_{k^*})^2} v^2 k^{*2}.
\end{aligned}$$

Since $\mathrm{Re}\Big\{\det\Big(M^{(k)}\Big)\Big\} = \det\Big(M^{(0)}\Big) + v^2 k^{*2} + g_{k^*}$, where g_k is a positive function, our problem is equivalent to find the solutions of

$$\det\Big(M^{(0)}\Big) + g_{k^*} = (c-1)v^2 k^{*2}, \tag{SI.2.25}$$

where

$$c = \frac{(\rho r_3 f_{k^*} - \rho' + \rho'')^2(\varphi^\ell - \varphi^r)^2}{(\mathrm{Tr}\{M^{(0)}\} + (2\frac{r_1 r_4}{r'} + \rho r_3(\varphi^\ell + \varphi^r))\, f_{k^*})^2}. \tag{SI.2.26}$$

Hence, if $c < 1$, the left-hand side of Eq. (SI.2.25) is positive, while the right-hand side is negative and therefore there are no real solutions for k^*. Subtracting the numerator from the

denominator,

$$
\begin{aligned}
&\left(\mathrm{Tr}\left\{M^{(0)}\right\} + \left(2\frac{r_1 r_4}{r'} + \rho r_3(\varphi^\ell + \varphi^r)\right) f_{k^*}\right)^2 - (\rho r_3 f_{k^*} - \rho' + \rho'')^2 (\varphi^\ell - \varphi^r)^2 \\
&= \left(2\frac{r_1 r_4}{r'} + \rho r_3(\varphi^\ell + \varphi^r)\right)^2 f_{k^*}^2 + 2\,\mathrm{Tr}\left\{M^{(0)}\right\}\left(2\frac{r_1 r_4}{r'} + \rho r_3(\varphi^\ell + \varphi^r)\right) f_{k^*} \\
&\quad + \mathrm{Tr}\left\{M^{(0)}\right\}^2 - \rho^2 r_3^2 f_k^2 (\varphi^\ell - \varphi^r)^2 - 2(\rho'' - \rho')\rho r_3 f_k (\varphi^\ell - \varphi^r)^2 - (\rho'' - \rho')^2 (\varphi^\ell - \varphi^r)^2, \\
&= 4\left(\frac{r_1 r_4}{r'} + \rho r_3 \varphi^\ell\right)\left(\frac{r_1 r_4}{r'} + \rho r_3 \varphi^r\right) f_k^2 + 4\,\mathrm{Tr}\left\{M^{(0)}\right\}\frac{r_1 r_4}{r'} f_k - (\rho'' - \rho')^2 (\varphi^\ell - \varphi^r)^2 \\
&\quad + 2\left(\mathrm{Tr}\left\{M^{(0)}\right\}(\varphi^\ell + \varphi^r) - (\rho'' - \rho')(\varphi^\ell - \varphi^r)^2\right)\rho r_3 f_k + \left(2r_2 + (\rho'' + \rho')(\varphi^\ell + \varphi^r)\right)^2, \\
&= 4\left(\frac{r_1 r_4}{r'} + \rho r_3 \varphi^\ell\right)\left(\frac{r_1 r_4}{r'} + \rho r_3 \varphi^r\right) f_k^2 + 4\,\mathrm{Tr}\left\{M^{(0)}\right\}\frac{r_1 r_4}{r'} f_k \\
&\quad + 4\left(r_2 + \rho''\varphi^\ell + \rho'\varphi^r\right)\left(r_2 + \rho''\varphi^r + \rho'\varphi^\ell\right) \\
&\quad - 4\left(r_2(\varphi^\ell + \varphi^r) + 2\rho'\varphi^\ell\varphi^r + \rho''((\varphi^\ell)^2 + (\varphi^r)^2)\right)\rho r_3 f_k.
\end{aligned}
$$

Since $\mathrm{Tr}\left\{M^{(0)}\right\}, f_k < 0$ the above quantity is positive. Thus, the denominator is larger than the numerator and $c < 1$. Consequently, there is no real solution for k^* such that $a_1(k^*) \cdot a_2(k^*) = 0$, and the higher order modes are all linearly stable if $k = 0$ is stable.

SI.3 Well-mixed locust model

The well-mixed limit is obtained from the spatially explicit model either when the interaction range tends to the size of the arena, or when the locusts have enough time to move around before interacting and changing direction. Remember the interactions presented in Section SI.2.2

$$
\begin{array}{ccc}
\mathcal{R} \underset{r_2}{\overset{r_1}{\rightleftarrows}} \mathcal{S} & \mathcal{R} + \mathcal{S} \xrightarrow{r_3} 2\mathcal{S} & \mathcal{R} + \mathcal{S} \xrightarrow{r_4} 2\mathcal{R} \\
\mathcal{L} \underset{r_2}{\overset{r_1}{\rightleftarrows}} \mathcal{S} & \mathcal{L} + \mathcal{S} \xrightarrow{r_3} 2\mathcal{S} & \mathcal{L} + \mathcal{S} \xrightarrow{r_4} 2\mathcal{L} \\
\mathcal{R} + \mathcal{L} \xrightarrow{r_5} \mathcal{R} + \mathcal{S} & \mathcal{R} + \mathcal{L} \xrightarrow{r_5} \mathcal{S} + \mathcal{L} & \mathcal{R} + \mathcal{L} \xrightarrow{r_6} 2\mathcal{S},
\end{array}
$$

where $\mathcal{R}$, $\mathcal{L}$ and $\mathcal{S}$ represent right-moving, left-moving and stopped locusts, respectively. While the interactions are the same, the spatial constraint has now been removed. In this approximation, any locust can interact with any other locust in the arena.

This model relates to our general model in Section SI.1 (see Table SI.1) by setting $\mathcal{R}$ and $\mathcal{L}$ to X and Y, and $\mathcal{S}$ to W with rates $a_{XY} = 0, b_{XY} = 0, c_{XY} = 0, a_{XW} = r_1, b_{XW} = r_3, c_{XW} = r_5, a_{WX} = r_2, b_{WX} = r_4, c_{WX} = 0$. In this section, we focus on deriving the drift and diffusion terms of the locust model, which we will use when comparing the data derived coefficients found using the equation-free method (Section SI.4.5) to fit parameters to data

(see Section SI.4.6 for results).

The master equation for this system can be written as

$$\frac{\partial p(\boldsymbol{x};t)}{\partial t} = \sum_{j=1}^{5} \left\{ \varrho_j(\boldsymbol{x}-\nu_j)p(\boldsymbol{x}-\nu_j;t) - \varrho_j(\boldsymbol{x})p(\boldsymbol{x};t) \right\}, \tag{SI.3.1}$$

where $\boldsymbol{x} = (x, y)$ is the number of right and left movers, respectively, and

$$\begin{aligned}
\varrho_1(x,y) &= xr_1 + x(N-x-y)\frac{r_3}{N} + xy\frac{r_5}{N}, & \nu_1 &= (-1,0),\\
\varrho_2(x,y) &= yr_1 + y(N-x-y)\frac{r_3}{N} + xy\frac{r_5}{N}, & \nu_2 &= (0,-1),\\
\varrho_3(x,y) &= (N-x-y)r_2 + x(N-x-y)\frac{r_4}{N}, & \nu_3 &= (1,0),\\
\varrho_4(x,y) &= (N-x-y)r_2 + y(N-x-y)\frac{r_4}{N}, & \nu_4 &= (0,1),\\
\varrho_5(x,y) &= xy\frac{r_6}{N}, & \nu_5 &= (-1,-1).
\end{aligned}$$

The Fokker-Planck approximation for the densities of right and left movers is

$$\begin{aligned}
\frac{\partial p(x,y;t)}{\partial t} =& -\frac{\partial}{\partial x}\Big(F_1(x,y)p(x,y;t)\Big) - \frac{\partial}{\partial y}\Big(F_2(x,y)p(x,y;t)\Big)\\
&+ \frac{1}{2N}\frac{\partial^2}{\partial x^2}\Big(D_{1,1}(x,y)p(x,y;t)\Big) + \frac{1}{2N}\frac{\partial^2}{\partial y^2}\Big(D_{2,2}(x,y)p(x,y;t)\Big)\\
&+ \frac{1}{N}\frac{\partial^2}{\partial x \partial y}\Big(D_{1,2}(x,y)p(x,y;t)\Big),
\end{aligned}$$

where

$$\begin{aligned}
F_1(x,y) &= (1-x-y)r_2 - xr_1 - x(1-x-y)(r_3-r_4) - xy(r_5+r_6),\\
F_2(x,y) &= (1-x-y)r_2 - yr_1 - y(1-x-y)(r_3-r_4) - xy(r_5+r_6),\\
D_{1,1}(x,y) &= (1-x-y)r_2 + xr_1 + x(1-x-y)(r_3+r_4) + xy(r_5+r_6),\\
D_{2,2}(x,y) &= (1-x-y)r_2 + yr_1 + y(1-x-y)(r_3+r_4) + xy(r_5+r_6),\\
D_{1,2}(x,y) &= xyr_6.
\end{aligned} \tag{SI.3.2}$$

Note that $F_2(x,y) = F_1(y,x)$ and $D_{2,2}(x,y) = D_{1,1}(x,y)$. In the deterministic limit, $N \to \infty$, we get

$$\begin{aligned}
\frac{\mathrm{d}x}{\mathrm{d}t} &= F_1(x,y),\\
\frac{\mathrm{d}y}{\mathrm{d}t} &= F_2(x,y).
\end{aligned} \tag{SI.3.3}$$

To get the deterministic steady states of the system, we set $F_1(x,y) = F_2(x,y) = 0$ and define

$r' = r_4 - r_3$ and $r'' = r_5 + r_6$,

$$\begin{aligned} (1-x-y)r_2 - xr_1 + x(1-x-y)r' - xyr'' &= 0 \\ (1-x-y)r_2 - yr_1 + y(1-x-y)r' - xyr'' &= 0, \end{aligned} \tag{SI.3.4}$$

Subtracting the first from the second, and remembering that $w = 1 - x - y$,

$$(x-y)(r_1 + wr') = 0 \Rightarrow x = y \text{ or } w^* = \frac{r_1}{r_4 - r_3}. \tag{SI.3.5}$$

If $x = y$, then

$$\begin{aligned} (1-2x)r_2 - xr_1 + x(1-2x)r' - x^2r'' &= 0 \\ x^2(r'' + 2r') + x(r_1 + 2r_2 - r') - r_2 &= 0. \end{aligned}$$

If $r'' = -2r'$,

$$x = y = \frac{r_2}{r_1 + 2r_2 - r'} \quad w = \frac{r_1 - r'}{r_1 + 2r_2 - r'}, \tag{SI.3.6}$$

while if $r'' \neq -2r'$, then

$$x = \frac{(2r_2 + r_1 - r') \pm \sqrt{(2r_2 + r_1 - r')^2 + 4r_2(2r' + r'')}}{2(-2r' - r'')}. \tag{SI.3.7}$$

If $-2r' - r'' < 0$, then there is a positive solution of order r_2, for small r_2, and a non-physical negative solution.

SI.3.1 Stopped locusts promote alignment

On the other hand, if $w = w^* = \frac{r_1}{r_4 - r_3}$, then

$$xyr'' - r_2w^* = 0 \Rightarrow xy = \frac{r_2w}{r''}. \tag{SI.3.8}$$

Using $y = 1 - w^* - x$ gives

$$x^2 - x(1-w^*) + \frac{r_2}{r''}w^* = 0 \Rightarrow x = \frac{(1-w^*) \pm \sqrt{(1-w^*)^2 - 4\frac{r_2w^*}{r''}}}{2}. \tag{SI.3.9}$$

Since the square root factor is smaller than $(1 - w)$, we get two positive solutions when they exist. Moreover, $x_+ + x_- + w = 1$. The solution is not physical if $w^* < 0$, $w^* > 1$ or if (remember $r' = r_4 - r_3$ and $r'' = r_5 + r_6$)

$$r_2 \geq \left(1 + \frac{r_1}{r'}\right)^2 \frac{r'r''}{4r_1}. \tag{SI.3.10}$$

That means that, in the limit $N \to \infty$, we will see some non-zero alignment $x - y = \pm\sqrt{(1 - \frac{r_1}{r'})^2 - 4\frac{r_2 r_1}{r'' r'}}$. From the stability analysis performed in Section SI.2, we note that the aligned solution is always stable when it exists inside the simplex, while the disordered solution is stable otherwise.

It is important to highlight that the role of the third-order interactions in Dyson et al. [2015]'s model was to specifically create this alignment when $N \to \infty$, while keeping the zero alignment unstable, allowing the noise to create bistability when N is small. This same property is obtained in this model with three stances without the need for third-order interactions.

Furthermore, Eq. (SI.3.10) provides insight into ways to break coherence in the system. For instance, one option is to cause external disturbance to increase r_2, the rate at which the stopped locusts spontaneously start moving. Other options are to keep r_2 constant but decrease r_5 or r_6 by external perturbations (e.g., introducing predators in the environment that will chase the locusts), thus reducing the number of stopped locusts. The mechanisms behind destabilising the swarm suggest that neutral actors play a major role in stabilising the swarm and facilitating alignment.

For generality and to compare results between different data sets, we will consider $r_6 = 0$ onwards unless stated otherwise. As our results here and in Section SI.2 show, r_6 and r_5 play similar roles in the dynamics through the combined parameter $r'' = r_5 + r_6$, meaning we can incorporate r_6 into r_5 without significantly impacting any of our results. In this case, the well-mixed locust model is a subset of the general model introduced and analysed in Section SI.1. All results on the stochastic model, including submanifolds, stationary probability distributions and mean transit times, are also valid for the locust model with the appropriate parameter mapping.

SI.4 Locust data analysis

In this section, we are going to present and analyse the data from locusts experiments performed by Buhl et al. [2006]. We will focus on using this data to test the predictions of our theoretical framework in terms of the observed submanifold of the data and the stationary probability distributions. We will also test how well the spatial features of the locust experimental data can be approximated by the well-mixed modelling approach.

Note on notation: Given the relationship between this section and Section SI.2, we will share the same notation in which x and y are coordinates in physical space. We use R to denote an interaction radius, L is the length of the arena, and r, ℓ and s to represent the proportions of clockwise, anticlockwise and stopped locusts, respectively.

SI.4.1 Data cleaning protocol

The raw data provides the x coordinate, y coordinate and size for each object detected in each frame in units of pixels, sorted by y coordinate. The movie frame rate from which the data is extracted from is 5 frames per second, and the recordings last 6 to 8 hours. We performed a cleaning process to track each object in each frame, even when it was occluded. This cleaning process corresponded to three stages: a manual and specific pre-cleaning for each experiment, an algorithmic tracking, and a stance classification of the objects.

Pre-cleaning: In a case-by-case process, we manually determined the maximum number of objects that could be correctly interpreted as locusts in the arena, N (i.e., not missing or massive objects corresponding to occluded locusts). We also deleted any objects detected outside the arena and 'shadows or spots' – objects much smaller than the average size of a locust, and positioned close to average-sized objects. We then defined a 'warm-up' period, in which the locusts are still becoming familiar with their experimental environment and starting to move more consistently. This warm-up period is consistent with definitions of Buhl et al. [2006]. The data from the warm-up period is discarded from the analysis. We then selected an initial frame containing as much information as possible about the positions of all locusts in the arena.

Algorithmic-tracking: We remove all data from frames corresponding to the 'warm-up' period. Given the determined number of locusts in the experiment, N, and an initial frame with information about the position and size of all objects deemed locusts, the algorithm tracks each object in subsequent frames as the closest object in the new frame. Algorithmically, this corresponds to:

1. Get the positions of the objects in the previous, cleaned, frame $t-1$ and in the uncleaned frame, t.
2. Compute all the distances between each pair of objects (i, j), where i is an object of frame $t-1$ and j is an object of frame t.
3. Check the number of objects in frame t, n_t.
4. If $n_t = N$, all objects have been detected in frame t.
   (a) Successively, select the remaining pair (i, j) with the smallest distance, and give object j object i's id.
   (b) Remove object i from the pool of unmatched objects by removing all pairs with object i.
5. If $n_t < N$, there are occluded objects.
   (a) Match the available objects in frame t as in the previous step.

(b) Determine objects in t much larger than the average object size and add them to a list of 'candidate objects'.

(c) For each unmatched object in frame $t-1$, find a 'candidate object' from the list with the smallest distance.

(d) If the displacement between the unmatch object and the candidate is smaller than 30 units (about 10 times larger than the average displacement of moving locusts per frame), match the two objects by repeating the candidate object's data in frame t. Otherwise, the displacement is too large, and it might be the case that the unmatched object was not detected in frame t. Hence, copy the unmatched object's data from frame $t-1$ into frame t.

This way, all original data in frame t has been kept, while occluded objects will have replicated data from the frame to indicate there are many objects in that location, keeping track of all objects in all frames.

Stance classification: The classification of each object in each frame as 'anticlockwise', 'clockwise' or 'stopped' is based on the moving average of their angular displacement between frames t and $t + t_w$. The moving average reduces the effect of measurement errors and fluctuations in the movement of the locusts. We used a time window, t_w, of 10 frames (2 seconds). We considered a locust immobile if their angular displacement was less than 0.005 rad/frame and otherwise moving, in line with the definition in [Buhl et al., 2006].

SI.4.2 Interaction radius estimation

In Section SI.2 the ratio between the particles' interaction range and the arena's size was important to scale the interaction rates correctly. To compare the spatial and well-mixed models in realistic cases, we require estimates of the interaction range of the locusts in the arena.

We estimate the interaction radius by looking at the behaviour of the probability a locust changes direction when isolated as a function of the interaction radius, R. This quantity should be independent of the interaction radius, such that abrupt changes in the behaviour of the curves suggest that external factors (such as interactions with other locusts in the arena not captured within the defined neighbourhood) influence the estimate of the spontaneous change probability. The use of the spontaneous rate of change is due to the lack of ambiguity when defining an 'isolated locust' when compared to other interactions that become ambiguous when 3 or more locusts are within the radius of each other.

Computationally, this is estimated as the ratio between the number of frames a locust changed direction after being isolated and the number of frames that it has been detected as isolated. For each given radius, R, isolation means the absence of locusts within a distance smaller

than R. In Fig. SI.5 we plot this probability for different group sizes as a function of R. For all experiments, there is an abrupt change in behaviour at around $R = 20$ cm, in agreement with experimental observations [Buhl et al., 2006].

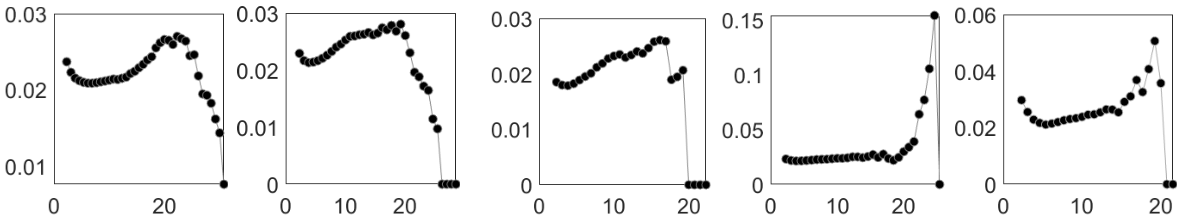

(a) $N = 30$ (b) $N = 50$ (c) $N = 70$ (d) $N = 80$ (e) $N = 100$

Figure SI.5: The probability of isolated locusts changing stances as a function of a given interaction radius, R (plotted in the x-axis, in cm), for different group sizes. Abrupt changes occur at $R \approx 20$ cm, suggesting this value is a good approximation to the interaction radius of the locusts. This agrees with observations by Buhl et al. [2006].

SI.4.3 Comparing SPDs of the well-mixed and spatial models

We use the Wasserstein metric (also known as Earth Mover's distance) [Rubner et al., 1998] to compare the 2-dimensional probability distributions of the well-mixed and spatial models to measure how "well-mixed" is a system with interaction radius, R, and moving locust speed, v.

The Wasserstein metric is preferred over others, such as the Hellinger or the Kullback–Leibler divergence, since we compare discrete probability distributions obtained from numerical simulations, meaning some entries might be zero. Moreover, the Wasserstein metric is consistent with the intuition that distributions resulting from small changes in parameter values are not significantly different, only slightly changing the position of the peaks of the distribution while maintaining its shape.

Let $P_{R,v}(r, \ell)$ be the 2-dimensional stationary probability distribution that the system has a proportion of r clockwise and ℓ anticlockwise moving locusts of the spatial system with radius R and speed v, and $P_{WM}(r, \ell)$ the same for the well-mixed system. We define the "well-mixedness", $\mathcal{W}(R, v)$ as

$$\mathcal{W}(R, v) = 1 - W\left(P_{WM}, P_{R,v}\right), \tag{SI.4.1}$$

where $W\left(P_{WM}, P_{R,v}\right)$ is the Wasserstein metric between P_{WM} and $P_{R,v}$ normalised by the maximum distance on the simplex. Since we are comparing two normalised probability distributions (same mass), normalising by the maximum distance on the simplex guarantees that $W\left(P_{WM}, P_{R,v}\right)$ is between 0 and 1.

Fig. SI.6 shows how $\mathcal{W}(R, v)$ changes as a function of R and v. To generate the figure we

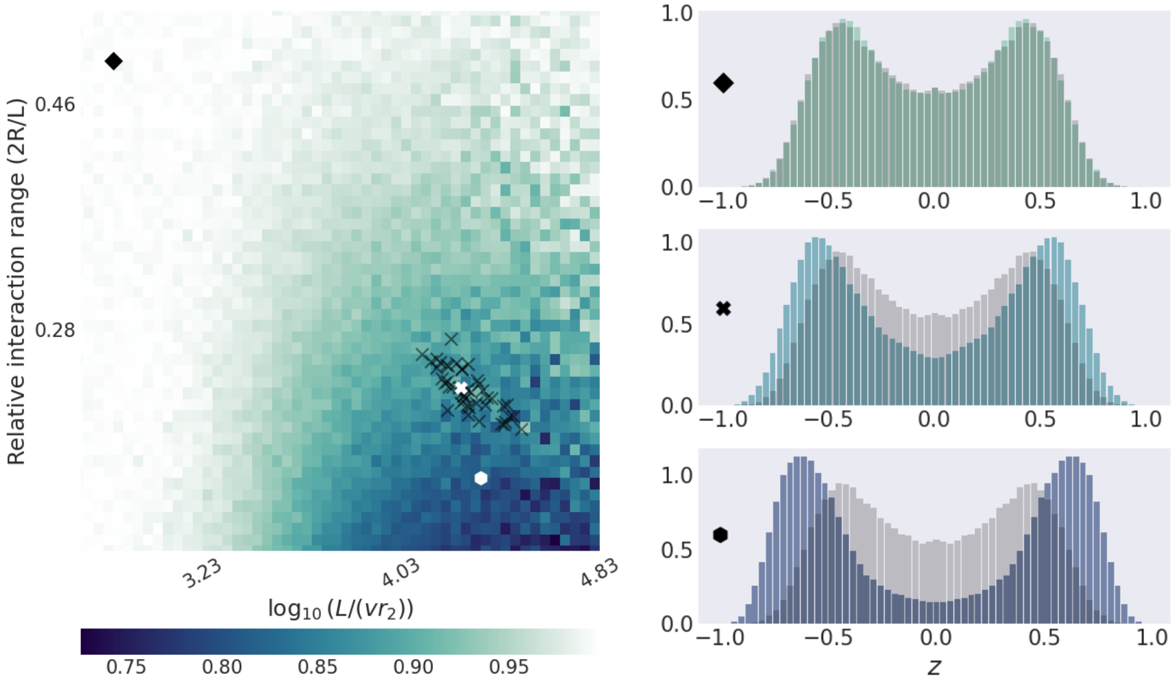

(a) (b)

Figure SI.6: Locust behaviour shows no significant spatial correlation during consensus or switching, confirming that a well-mixed model accurately captures the collective dynamics. (a) The “well-mixedness” of the spatial model is based on the Earth Mover’s Distance between the 2-dimensional stationary probability distributions of the well-mixed and spatial models for varying locust speed, v, and range of interaction, R. Lighter colours indicate better agreement between the well-mixed and spatial models. Thin black crosses correspond to estimates of the speed and interaction range of the locusts for different experiments. For both models, simulations have $N = 30$ particles and parameters $r_1 = 0.0999$, $r_2 = 0.0148$, $r_3 = 0.03504$, $r_4 = 0.2370$, $r_5 = 0.3480$, corresponding to averaged fitted parameters from experiments with $25 - 36$ locusts. (b) Examples of comparisons between the one-dimensional stationary probability distributions for three representative choices of the speed and interaction radius of the spatial model (see (a)). The second panel, marked with the white cross, illustrate the comparison of parameters representative of the real locust experiments (see (a)).

used stochastic simulations of the spatial model with fixed $N = 30$ particles, and parameters $r_1 = 0.0999$, $r_2 = 0.0148$, $r_3 = 0.03504$, $r_4 = 0.2370$, $r_5 = 0.3480$, corresponding to averaged fitted parameters from experiments with $25 - 36$ locusts. In the right panel, we plotted the projection of the 2-dimensional SPDs along the lines of constant $z = r - \ell$ for a few different values to provide a visual comparison of the differences between the well-mixed (grey) and the spatial (colours) SPDs.

The crosses correspond to the estimated ratio $2R/L$ and speeds for experimental data. We used $R = 20$ cm and estimated L as the average circumference travelled by the locusts in the corresponding experiment. We estimated L/v as the average angular velocity of the

moving locusts in the experiment. At values of "well-mixedness" around 0.82, the dynamics of the locusts in the experiments are well-characterised by the well-mixed model, obtaining qualitatively similar SPDs.

SI.4.4 Two-body correlations

In the locust system, it could be hypothesised that the locusts collide and remain stationary, blocking the movement of other locusts in the arena and forming agglomerates of stopped locusts that eventually change direction. Hence, the observed increase in the number of stopped locusts as they switch directions would be caused by a spatial effect. This means that a well-mixed approximation would not be appropriate for describing the dynamics of the locusts' experiment. In this section, we test whether large groups of stopped locusts form by analysing the distribution of two-body correlations [Younge et al., 2006, Gavagnin et al., 2018] in the experimental data.

To check for significant spatial correlations when the system is transitioning between consensus states, we compute two-body spatial correlations in two groups of frames. In the first group, we considered 'strong consensus' frames, in which a strong majority of anticlockwise or clockwise moving locusts is observed, i.e., $|n_r - n_\ell| \geq 0.6N$, for n_r the number of clockwise moving locusts, n_ℓ the number of anticlockwise moving locusts, and N the group size of the experiment. The second group of frames is 'transition' frames, in which no consensus is observed. These are frames in which $|n_\ell - n_r| \leq 1$.

For each group of frames, we looked at the angular distance between pairs of locusts in the frame. For both the 'consensus' and 'transition' frames, we were interested in the distances between any two moving locusts or any two stopped locusts to check that they were not grouping as they continued to move and that the locusts were not stopping due to collisions with a large group of stopped locusts. Computing distances between pairs of locusts with the same stance also guarantees we are considering frames of reference in which the locusts are stationary to each other. We then aggregated the data for all frames in the same group and plotted histograms as presented in Figs. SI.7 and SI.8. The figures indicate that changes in stance from stopped to moving and vice versa occur throughout the arena and the absence of strong spatial correlations in directional switches, supporting the adoption of the well-mixed model approximation.

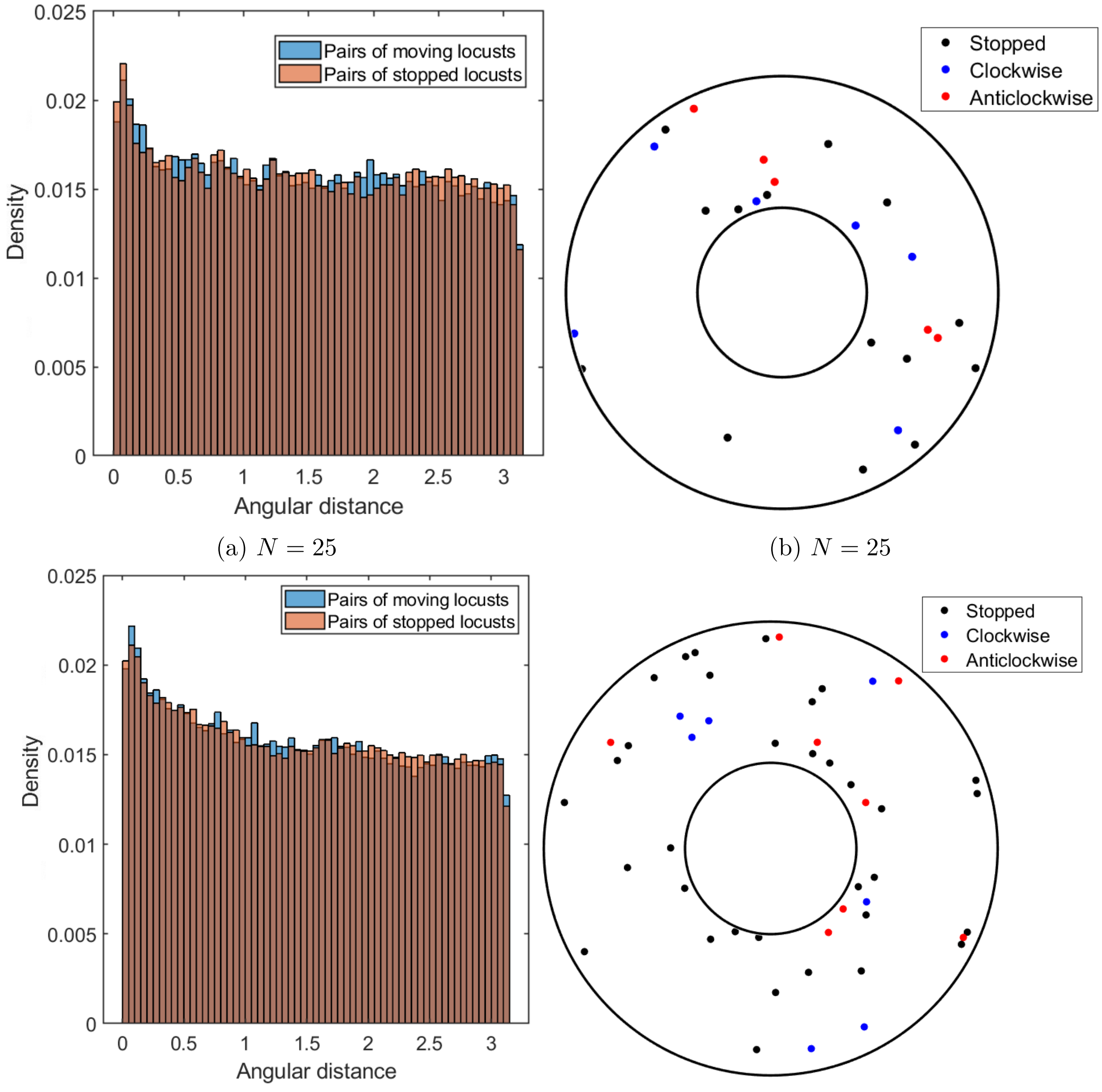

(a) $N = 25$

(b) $N = 25$

(c) $N = 50$

(d) $N = 50$

Figure SI.7: Stopped locusts do not form spatial clusters during low-polarisation phases, confirming that increased neutrality reflects a genuine behavioural mechanism rather than a local crowding effect. Panels (a) and (c) show pairwise distances between locusts in frames with low polarisation, in which the difference between the number of anticlockwise and clockwise moving locusts is 1 or less. Pairwise distance distributions between pairs of stopped locusts (orange) and pairs of locusts moving in the same direction (i.e., both moving clockwise or anticlockwise, blue histogram) for experiments with 25 (a) and 50 locusts (c). The histograms suggest that moving and stopped locusts are found everywhere in the arena, with a small peak at short distances when they collide. Panels (b)-(d) show sample frames used for the histograms depicting the position of the locusts in the arena for a group of $N = 25$ locusts (b) and $N = 50$ locusts (d). Two occluded locusts are not distinguishable in (d) due to high population density and low equipment resolution (see SM 4.1 for details on data cleaning protocols). The figures indicate the absence of strong spatial correlations in directional switches, supporting the adoption of the well-mixed model approximation.

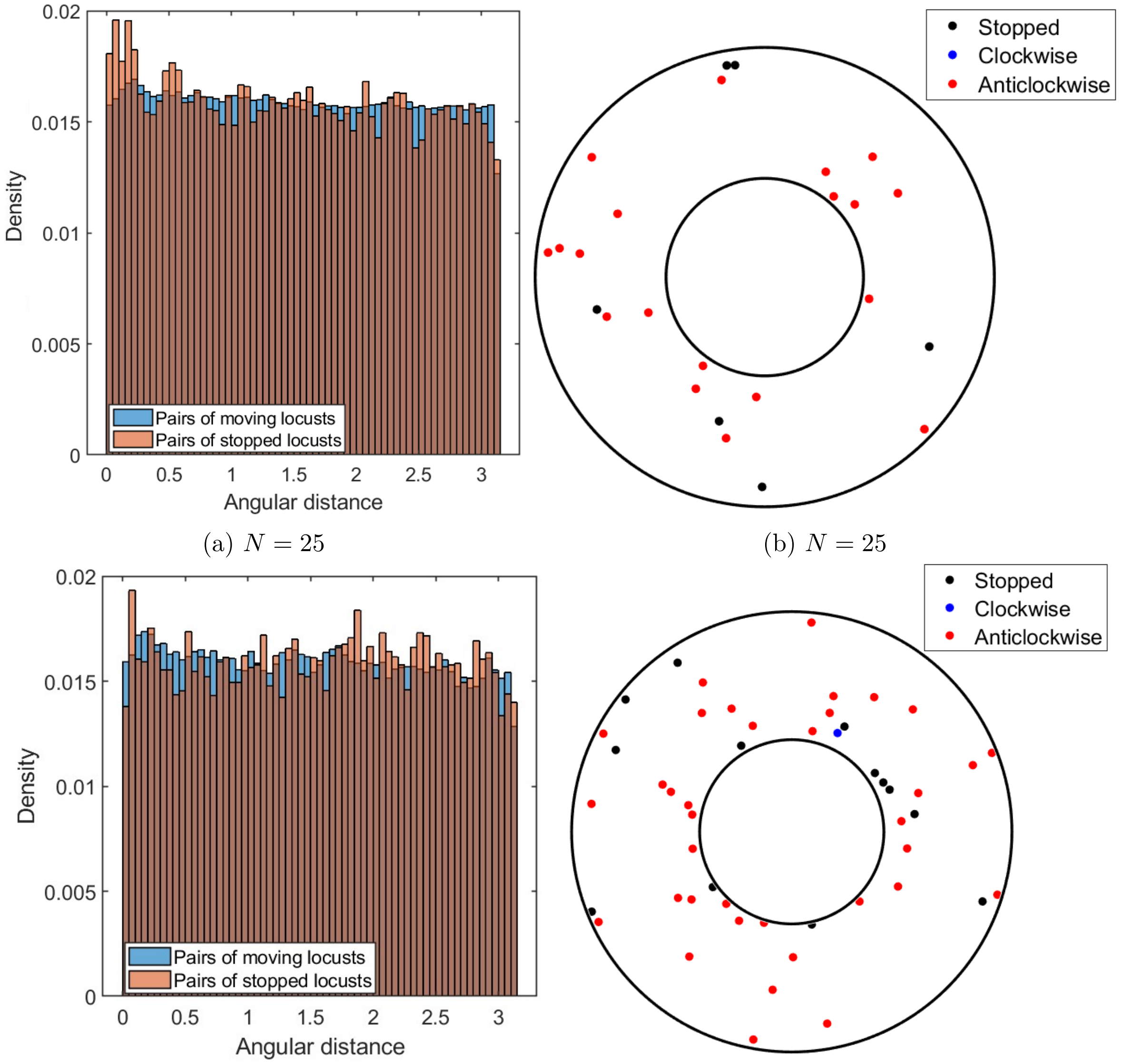

(a) $N = 25$ (b) $N = 25$

(c) $N = 50$ (d) $N = 50$

Figure SI.8: Locusts do not form directional clusters during strong consensus, confirming that collective alignment arises from distributed interactions rather than spatial crowding. Panels (a) and (c) show pairwise distances between locusts in frames with high polarisation, in which the difference between the number of anticlockwise and clockwise moving locusts is at least 60% of the total group size. Pairwise distance distributions between pairs of stopped locusts (orange) and pairs of locusts moving in the same direction (i.e., both moving clockwise or anticlockwise, blue histogram) for experiments with 25 (a) and 50 locusts (c). Panels (b)-(d) show sample frames used for the histograms depicting the position of the locusts in the arena for a group of $N = 25$ locusts (b) and $N = 50$ locusts (d). The figures indicate the absence of strong spatial correlations in directional switches. Rather, changes in stance from stopped to moving and vice versa occur throughout the arena, supporting the adoption of the well-mixed model approximation.

SI.4.5 Equation-free method in higher dimensions

Having established the well-mixed model as a good approximation to the more complicated spatial dynamics of the experiments with locusts, we aim to fit the parameters of the model to experimental data to determine if our model can describe the experimental data and get insight into the most significant interactions.

The equation-free method is a powerful tool to derive estimates of the drift and diffusion of stochastic differential equations from data [Yates et al., 2009, Dyson et al., 2015]. Here, we extend the theory to higher-dimensional models. Consider the Fokker-Planck equation in a dimension $d \geq 1$:

$$\frac{\partial p(\boldsymbol{x},t)}{\partial t} = -\sum_{i=1}^{d} \frac{\partial}{\partial x_i}[F_i(\boldsymbol{x})p(\boldsymbol{x},t)] + \sum_{i=1}^{d}\sum_{j=1}^{d} \frac{\partial^2}{\partial x_i \partial x_j}[D_{ij}(\boldsymbol{x})p(\boldsymbol{x},t)], \tag{SI.4.2}$$

with drift vector $\boldsymbol{F} = (F_1, F_2, \dots, F_d)^T$ and diffusion tensor $\mathbf{D} \in \mathbb{R}^d \times \mathbb{R}^d$. This Fokker-Planck equation has an associated Itô SDE representation of the form

$$d\boldsymbol{X}_t = \boldsymbol{F}(\boldsymbol{X}_t)dt + \boldsymbol{\sigma}(\mathbf{X}_t)d\mathbf{W}_t, \tag{SI.4.3}$$

where $\mathbf{X}_t$ and $\boldsymbol{F}(\mathbf{X}_t)$ are d-dimensional random vectors, $\mathbf{W}_t$ is an m-dimensional Wiener process, and $\boldsymbol{\sigma}$ is a $d \times m$ matrix such that $\mathbf{D} = \frac{1}{2}\boldsymbol{\sigma}\boldsymbol{\sigma}^T$, i.e.,

$$D_{ij}(\boldsymbol{x}) = \frac{1}{2}\sum_{k=1}^{d} \sigma_{ik}(\boldsymbol{x})\sigma_{jk}(\boldsymbol{x}). \tag{SI.4.4}$$

In this case, $p(\boldsymbol{x}, t)$ is then the probability density for the process $\mathbf{X}_t$. One can use the multidimensional Euler-Maruyama method to compute approximate trajectories that obey Eq. (SI.4.3), i.e.,

$$X_i(t+\delta t) - X_i(t) = F_i(\mathbf{X}(t))\delta t + \sum_{j=1}^{m} \sigma_{ij}(\mathbf{X}(t))\sqrt{\delta t}\xi_j(t), \tag{SI.4.5}$$

where δt is the computational time-step, $\xi_j(t)$ are independent and identically distributed

$\mathcal{N}(0,1)$ random variables. As $\delta t \to 0$, $\mathbf{X}(t) \to \mathbf{X}_t$. Hence,

$$\left\langle \frac{X_i(t+\delta t) - x_i}{\delta t} \right\rangle = F_i(\boldsymbol{x}) + \frac{1}{\sqrt{\delta t}} \sum_{j=1}^{m} \sigma_{ij}(\boldsymbol{x}) \langle \xi_j(t) \rangle, \tag{SI.4.6}$$

$$\begin{aligned}\left\langle \frac{(X_i(t+\delta t) - x_i)(X_j(t+\delta t) - x_i)}{\delta t} \right\rangle =& F_i(\boldsymbol{x}) F_j(\boldsymbol{x}) \delta t + \sqrt{\delta t} F_i(\boldsymbol{x}) \sum_{k=1} \sigma_{ik}(\boldsymbol{x}) \langle \xi_k(t) \rangle \\ &+ \sqrt{\delta t} F_j(\boldsymbol{x}) \sum_{k=1} \sigma_{jk}(\boldsymbol{x}) \langle \xi_j(t) \rangle \\ &+ \sum_{\ell=1}^{m} \sum_{k=1}^{m} \sigma_{i\ell}(\boldsymbol{x}) \sigma_{jk}(\boldsymbol{x}) \langle \xi_\ell(t) \xi_k(t) \rangle,\end{aligned} \tag{SI.4.7}$$

where $\langle \,\cdot\, \rangle$ is the average given $\mathbf{X}(t) = \boldsymbol{x}$.

Theoretically, $\langle \xi_k(t) \rangle = 0$, $\langle \xi_\ell(t) \xi_k(t) \rangle = \delta_{\ell k}$, where $\delta_{\ell k}$ is the Kronecker delta. Thus,

$$F_i(\boldsymbol{x}) = \lim_{\delta t \to 0} \left\langle \frac{X_i(t+\delta t) - x_i}{\delta t} \right\rangle \tag{SI.4.8}$$

$$D_{ij} = \frac{1}{2} \lim_{\delta t \to 0} \left\langle \frac{(X_i(t+\delta t) - x_i)(X_j(t+\delta t) - x_i)}{\delta t} \right\rangle = \frac{1}{2} \sum_{k=1}^{m} \sigma_{ik}(\boldsymbol{x}) \sigma_{jk}(\boldsymbol{x}). \tag{SI.4.9}$$

In practice, our sample sizes and time steps are finite, hence, the computational value of $\langle \xi_k(t) \rangle$ might not be zero as the result only holds in the limit that both sample sizes tend to infinity and time steps tend to zero. This means that we need to be careful to pick a computational δt for our evaluation of $F_i(\boldsymbol{x})$ that is not too small in order to avoid the diffusion 'bleeding through' in Eq. (SI.4.8). For the diffusion, the smaller the δt we choose, the better. In our case, the frame rate of the data is not too large, meaning that δt is not too small, and we might benefit from choosing δt to be the smallest possible in both cases, i.e., 1 frame or 0.2 seconds.

SI.4.6 Fitting parameters to data using equation-free method

Using the equation-free method described in the previous section with parameters $\delta t = 0.2$ seconds (1 frame), we obtain an estimate of the drift and diffusion of in our $d = 2$ system, which we can use to fit the functional forms of Eq. (SI.3.2) using weighted minimum squared error. Let $\tilde{F}_1(x,y)$, $\tilde{F}_2(x,y)$, $\tilde{D}_{1,1}(x,y)$, $\tilde{D}_{1,2}(x,y)$, $\tilde{D}_{2,1}(x,y)$ and $\tilde{D}_{2,2}(x,y)$ be the estimates of the drift and diffusion terms for the point (x,y) in the data set obtained using the equation-free method. We define the weighted error function of the set of parameters

$\{r_i\} \coloneqq \{r_1, r_2, r_3, r_4, r_5\}$.

$$\begin{aligned}\mathrm{Er}(\{r_i\}) = \sum_{(x,y)} &\omega_{f_1}(x,y) \left\{ \tilde{F}_1(x,y) - F_1(x,y \mid \{r_i\}) \right\}^2 \\ &+ \omega_{f_2}(x,y) \left\{ \tilde{F}_2(x,y) - F_2(x,y \mid \{r_i\}) \right\}^2 \\ &+ \sqrt{\omega_{d_{1,1}}(x,y)} \left\{ \tilde{D}_{1,1}(x,y) - D_{1,1}(x,y \mid \{r_i\}) \right\}^2 \\ &+ \sqrt{\omega_{d_{2,2}}(x,y)} \left\{ \tilde{D}_{2,2}(x,y) - D_{2,2}(x,y \mid \{r_i\}) \right\}^2 \\ &+ \sqrt{\omega_{d_{1,2}}(x,y)} \left\{ \tilde{D}_{1,2}(x,y) - D_{1,2}(x,y \mid \{r_i\}) \right\}^2 \\ &+ \sqrt{\omega_{d_{2,1}}(x,y)} \left\{ \tilde{D}_{2,1}(x,y) - D_{2,1}(x,y \mid \{r_i\}) \right\}^2 ,\end{aligned} \tag{SI.4.10}$$

where ω's are the counts of the bin (x, y) in the data for that particular term, meaning that data points with more counts have more weight on the fitting. $F_i(x, y \mid \{r_i\})$ and $D_{i,j}(x, y \mid \{r_i\})$ are the functional terms from Eq. (SI.3.2) in which we made explicit the dependency on the set of parameters $\{r_i\}$. Note that not all possible pairs (x, y) are present in the data, hence, the sum is only over the observed pairs.

We then minimise $\mathrm{Er}(\{r_i\})$ using MATLAB's `particleswarm` function with tolerance `1e-9` to perform the particle swarm minimisation algorithm [Kennedy and Eberhart, 1995, Kennedy, 2006, Bonyadi and Michalewicz, 2017]. Table SI.2 shows the parameter values obtained from the fitting. Figs. SI.9 to SI.14 depict the data overlaid with the fitted fast flow and analytical stationary probability distributions derived from the curved manifold approximation. The external flow field over the simplex obtained for the fitted parameters matches the apparent distribution of the data, capturing the dynamics precisely. The analytical approximation of the curved slow manifold is better at fitting larger group sizes, in which the assumptions of ignoring terms of order ε/N or higher are satisfied more appropriately.

Experiment	$r_1 = a_{XW}$	$r_2 = a_{WX}$	$r_3 = b_{XW}$	$r_4 = b_{WX}$	$r_5 = c_{XW}$
5_28	0.28980	0.03206	0.00000	0.06745	0.00308
5_29	0.13234	0.03388	0.00000	0.19003	0.11453
5_36	0.04519	0.02755	0.10115	0.18880	0.28492
5_45	0.06810	0.02704	0.00000	0.23020	0.13469
5_46	0.03473	0.03062	0.18584	0.11937	0.35520
7_14	0.12977	0.03164	0.03682	0.11861	0.10007
7_27	0.03960	0.02033	0.10032	0.13750	0.17910
7_30	0.07130	0.03537	0.06637	0.16852	0.33244
7_35	0.02275	0.03487	0.13267	0.17003	0.26235
7_8	0.07910	0.06121	0.05585	0.11711	0.20616
10_1	0.03916	0.06404	0.04766	0.25239	0.22686
10_31	0.07999	0.02312	0.06551	0.16653	0.21137
10_40	0.04982	0.02746	0.00000	0.15763	0.20018
10_9	0.06950	0.08162	0.09885	0.18361	0.21915
11_18	0.03354	0.07136	0.11472	0.08970	0.22611
15_13	0.07884	0.03030	0.11766	0.11258	0.25720
15_32	0.06234	0.01961	0.03125	0.23712	0.19479
15_6	0.08571	0.03130	0.03630	0.21721	0.34249
19_33	0.06782	0.01926	0.01185	0.17093	0.28416
20_12	0.11269	0.03630	0.04582	0.07834	0.31947
20_3	0.02639	0.06734	0.18341	0.35138	0.37095
21_5	0.04531	0.02962	0.16208	0.27578	0.32800
22_15	0.09087	0.02052	0.00000	0.18165	0.28937
25_17	0.04702	0.04319	0.06531	0.20759	0.23353
25_20	0.09629	0.03064	0.01669	0.23697	0.22248
25_34	0.04816	0.01513	0.03512	0.23704	0.27525
25_4	0.09576	0.03699	0.11205	0.31158	0.38388
26_10	0.08740	0.03201	0.00000	0.19999	0.21331
29_22	0.06593	0.02679	0.05675	0.25107	0.16861
30_11	0.08681	0.02276	0.00000	0.20869	0.34972
30_21	0.11482	0.02618	0.00000	0.22610	0.28778
32_50	0.03600	0.01156	0.10311	0.26768	0.19069
34_23	0.06761	0.02885	0.00000	0.23718	0.33391
36_24	0.05224	0.03936	0.04849	0.20319	0.29027
39_48	0.02962	0.03188	0.10829	0.26484	0.17259
41_37	0.03620	0.02453	0.03671	0.28569	0.19888
42_7	0.05346	0.02517	0.02324	0.20635	0.27492
47_25	0.04964	0.03393	0.06616	0.24926	0.31069
49_16	0.04386	0.03248	0.05885	0.18429	0.25568
50_38	0.04833	0.01118	0.10643	0.25064	0.35134
66_26	0.03590	0.03452	0.00000	0.30705	0.20117
70_49	0.04117	0.03725	0.06111	0.26832	0.21717
80_43	0.03875	0.02941	0.11961	0.24687	0.32064
100_0	0.06591	0.07812	0.05925	0.34461	0.33765

Table SI.2: Parameters fitted for the locust data set using the equation-free method.

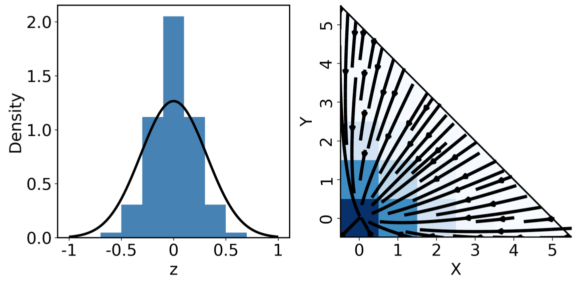

(a) Experiment ‘5_28’

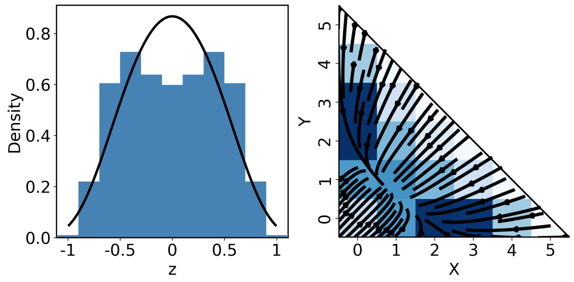

(b) Experiment ‘5_29’

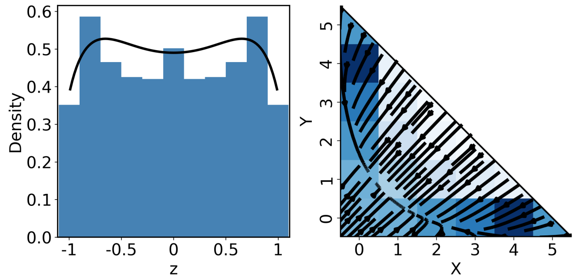

(c) Experiment ‘5_36’

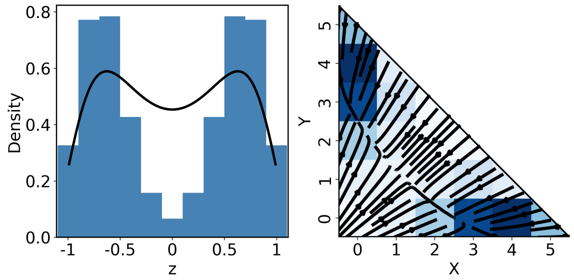

(d) Experiment ‘5_45’

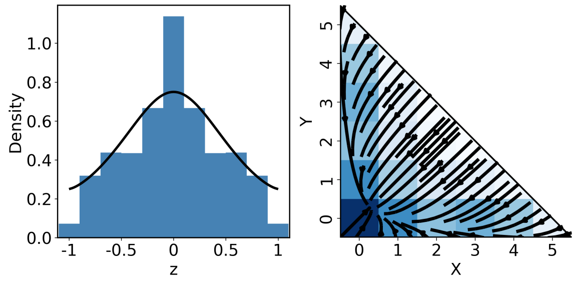

(e) Experiment ‘5_46’

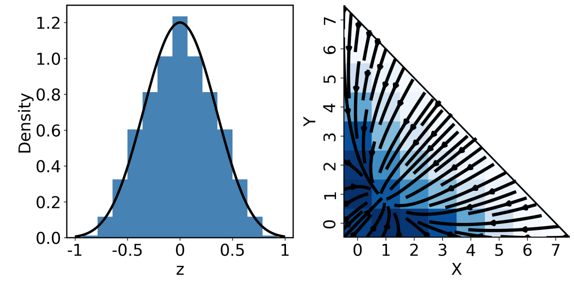

(f) Experiment ‘7_14’

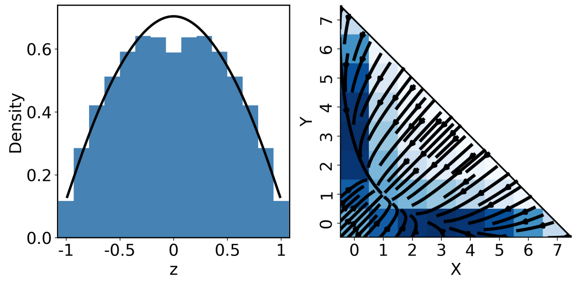

(g) Experiment ‘7_27’

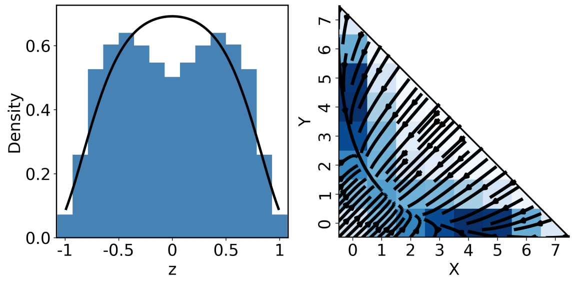

(h) Experiment ‘7_30’

Figure SI.9: (a-h) (Right) Heat maps of the occupied system states for different experiments with locusts. Plotted over the heat maps are the flow fields obtained from our model for individually fitted parameters (see Table SI.2 for parameter values). In all cases, the flow field captures the position and curvature of the slow manifold observed in the data. (Left) The projection of the two-dimensional stationary probability distributions (right side) into $z = (X - Y)/N$. The black curve over the histograms is the analytical SPD for z obtained from the curved manifold approximation (see Eq. (SI.1.33)).

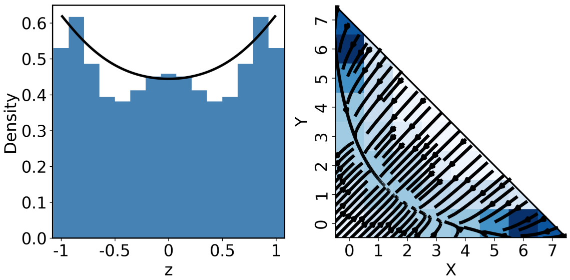

(a) Experiment '7_35'

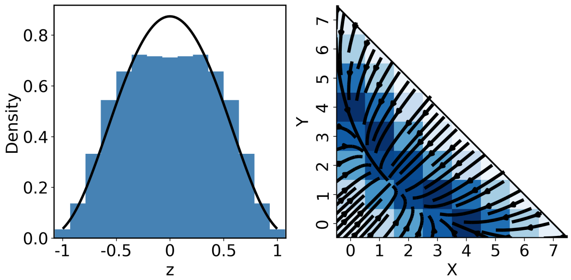

(b) Experiment '7_8'

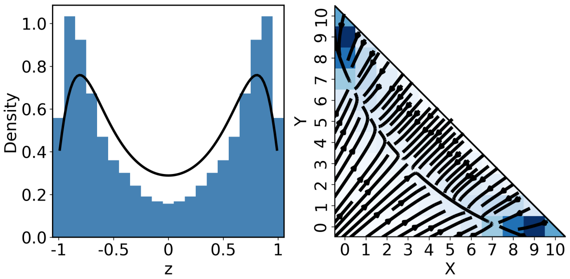

(c) Experiment '10_1'

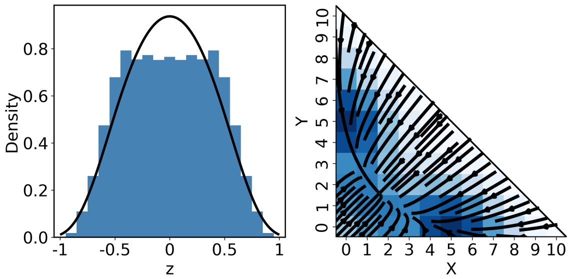

(d) Experiment '10_31'

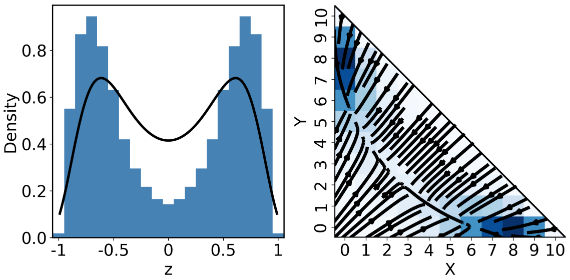

(e) Experiment '10_40'

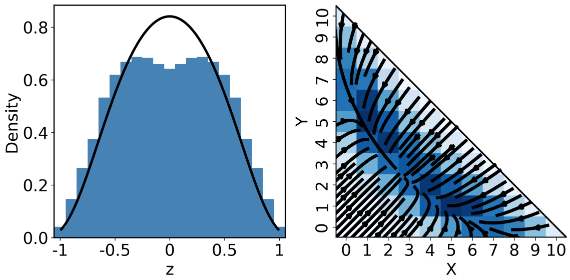

(f) Experiment '10_9'

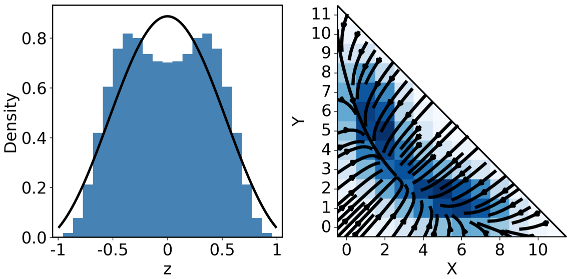

(g) Experiment '11_18'

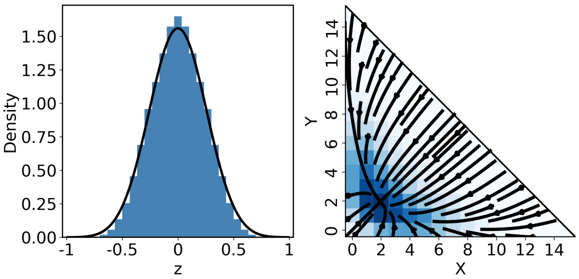

(h) Experiment '15_13'

Figure SI.10: (a-h) (Right) Heat maps of the occupied system states for different experiments with locusts. Plotted over the heatmaps are the flow fields obtained from our model for individually fitted parameters (see Table SI.2 for parameter values). In all cases, the flow field captures the position and curvature of the slow manifold observed in the data. (Left) The projection of the two-dimensional stationary probability distributions (right side) into $z = (X - Y)/N$. The black curve over the histograms is the analytical SPD for z obtained from the curved manifold approximation (see Eq. (SI.1.33)).

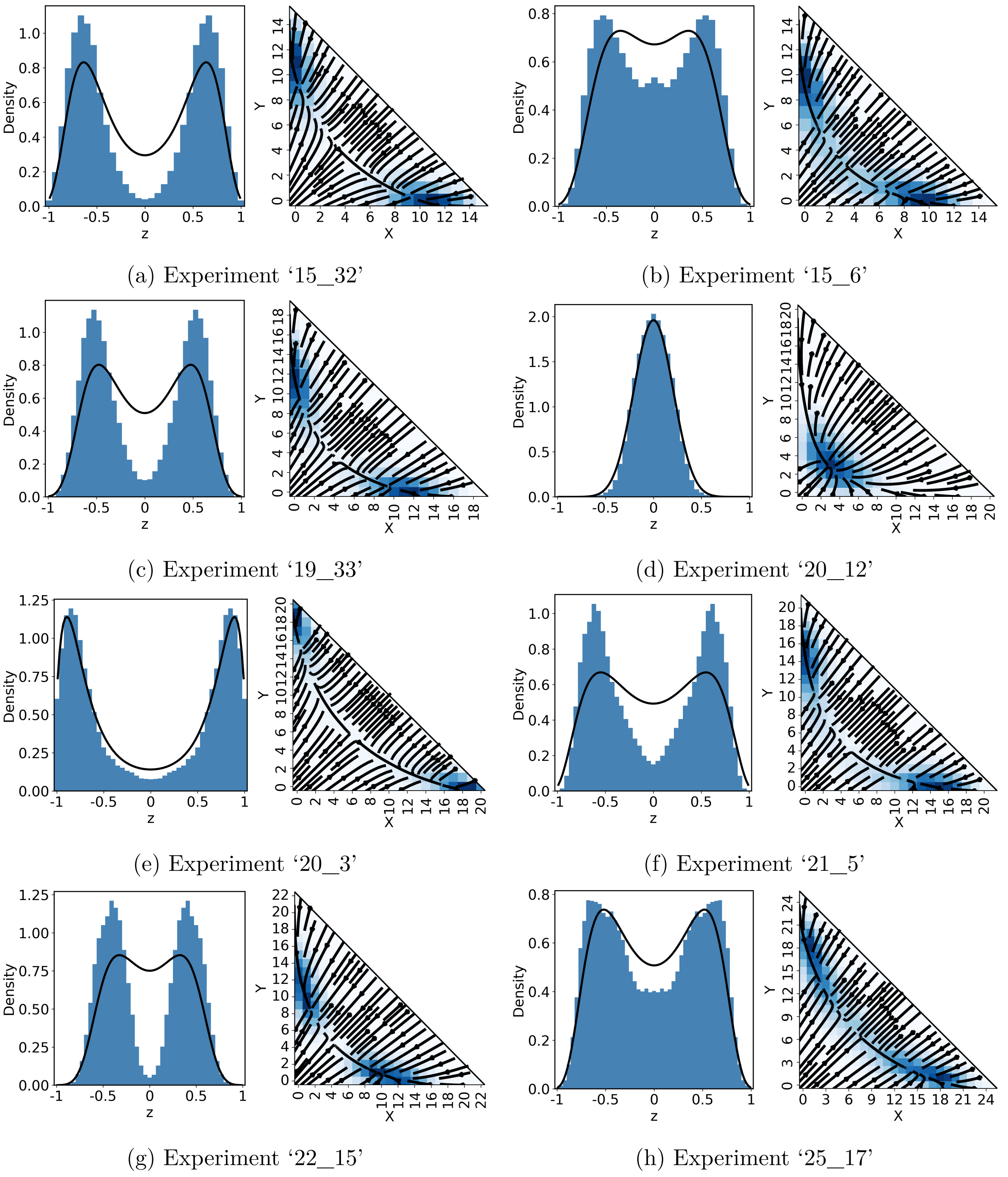

(a) Experiment '15_32'

(b) Experiment '15_6'

(c) Experiment '19_33'

(d) Experiment '20_12'

(e) Experiment '20_3'

(f) Experiment '21_5'

(g) Experiment '22_15'

(h) Experiment '25_17'

Figure SI.11: (a-h) (Right) Heat maps of the occupied system states for different experiments with locusts. Plotted over the heatmaps are the flow fields obtained from our model for individually fitted parameters (see Table SI.2 for parameter values). In all cases, the flow field captures the position and curvature of the slow manifold observed in the data. (Left) The projection of the two-dimensional stationary probability distributions (right side) into $z = (X - Y)/N$. The black curve over the histograms is the analytical SPD for z obtained from the curved manifold approximation (see Eq. (SI.1.33)).

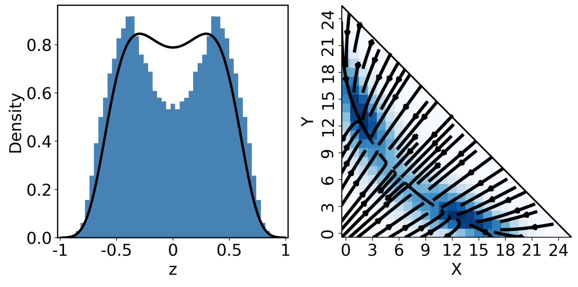

(a) Experiment '25_20'

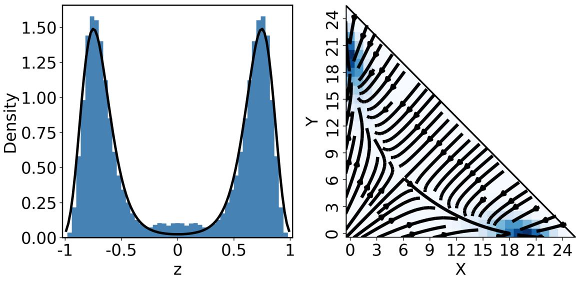

(b) Experiment '25_34'

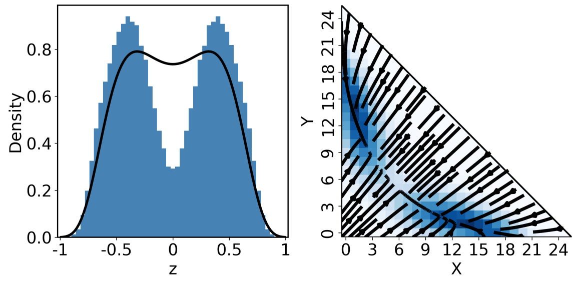

(c) Experiment '25_4'

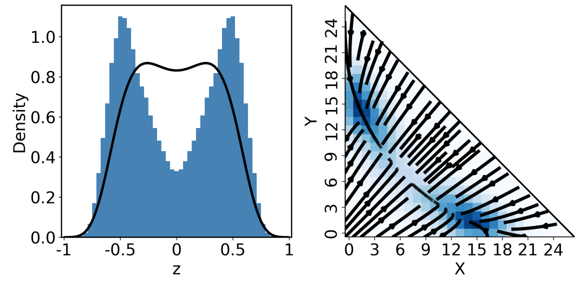

(d) Experiment '26_10'

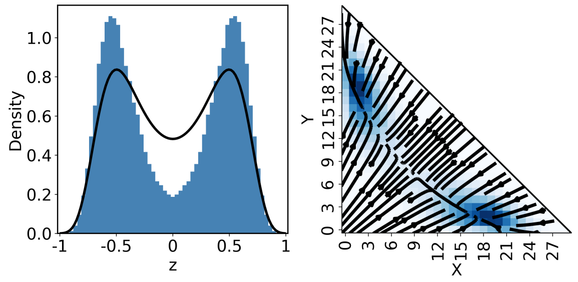

(e) Experiment '29_22'

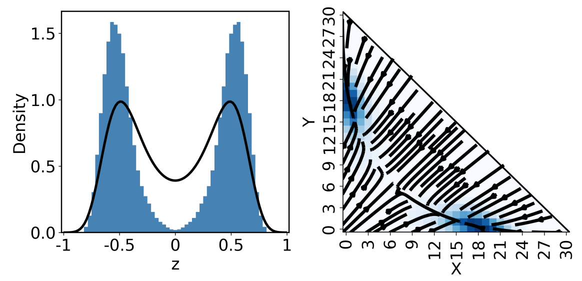

(f) Experiment '30_11'

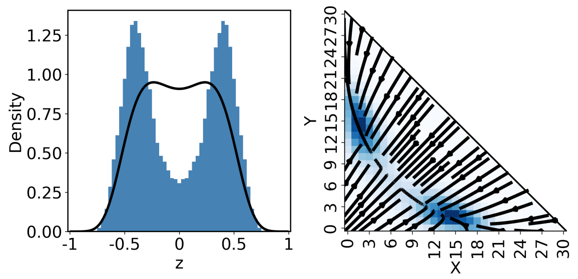

(g) Experiment '30_21'

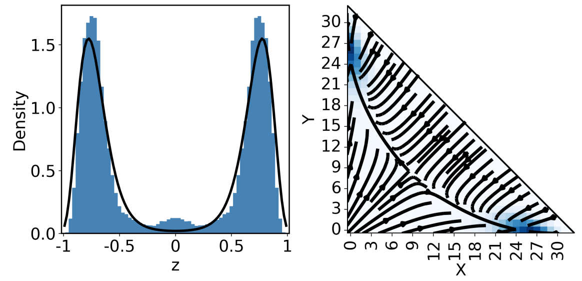

(h) Experiment '32_50'

Figure SI.12: (a-h) (Right) Heat maps of the occupied system states for different experiments with locusts. Plotted over the heatmaps are the flow fields obtained from our model for individually fitted parameters (see Table SI.2 for parameter values). In all cases, the flow field captures the position and curvature of the slow manifold observed in the data. (Left) The projection of the two-dimensional stationary probability distributions (right side) into $z = (X - Y)/N$. The black curve over the histograms is the analytical SPD for z obtained from the curved manifold approximation (see Eq. (SI.1.33)).

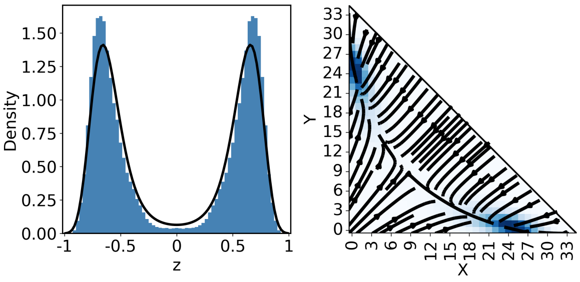

(a) Experiment ‘34_23’

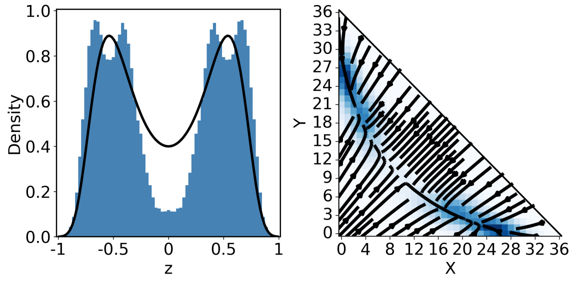

(b) Experiment ‘36_24’

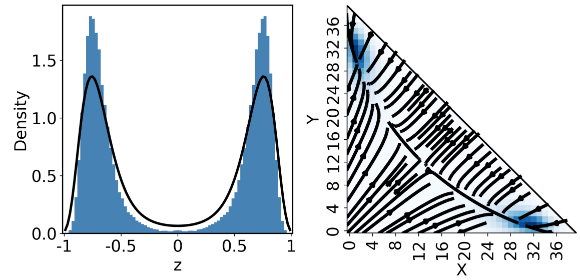

(c) Experiment ‘39_48’

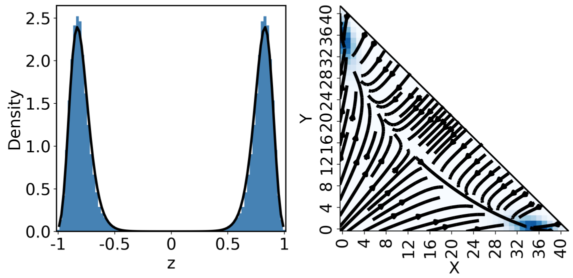

(d) Experiment ‘41_37’

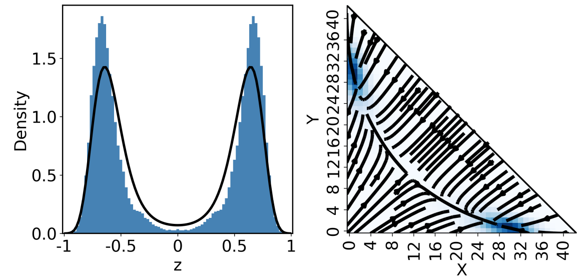

(e) Experiment ‘42_7’

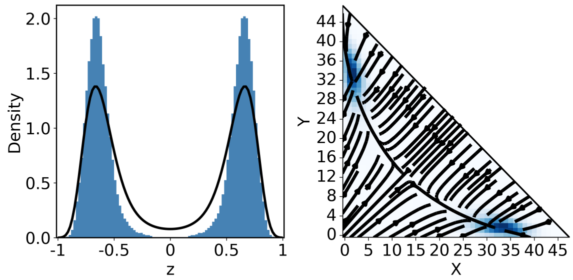

(f) Experiment ‘47_25’

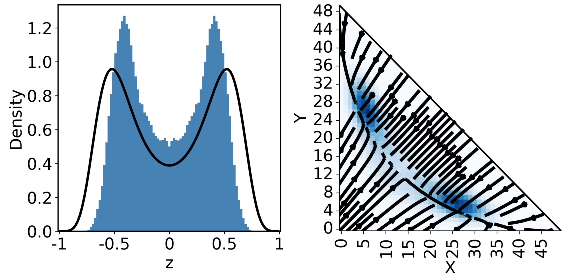

(g) Experiment ‘49_16’

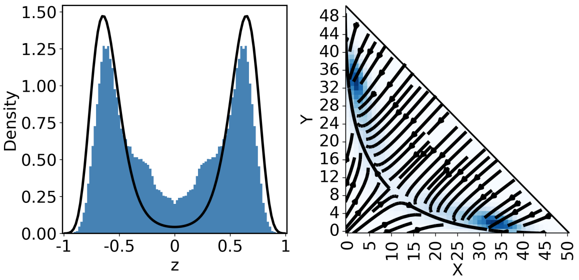

(h) Experiment ‘50_38’

Figure SI.13: (a-h) (Right) Heat maps of the occupied system states for different experiments with locusts. Plotted over the heatmaps are the flow fields obtained from our model for individually fitted parameters (see Table SI.2 for parameter values). In all cases, the flow field captures the position and curvature of the slow manifold observed in the data. (Left) The projection of the two-dimensional stationary probability distributions (right side) into $z = (X - Y)/N$. The black curve over the histograms is the analytical SPD for z obtained from the curved manifold approximation (see Eq. (SI.1.33)).

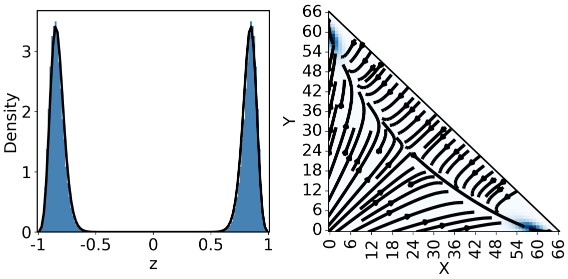

(a) Experiment ‘66_26’

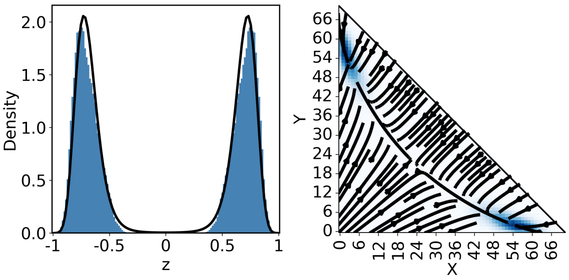

(b) Experiment ‘70_49’

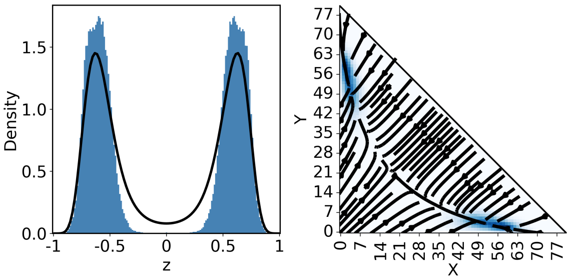

(c) Experiment ‘80_43’

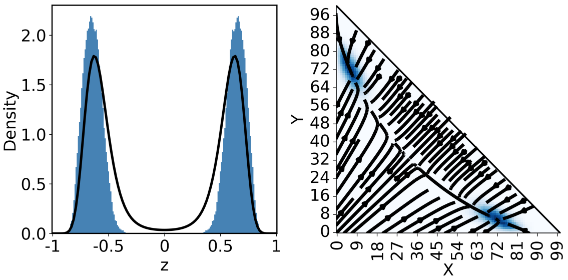

(d) Experiment ‘100_0’

Figure SI.14: (a-h) (Right) Heat maps of the occupied system states for different experiments with locusts. Plotted over the heatmaps are the flow fields obtained from our model for individually fitted parameters (see Table SI.2 for parameter values). In all cases, the flow field captures the position and curvature of the slow manifold observed in the data. (Left) The projection of the two-dimensional stationary probability distributions (right side) into $z = (X - Y)/N$. The black curve over the histograms is the analytical SPD for z obtained from the curved manifold approximation (see Eq. (SI.1.33)).

SI.5 Iterated human voting game

We designed and performed experiments with human participants to test our theoretical predictions in a new data set and obtain insight into collective decision-making in human groups, particularly on the role of neutral decisions.

We performed 12 experiments with the option to abstain. The experiments had group sizes 12, 13, 16, 18, 19, 19, 20, 22, 25, 28, 31 and 33, resulting in 256 participants. We performed an additional 8 experiments without the option to abstain with group sizes 13, 14, 15, 15, 15, 18, 18, and 20 resulting in 128 participants. In both cases, participants were screened on Prolific [Prolific, 2014], considering any English-speaking adult resident in the UK.

Recruitment. Recruitment of participants was achieved using the Prolific platform. Recruitment and experiment occurred in separate stages, due to the limitations of the platform for multiplayer games. One hour before the scheduled time for the experiment, a recruitment ‘study’ was set up on Prolific. The recruitment ‘study’ consisted of an app developed on oTree [Chen et al., 2016] containing basic information about the experiment, the participant

information sheet, and the consent form. Fifteen minutes before the experiment, we would invite participants who had consented to take part in the experiment, informing them the experiment would start at the scheduled time and explaining that they would be invited to the experiment 'study' containing the link to the experiment app. The experiment 'study' was set up on Prolific 5–10 minutes before the start of the experiment, linking to the virtual room of the experiment. Only people who had consented were invited to this 'study'. The experiment started once enough people had joined the experiment room. We took precautions which ensured participants were prevented from joining multiple experiments.

Experiment design. A group of between 12 and 33 participants is formed by including all participants who consent to participate in the experiment and join the virtual experiment room at the agreed time. Participants are paid a fixed fee for participation and receive a bonus payment equal to the participation fee if they are in the top 50% scoring participants. The participants play 120 rounds of a voting game. In each round, the participants have 10 seconds to choose Option X, Option Y, or Abstain. Failing to make a decision in time results in an abstention. Voting (i.e., choosing option X or option Y) costs 100 points, while abstaining does not deduct points. Participants who vote with the majority in the round are given 150 points. This information is presented to the participants before the start of the game as in Fig. SI.15. A majority is defined as the voting option with the most votes. In case of a tie, no one receives points. At each round after the first, participants are presented with information about the previous round. This information consists of a sample of 9 votes randomly generated for each participant. The sample is formed by randomly drawing 9 votes from the previous round. Noise is then introduced in the sample to mimic the effects of imperfect information. We introduce noise in the sample by allowing each of the 9 drawn votes to be modified with probability 0.4. If a drawn vote is modified in the sample, it is substituted by a uniformly drawn decision between Option X, Option Y or Abstain. We note that modifications occur only in the sample shown to participants and not in the recorded votes.

Initialisation. In order to observe a departure from a consensus system state, we start from the position of showing individuals' samples as if they were in a consensus system state with only Y votes, introducing noise for each participant's sample as explained. Participants are still rewarded based on their cast decisions in the first round.

Experiment protocol. The structure of the experiments was the following: (i) Participants are presented with the participant information screen and asked to confirm their consent. (ii) Participants who consented are grouped for the experiment. (iii) They are led to a screen with the rules of the game. (iv) They play 5 "training" rounds to adapt to the functionality of the experiment. (v) They are shown the results of the training session, including their ranking. These are not considered for the results and payments. (vi) The experiment starts. They play 120 rounds of the voting game. (vii) At the end of the experiment they are presented

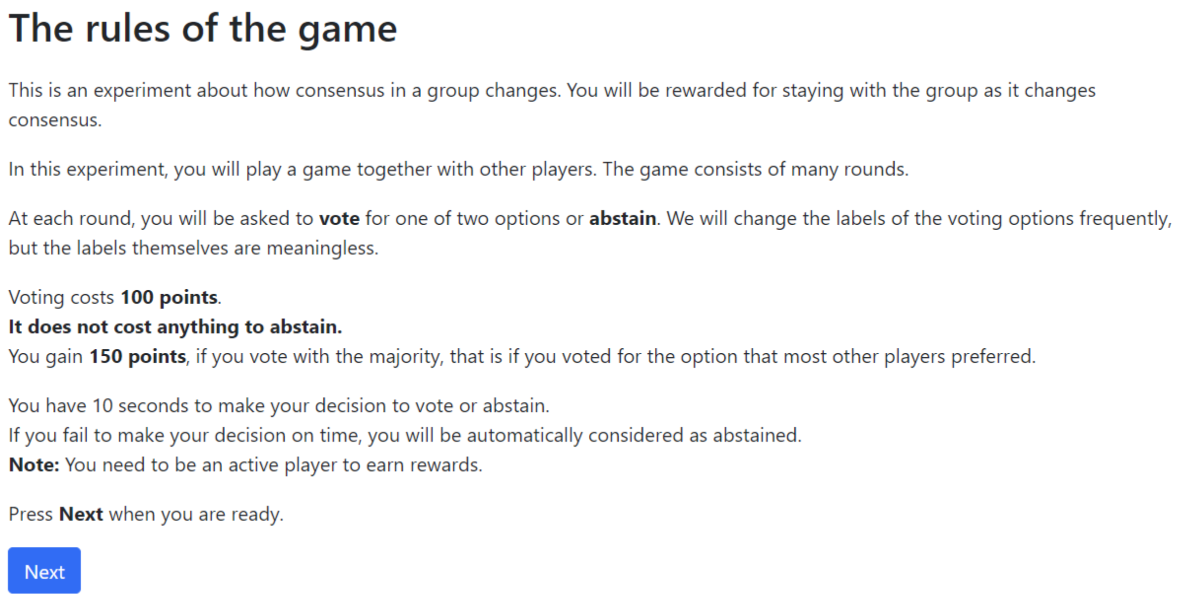

Figure SI.15: The initial screen presented to the participants in the voting game experiment with abstentions.

their ranking. They are then presented with the debrief screen and asked optionally to describe their strategy. (viii) They are then redirected to Prolific with the corresponding code (completed, bonus payment, failed attention checks). Those are then used to make the appropriate payments.

Labels and position randomisation. At every round, we randomise the label for options X and Y by different geometrical shapes (square, parallelogram, left right-triangle, right right-triangle, rhombus, pentagon and hexagon) to reduce the effect of preferences and randomise the position of the buttons between X and Y keeping the Abstain button in its place to reduce memory effects. We also randomise the colour of the Abstain and vote buttons between participants, fixing them throughout the experiment. Fig. SI.16 depicts a participant's experiment screen in two consecutive rounds.

Dropouts. Participants who fail to click a vote or abstain button for 5 consecutive rounds are presented with an activity check screen, in which they have 10 seconds to click a button confirming that they are active. Failing activity checks twice results in a dropout. Dropouts are substituted by bots to keep the flow of the game. Bots will always vote for the option with a lead of 2 or greater in the shown sample and abstain if the difference in votes for option X and option Y is 1 or less. No experiment had 2 or more dropouts, guaranteeing dropouts did not substantially affect the results.

Experiments without abstentions. We also performed experiments in which the option to abstain was removed. In this case, failing to vote in time had the same cost as voting. In this new setup, we draw 9 votes from the previous round, changing each with a probability

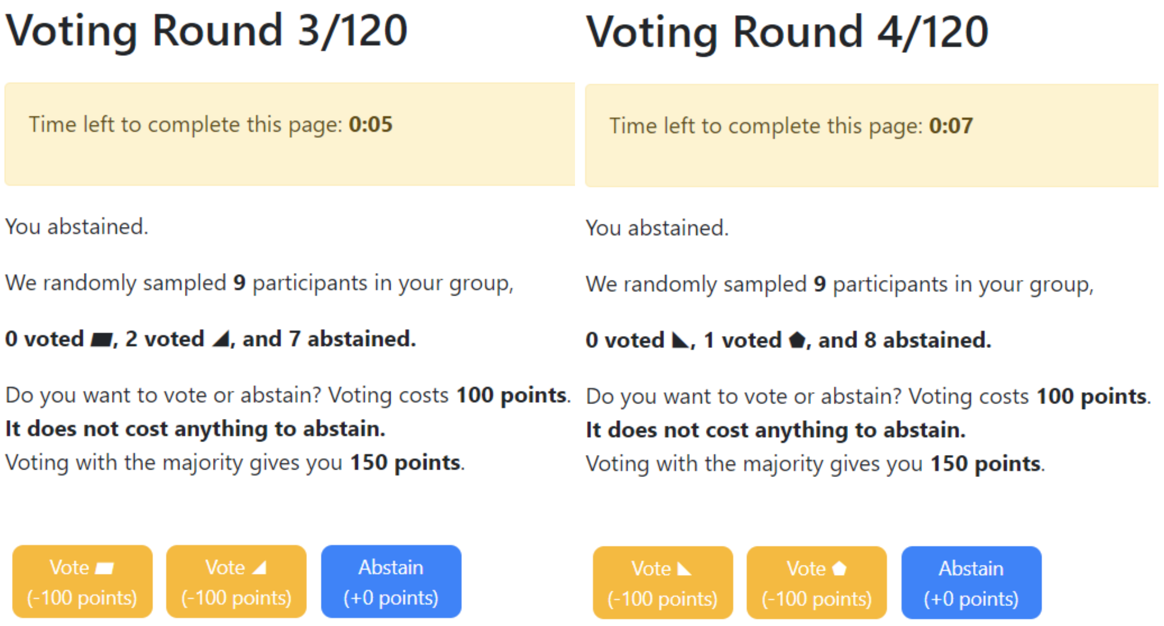

Figure SI.16: The screen the participants see at each round showing if they voted with the majority or abstained and the personalised samples from the previous round. The labels of options X and Y change each round, and their position is changed with probability $1/2$, but the Abstain box is kept constant.

0.2667 and drawing modified votes uniformly between X and Y, such that the mean number of noisy votes for X and Y is the same as in the experiments with abstentions. The rules of the game and the voting page are shown in Fig. SI.17. Any reference to voting costs was removed, as there is no cost difference between voting for X, Y, or failing to vote. The reward shown, 50 points, is the effective net difference in points for voting with the majority compared to voting for the minority in the original experiment. The few instances of failing to vote in time during the experiments without abstentions did not substantially impact the results. There were no dropouts in any of the three experiments.

SI.5.1 Calibration experiments

We designed an experiment to obtain the empirical distribution of the probability of voting for each option X, Y or choosing to Abstain based on the sample shown to participants. We used the same experiment and protocol as described above, however, at each round, rather than showing a sample of the votes of the group in the previous round, we showed the same predetermined samples to all participants and registered their votes. The predetermined samples shown were prepared to cover all the state space of a particular sample size as many times as possible in the 120 rounds of the experiment. We decided to test both a sample of 5 votes and a sample of 9 votes.

For the sample of 5 votes, there are 12 unique combinations of distributions into three stances:

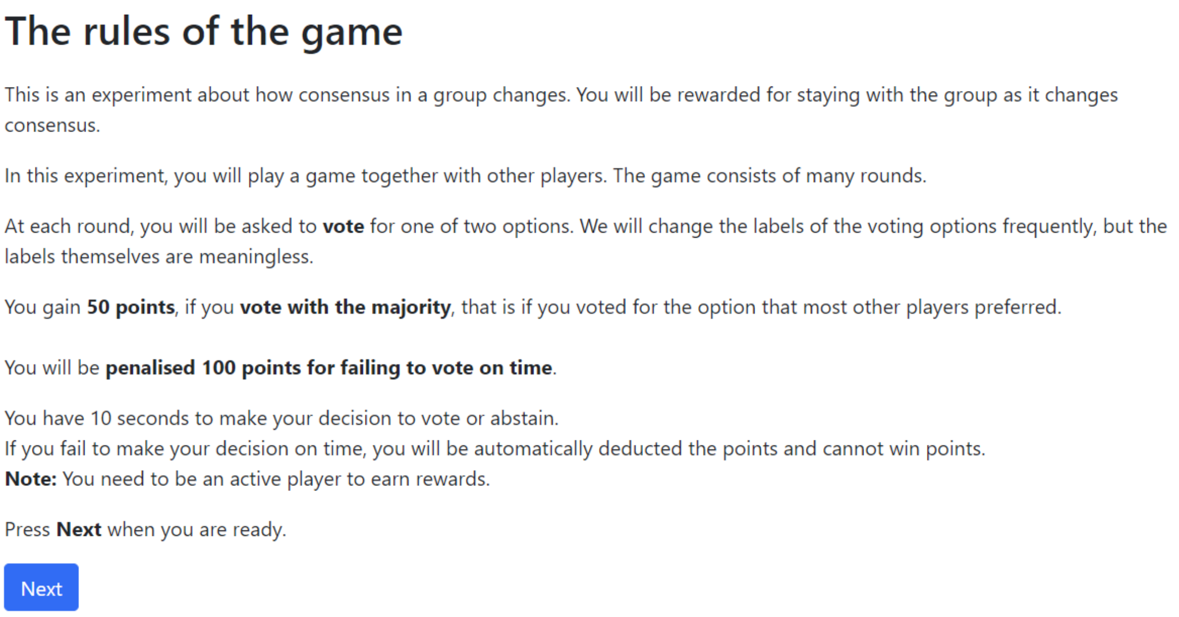

Figure SI.17: The screen presented to the participants in the voting game experiment without abstentions.

5,0,0; 4,0,1; 4,1,0; 3,0,2; 3,1,1; 3,2,0; 2,0,3; 2,1,2; 2,2,1; 1,0,4; 1,1,3; 0,0,5. We then repeated each of these 10 times to make the 120 rounds. For samples that have different numbers of X and Y, we showed half of them with X majority and half with Y majority (e.g., 5,0,0 and 0,5,0 five times each to make the 10 rounds of the 5,0,0 combination). After the 120 rounds were listed, we randomised the order to avoid any obvious pattern in sequential rounds. The same approach has been applied to a sample of 9 votes. In this case, there are 30 unique combinations (not listed here), each shown 4 times in the experiment. Bonus payments were determined as before by rewarding participants based on their vote and the majority vote, regardless of the samples shown.

We performed one experiment with a sample of 5 votes with 7 participants, providing 70 data points to estimate the voting distribution for each unique sample. Another experiment with a sample of 9 votes had 11 participants, providing each unique sample with 44 data points to estimate the voting distribution between stances X, Y and Abstain. Fig. SI.18 shows the empirical distributions in both cases.

Using the above two empirical distributions, we ran simulations of the experiment to determine the optimal experimental parameters to increase the probability of observing a switch in consensus within the 120 rounds of the experiments. We then determined the optimal group size, the probability of modifying a sampled vote, p, and whether it was optimal to show 5 or 9 votes to the participants. A range of parameters consistently gave at least 40% chance of observing a switch in which the system would stay at least 20 rounds in sequence in one option and 20 rounds in sequence in the other option. For the sample of 5 votes, the optimal

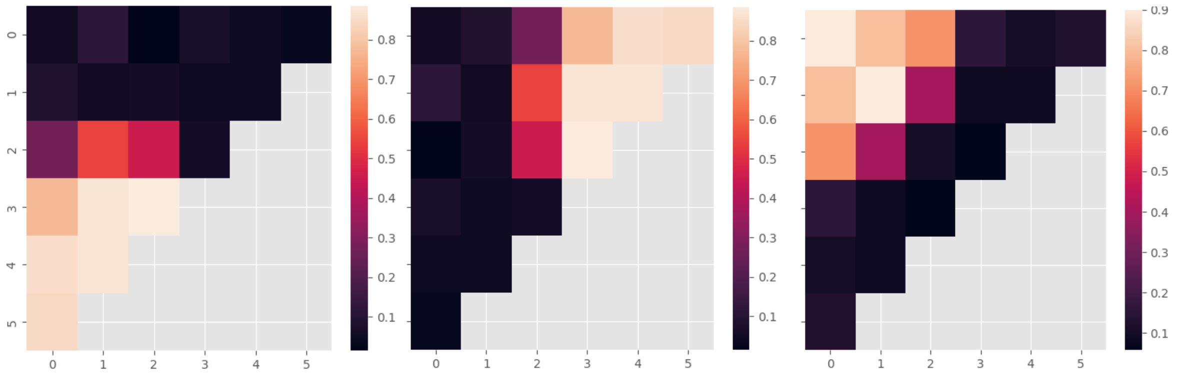

(a) Probability of voting X. (b) Probability of voting Y. (c) Probability of abstaining.

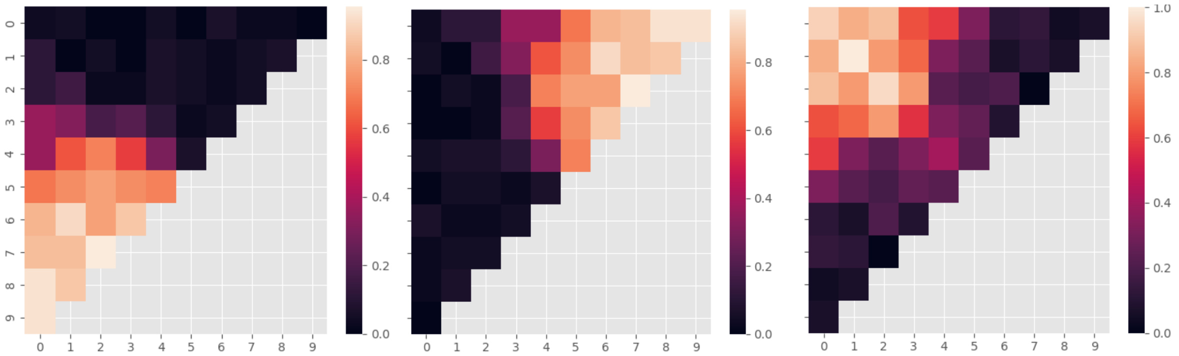

(d) Probability of voting X. (e) Probability of voting Y. (f) Probability of abstaining.

Figure SI.18: Empirical distributions for the sample of 5 votes (top row) and 9 votes (bottom row). The number of Y votes and X votes in the sample shown to participants are represented in the horizontal and vertical axes, respectively. We symmetrise X and Y data to obtain more data points for estimating the probabilities. As expected, the participants vote for the option with the most votes in the sample, with a probability that increases with the number of votes for the majority option. The participants abstain more when there is not a clear preference for any of the options.

probability, p, of changing each vote in the sample was in the interval 0.2-0.25, with group sizes of 15 to 25 participants and sampling X, Y, or Abstaining from a uniform distribution. For the sample of 9, the optimal p ranged between 0.35-0.45 with group sizes of around 15 to 20 participants and uniform sampling. The best simulation results were obtained with $p = 0.4$, 15 to 20 participants, and a sample of 9, which were used in the experiments as described in the previous section.

SI.6 Voting experiments analysis

SI.6.1 Curvature

For each experiment, let N be the experiment's group size. For each round of the experiment, let X be the number of votes for the first option, Y the number of votes for the second option,

and W the number of abstentions. Let $x = X/N$ and $y = Y/N$.

To reduce the dimensionality of the data, we project it along the two 'fast flows' obtained from the manifolds to check which describes the data best. Our theory predicts two manifolds. The first is a straight-line manifold described by $1 - x - y = \alpha$, where α is a parameter. The corresponding fast-flow projection is the lines in which x/y is constant. The second is a hyperbolic-manifold described by $1 - x - y - \gamma xy = 0$. The fast-flow projections in this case are the lines of constant $x - y$.

We first test the projection along the fast flow of the straight-line manifold. From the original data (X, Y), we obtain a new data set, with variables $k = (x - y)/(x + y)$ and $w = W/N$, where W is the number of abstentions. Note that k can vary from -1 to 1 and w from 0 to 1.

To group the data, we discretise k into bins. Then, we compute the average density of abstentions, w, for each particular bin and the standard deviation, σ, of the data in that bin. The error of the mean of a bin is then $\epsilon = \frac{\sigma}{\sqrt{n}}$, where n is the number of data points in the bin.

Now that we have 1-dimensional data and associated errors, we can symmetrise it and use it to fit our models. We will test 3 models: the straight-line, given by the curve $1-x-y = \alpha$; the hyperbola, given by $1-x-y-\gamma xy = 0$; and the combined curve, given by $1-x-y-\gamma xy = \beta$. Note that, $\beta = 0, \gamma \neq 0$ corresponds to the curved manifold, while $\beta \neq 0, \gamma = 0$ corresponds to the straight line.

Fitting the data (k, w) to the models, we get the following values for the parameters and sum of normalised squared residuals, σ_f^2:

1. Line: $\alpha = 0.144 \pm 0.005$. $\sigma_f^2 = 441.59$.
2. Hyperbola: $\gamma = 1.021 \pm 0.025$. $\sigma_f^2 = 136.34$.
3. Combination: $\gamma = 0.89 \pm 0.10$, $\beta = 0.020 \pm 0.014$. $\sigma_f^2 = 131.34$.

These suggest that the hyperbola is the best fit between the straight and the curved manifold, but the third option that combines both has a lower error. This is unsurprising since the combined fit has an extra degree of freedom, however, it confirms the curvature of the lower dimensional manifold. Fig. SI.19 shows the results of the fitting and the residuals of the fitted curves. The residuals for the straight-line fit show a clear "curved" pattern, reinforcing that the line fit is not appropriate for the data. The residuals for the hyperbolic fit do not present any clear patterns with fluctuations around the fitted curve.

Performing the z-test on the parameters of the mixed fit, we get $z_\gamma \approx 8.9$, while $z_\beta \approx 1.43$, which is within the 95% confidence interval of zero (right-tailed test). Hence, from both model selection (patterns in residuals and sum of squared residuals) and hypothesis testing

on the fitting of both parameters, we can conclude that there is a curvature in the data. Consequently, the switching between the two consensus system states occurs through an increase in the number of abstentions.

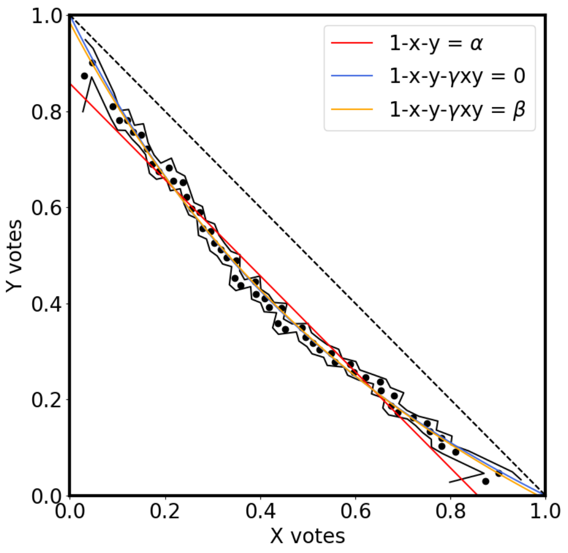

(a) Fitted curve for the $k = \frac{X-Y}{X+Y}$ projection.

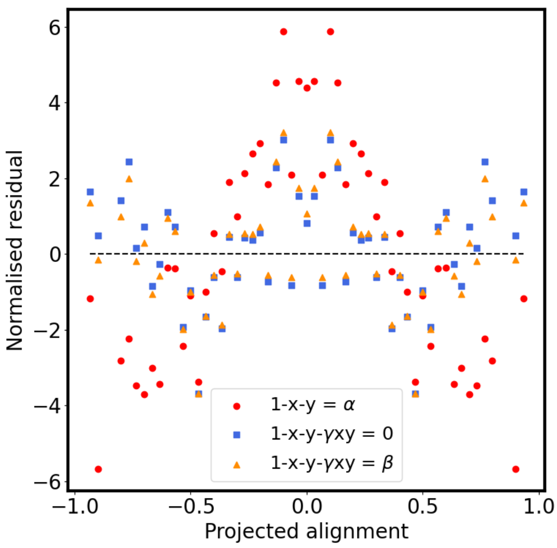

(b) Normalised residuals for the fitted curves.

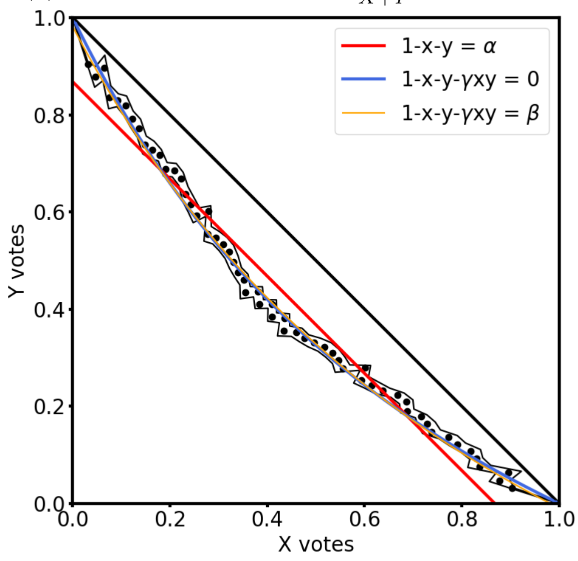

(c) Fitted curve for the $k = \frac{X-Y}{N}$ projection.

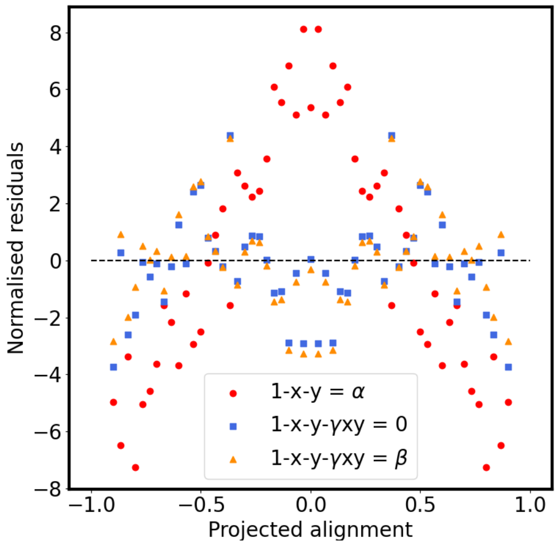

(d) Normalised residuals for the fitted curves.

Figure SI.19: Human voting data strongly favours a curved manifold over a straight one, validating that consensus changes proceed through states of increased system neutrality rather than constant neutrality. (a)-(c) Fitted curves to the aggregated projected data following the straight (a) and hyperbolic (c) manifold projections. In both cases, the continuous lines give the interval containing 95% of the data. (b)-(d) Corresponding normalised residuals of the fitted curves.

We now do the same analysis but take the second projection: $x - y$ = constant. From the original data, we define $z = (X - Y)/N$, where N is the group size for the particular

experiment, and $w = W/N$. Then, we discretise the values of z into bins and add the data point w to the corresponding bin of z. Finally, we symmetrise the data taking the average of each bin and get the standard deviation of the data of each bin, which we divide by the square root of the number of points in the bin to get the error of the average. We then fit the line $1 - x - y = \alpha$, the hyperbola (curved manifold, $1 - x - y - \gamma xy = 0$) and the combined fit ($1 - x - y - \gamma xy = \beta$) as before.

Fitting the data (z, w) to the models, we get the following values for the parameters and sum of squared normalised residuals, σ_f^2:

1. Line: $\alpha = 0.137 \pm 0.006$. $\sigma_f^2 = 800.42$.
2. Hyperbola: $\gamma = 1.062 \pm 0.027$. $\sigma_f^2 = 167.69$.
3. Combination: $\gamma = 0.92 \pm 0.09$, $\beta = 0.020 \pm 0.012$. $\sigma_f^2 = 159.58$.

These results suggest that the hyperbolic manifold is a better fit for the data than the line fit. Additionally, analysis of the residuals shows a clear curved pattern in the residuals of the line fit (see Fig. SI.19). As for the curved fits, there seems to be a less clear pattern.

The Bayesian Information Criterion (BIC) [Stoica and Selen, 2004] can be used for model selection. It is defined as

$$\text{BIC} = n_d \log(n_s) + n_s \log\left(\sigma_f^2\right), \tag{SI.6.1}$$

for $n_s = 57$, the number of data points used in the fit, and n_d the number of parameters of the fitted model.

For the hyperbolic fit, the BIC is 296.00, while for the combined fit, we obtain 297.22 as it has an extra parameter that does not significantly improve the reduced squared error when compared to the hyperbolic fit. For the linear fit, the BIC is 385.10. Hence, the hyperbolic model should be selected. We can then conclude that the switching in consensus occurs via a low active system state of higher numbers of abstentions.

SI.6.2 Discrete model and path likelihood definitions

To use the voting data to fit parameters to our general model presented in Section SI.1, we need to conciliate the discreteness in time of our data (played in rounds) to the continuous model. To overcome this issue, we present a method based on the likelihood of the path (or time series) of the experiments. The method consists of defining a likelihood function from the data on each interval or round conditioning on the state of the system at the previous step [Dai et al., 2020, Zheng and Sun, 2017, Zheng et al., 2020].

Recall our general continuous model:

$$\mathcal{A} \to \mathcal{B}: \quad q_{ab} = a_{\mathcal{AB}} + b_{\mathcal{AB}}\frac{n_{\mathcal{B}}}{N} + c_{\mathcal{AB}}\frac{n_{\mathcal{C}}}{N},$$

for distinct $\mathcal{A}, \mathcal{B}, \mathcal{C} \in \{X, Y, W\}$.

Let v_t be the vote of a particular individual in round t and $p_t^{\mathcal{A}|\mathcal{B}} = \mathbb{P}(v_{t+1} = \mathcal{A} \mid v_t = \mathcal{B}, X(t) = n_X, Y(t) = n_Y, W(t) = n_W)$, for $\mathcal{A}, \mathcal{B} \in \{X, Y, W\}$. Then we can define the state vector $P_t = \left(p_t^X, p_t^Y, p_t^W\right)$ which gives the probability that the participant has, after one round, stance X, Y, or W, respectively. The description of the discrete-time experiment from our continuous-time model comes from mapping to a discrete-time Markov chain [Levin and Peres, 2017, Grimmett and Stirzaker, 2020]. The equations that govern the evolution of the discrete-time variable P_t are then,

$$P_{t+1} = P_t T_t, \quad T_t = e^{Q(t)},$$
$$Q(n_X, n_Y, n_W; t) = \begin{pmatrix} -q_{XY} - q_{XW} & q_{XY} & q_{XW} \\ q_{YX} & -q_{YX} - q_{YW} & q_{YW} \\ q_{WX} & q_{WY} & -q_{WX} - q_{WY}. \end{pmatrix},$$

where P_0 is the stance of the individual in the previous round, and q_{ij} are the total rates, which depend on n_X, n_Y and n_W.

By definition,

$$T_t \equiv \begin{pmatrix} p_t^{X|X} & p_t^{Y|X} & p_t^{W|X} \\ p_t^{X|Y} & p_t^{Y|Y} & p_t^{W|Y} \\ p_t^{X|W} & p_t^{Y|W} & p_t^{W|W} \end{pmatrix}.$$

Now, rather than estimating T from the data, we can define a likelihood as follows.

For a participant i, let $V_t^{(i)}$ be the random variable corresponding to their vote at time t and let the data points $\mathbf{v}^{(i)} = \left(v_1^{(i)}, v_2^{(i)}, \ldots, v_n^{(i)}\right)$ be the record of the votes of this participant at each round. Then, using the Markov property,

$$\mathbb{P}\left(V_1^{(i)} = v_1^{(i)}, \ldots, V_n^{(i)} = v_n^{(i)}\right) = \mathbb{P}\left(V_n^{(i)} = v_n^{(i)} \mid V_{n-1}^{(i)} = v_{n-1}^{(i)}\right) \times \cdots \times \mathbb{P}\left(V_1^{(i)} = v_1^{(i)}\right)$$

But $\mathbb{P}(V_n = v_n \mid V_{n-1} = v_{n-1})$ are the transition probabilities, the entries of the matrix $T_t = e^{Q(t)}$. Hence, we can define a likelihood function

$$L\left(\theta \mid \left\{\mathbf{v}^{(i)}\right\}_{i \in \mathcal{P}}\right) = \prod_{i \in \mathcal{P}} \prod_{t=1}^{120} \mathbb{P}\left(V_t^{(i)} = v_t^{(i)} \mid V_{t-1}^{(i)} = v_{t-1}^{(i)}; \theta\right), \tag{SI.6.2}$$

where $\mathcal{P}$ is the set of participants in the experiment and θ the parameters. The product over $\mathcal{P}$ comes from the fact that each participant makes their decision independently, hence, the steps of each trajectory are independent when conditioned on the natural filtration. For a given set of parameters, θ, its likelihood is easy to compute: for each round, t, we know n_X, n_Y and n_W, meaning we can compute the matrix $Q(t)$ and exponentiate to obtain the matrix T_t. Then, we only need to multiply, for each participant, the entry of the matrix T_t

corresponding to the observed transition.

SI.6.3 Fitting general model parameters to voting experiments data

The discreteness in time of the data in rounds limits the available methods for fitting our model parameters. The most successful method consisted of assuming the curved manifold approximation of Section SI.1.5 and fitting the analytical SPD of Eq. (SI.1.33) to the empirical SPD by minimising the squared error using the particle swarm algorithm [Kennedy and Eberhart, 1995, Kennedy, 2006, Bonyadi and Michalewicz, 2017] implemented in MATLAB for the minimisation problem. Fitting the SPD provides us all parameters besides the timescale of the dynamics, which can be obtained by maximising the likelihood defined by Eq. (SI.6.2) using time series data. Table SI.3 shows the parameter values obtained from the fitting. Figs. SI.20 to SI.31 depict the data overlaid with the fitted fast flow and analytical stationary probability distributions derived from the hyperbolic manifold approximation.

Experiment	a_{XY}	b_{XY}	c_{XY}	a_{XW}	b_{XW}	c_{XW}	a_{WX}	b_{WX}	c_{WX}
12_1	0.0000	0.5277	0.0009	0.56	3.18	2.35	3.21	1.95	0.0297
13_1	0.0143	0.0016	0.0305	0.92	0.39	2.59	1.63	5.61	0.0010
16_1	0.0003	0.0000	0.0077	1.80	0.07	9.98	8.54	9.05	0.0023
18_1	0.0141	0.0019	0.0152	0.53	0.75	3.09	1.99	3.69	0.0005
19_1	0.0058	0.0019	0.0634	1.17	0.02	2.58	2.25	7.61	0.0000
19_2	0.0036	0.0224	0.0034	0.32	0.57	3.05	2.79	4.34	0.0002
20_1	0.0020	0.0019	0.0046	1.25	0.51	2.91	2.23	7.12	0.0016
22_1	0.0042	0.0008	0.0060	0.93	0.11	3.23	2.32	5.27	0.0043
25_1	0.0038	0.0009	0.0041	1.88	0.01	4.81	3.69	4.18	0.0000
28_1	0.0100	0.0342	0.0094	1.96	10.83	8.07	7.11	8.58	0.0131
31_1	0.0031	0.0527	0.0037	1.09	4.28	5.79	4.38	1.40	0.0179
33_1	0.0053	0.0010	0.1481	0.80	0.016	2.00	1.33	4.92	0.0000

Table SI.3: Fitted parameters to each of the voting game experiments.

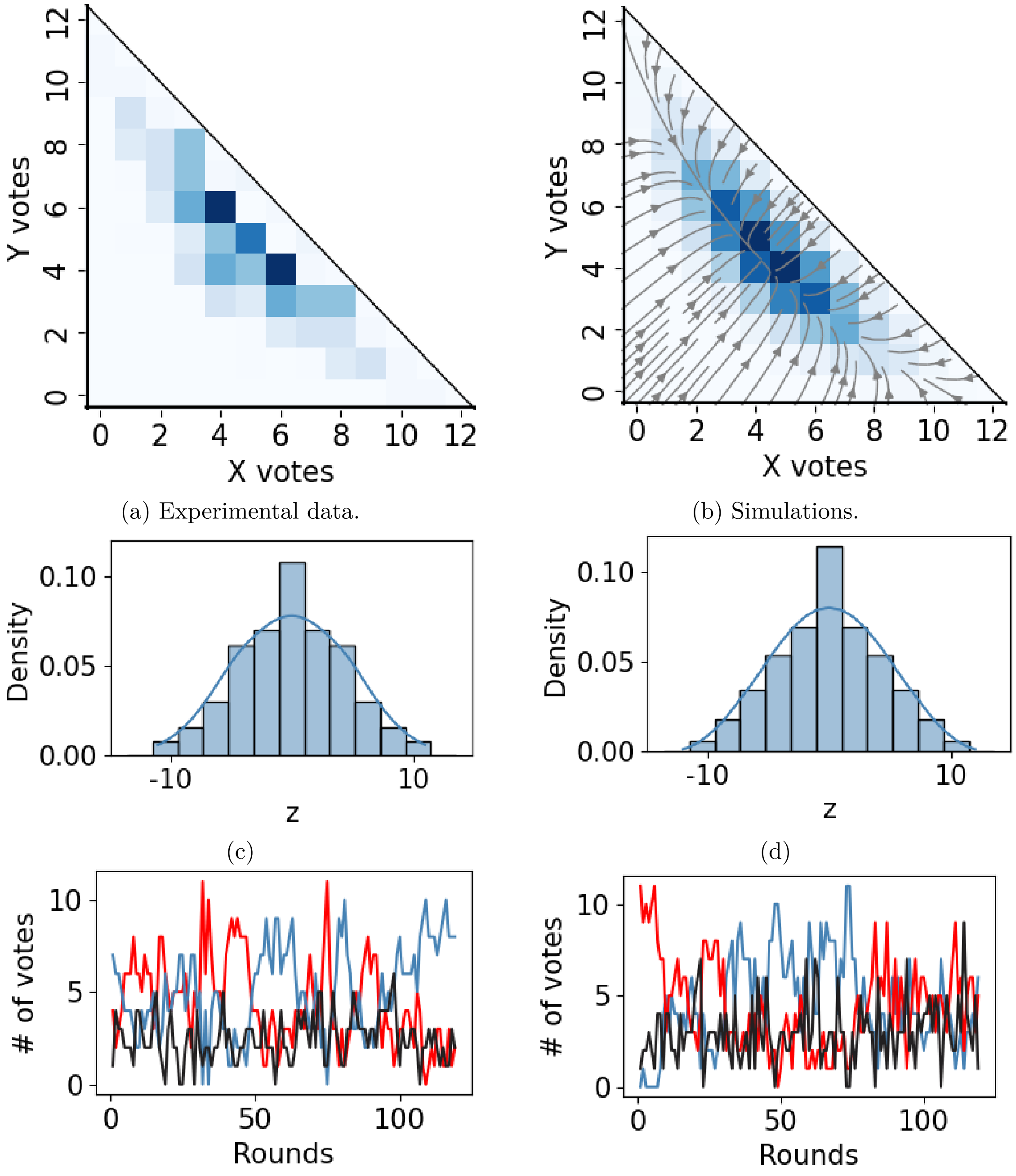

(a) Experimental data.

(b) Simulations.

(c)

(d)

(e)

(f)

Figure SI.20: (a) A heat map of the data of an experiment with $N = 12$ participants. (b) A heat map generated from a simulated time course with the flow field superimposed atop. Parameters for the simulations are fitted from experimental data. (c-d) One-dimensional projections of the stationary probability distributions onto $Z = (X - Y)$ (see SM Sections 1 and 6 for details) for experimental (c) and simulated data (d). The Gaussian kernel density estimation is shown atop the histograms. (e-f) Time courses of the experimental (e) and simulated data (f). Votes for option X, option Y and abstentions are represented in blue, red and black, respectively.

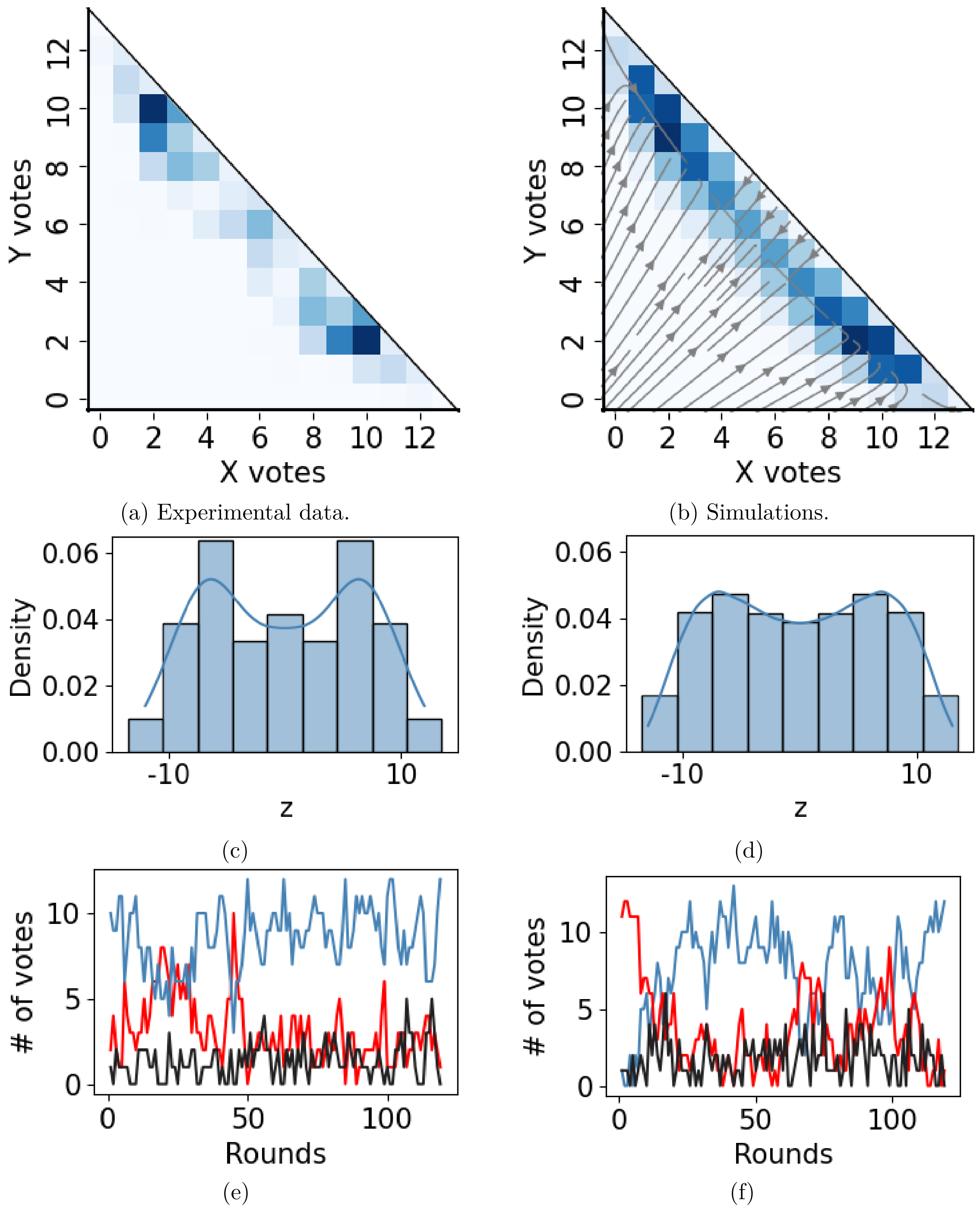

Figure SI.21: (a) A heat map of the data of an experiment with $N = 13$ participants. (b) A heat map generated from a simulated time course with the flow field superimposed atop. Parameters for the simulations are fitted from experimental data. (c-d) One-dimensional projections of the stationary probability distributions onto $Z = (X - Y)$ (see SM Sections 1 and 6 for details) for experimental (c) and simulated data (d). The Gaussian kernel density estimation is shown atop the histograms. (e-f) Time courses of the experimental (e) and simulated data (f). Votes for option X, option Y and abstentions are represented in blue, red and black, respectively.

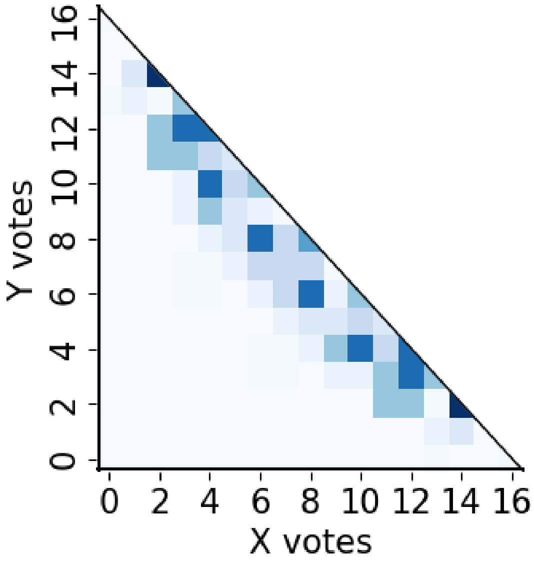

(a) Experimental data.

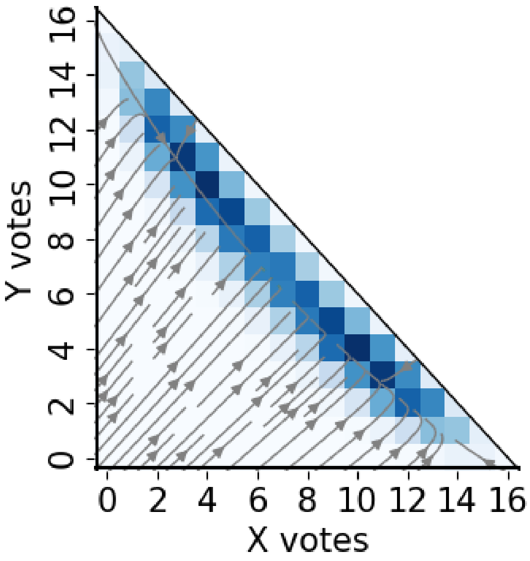

(b) Simulations.

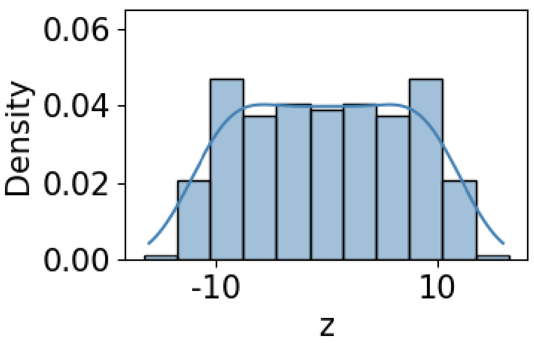

(c)

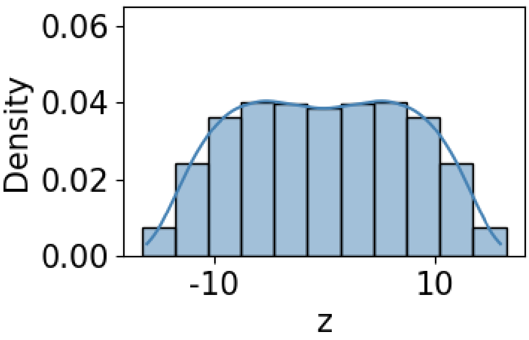

(d)

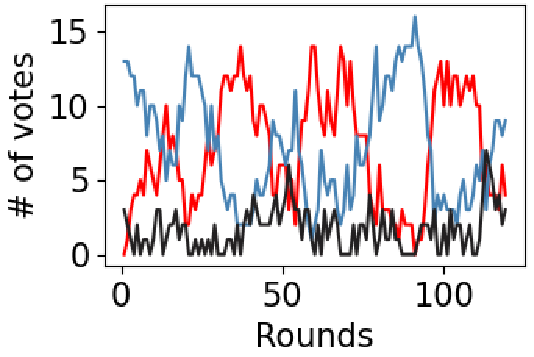

(e)

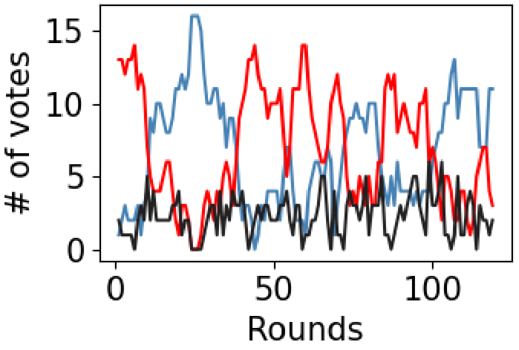

(f)

Figure SI.22: (a) A heat map of the data of an experiment with $N = 16$ participants. (b) A heat map generated from a simulated time course with the flow field superimposed atop. Parameters for the simulations are fitted from experimental data. (c-d) One-dimensional projections of the stationary probability distributions onto $Z = (X - Y)$ (see SM Sections 1 and 6 for details) for experimental (c) and simulated data (d). The Gaussian kernel density estimation is shown atop the histograms. (e-f) Time courses of the experimental (e) and simulated data (f). Votes for option X, option Y and abstentions are represented in blue, red and black, respectively.

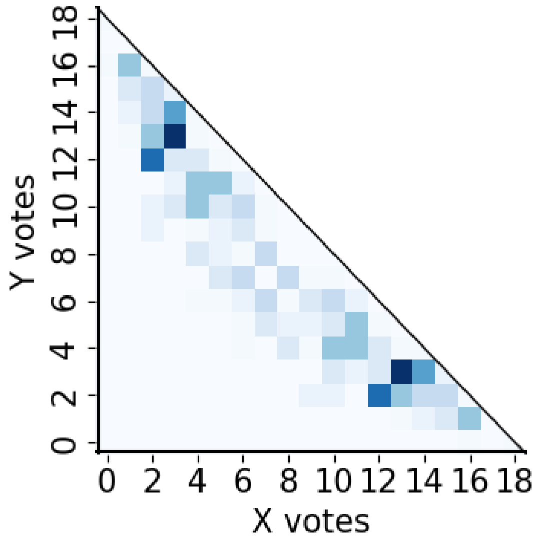

(a) Experimental data.

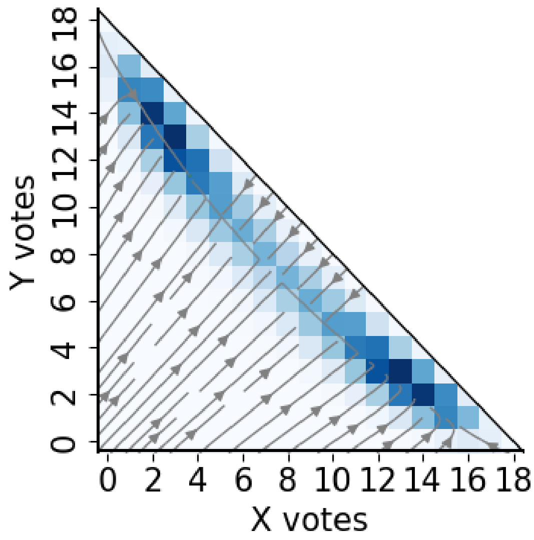

(b) Simulations.

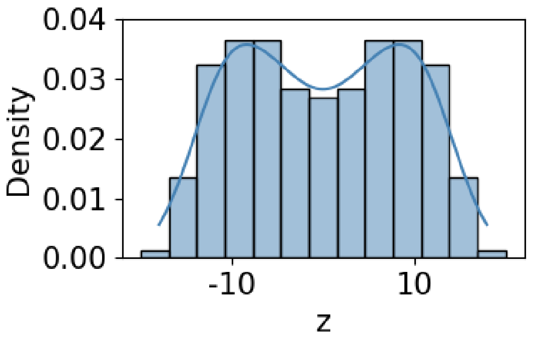

(c)

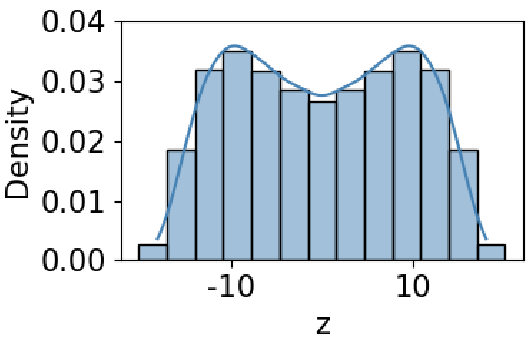

(d)

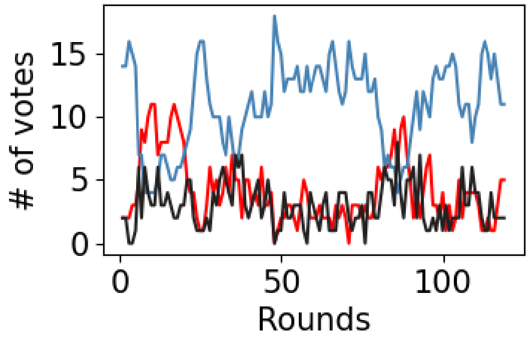

(e)

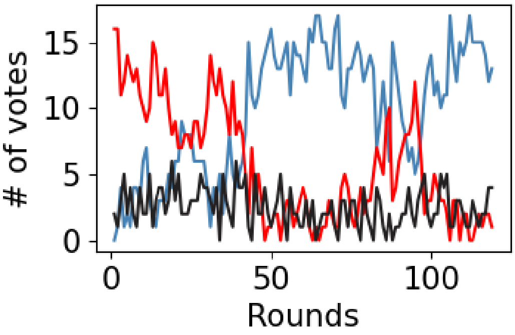

(f)

Figure SI.23: (a) A heat map of the data of an experiment with $N = 18$ participants. (b) A heat map generated from a simulated time course with the flow field superimposed atop. Parameters for the simulations are fitted from experimental data. (c-d) One-dimensional projections of the stationary probability distributions onto $Z = (X - Y)$ (see SM Sections 1 and 6 for details) for experimental (c) and simulated data (d). The Gaussian kernel density estimation is shown atop the histograms. (e-f) Time courses of the experimental (e) and simulated data (f). Votes for option X, option Y and abstentions are represented in blue, red and black, respectively.

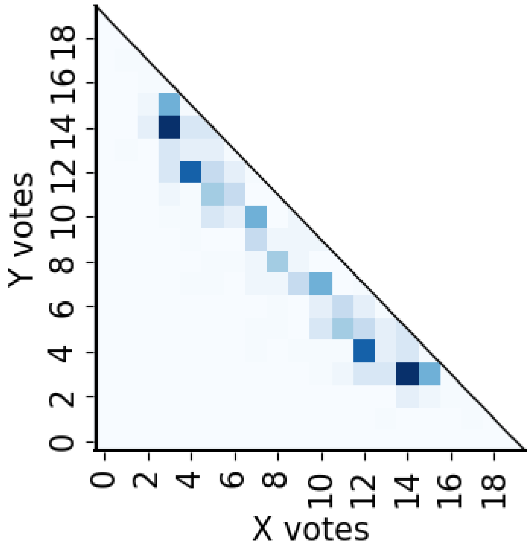

(a) Experimental data.

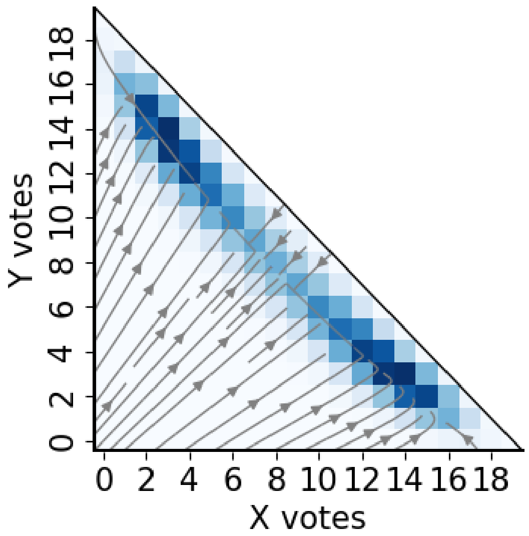

(b) Simulations.

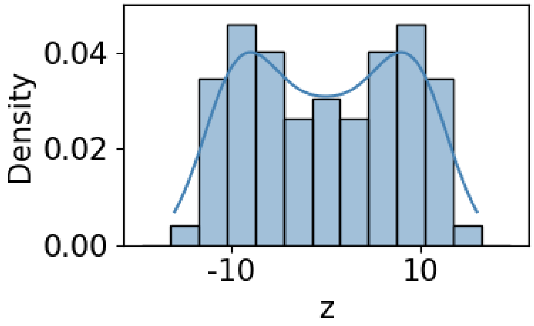

(c)

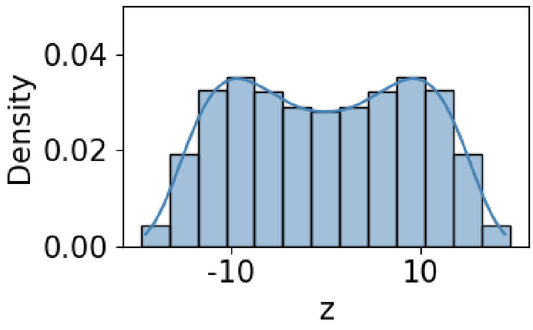

(d)

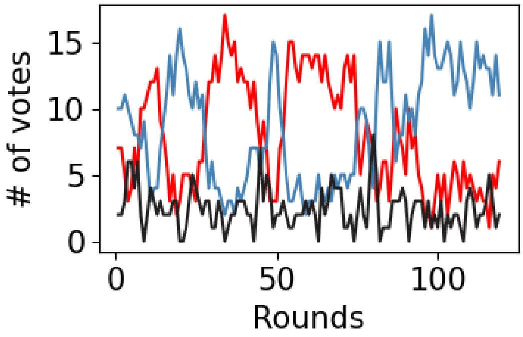

(e)

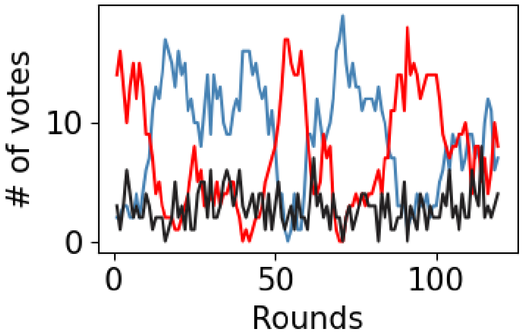

(f)

Figure SI.24: (a) A heat map of the data of for the first experiment with $N = 19$ participants. (b) A heat map generated from a simulated time course with the flow field superimposed atop. Parameters for the simulations are fitted from experimental data. (c-d) One-dimensional projections of the stationary probability distributions onto $Z = (X - Y)$ (see SM Sections 1 and 6 for details) for experimental (c) and simulated data (d). The Gaussian kernel density estimation is shown atop the histograms. (e-f) Time courses of the experimental (e) and simulated data (f). Votes for option X, option Y and abstentions are represented in blue, red and black, respectively.

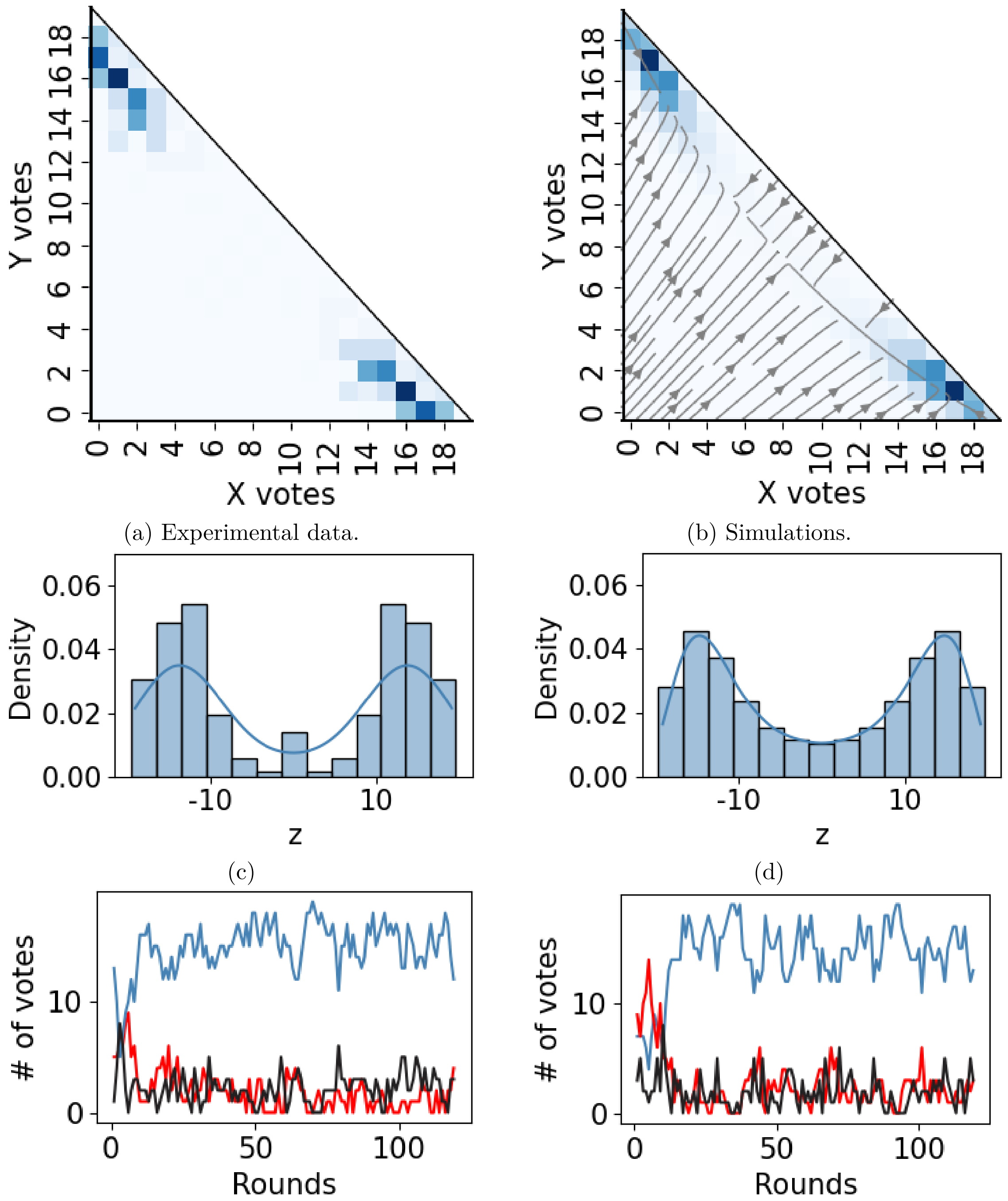

(a) Experimental data.

(b) Simulations.

(c)

(d)

(e)

(f)

Figure SI.25: (a) A heat map of the data of the second experiment with $N = 19$ participants. (b) A heat map generated from a simulated time course with the flow field superimposed atop. Parameters for the simulations are fitted from experimental data. (c-d) One-dimensional projections of the stationary probability distributions onto $Z = (X - Y)$ (see SM Sections 1 and 6 for details) for experimental (c) and simulated data (d). The Gaussian kernel density estimation is shown atop the histograms. (e-f) Time courses of the experimental (e) and simulated data (f). Votes for option X, option Y and abstentions are represented in blue, red and black, respectively.

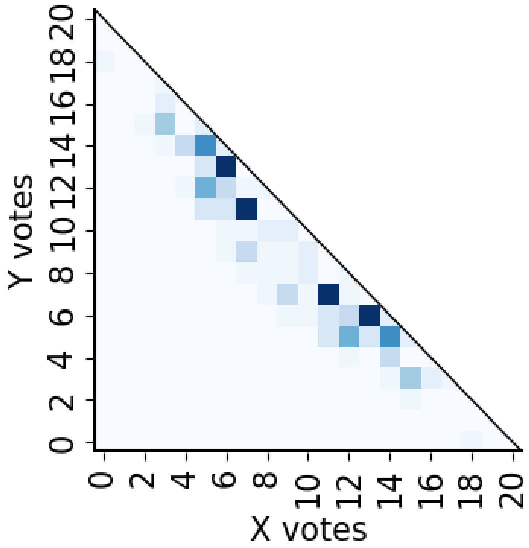

(a) Experimental data.

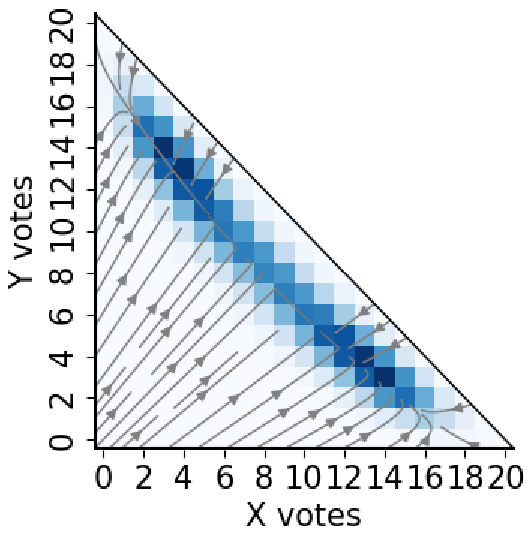

(b) Simulations.

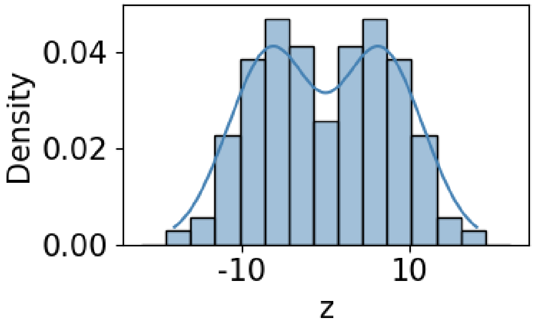

(c)

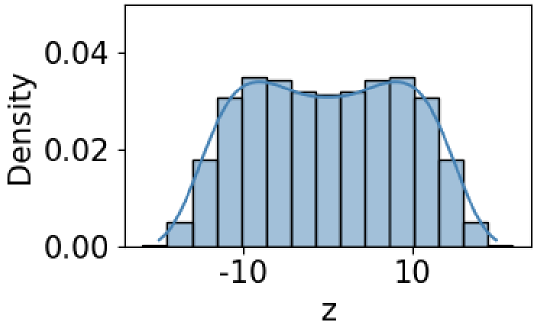

(d)

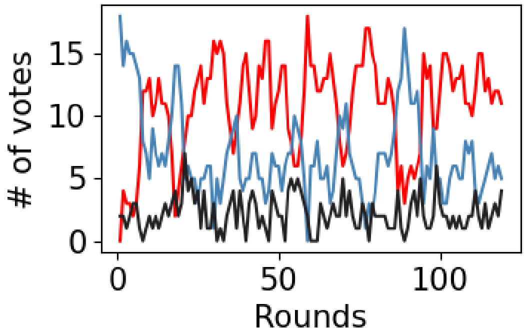

(e)

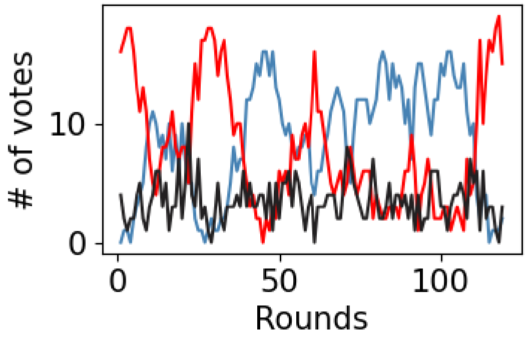

(f)

Figure SI.26: (a) A heat map of the data of an experiment with $N = 20$ participants. (b) A heat map generated from a simulated time course with the flow field superimposed atop. Parameters for the simulations are fitted from experimental data. (c-d) One-dimensional projections of the stationary probability distributions onto $Z = (X - Y)$ (see SM Sections 1 and 6 for details) for experimental (c) and simulated data (d). The Gaussian kernel density estimation is shown atop the histograms. (e-f) Time courses of the experimental (e) and simulated data (f). Votes for option X, option Y and abstentions are represented in blue, red and black, respectively.

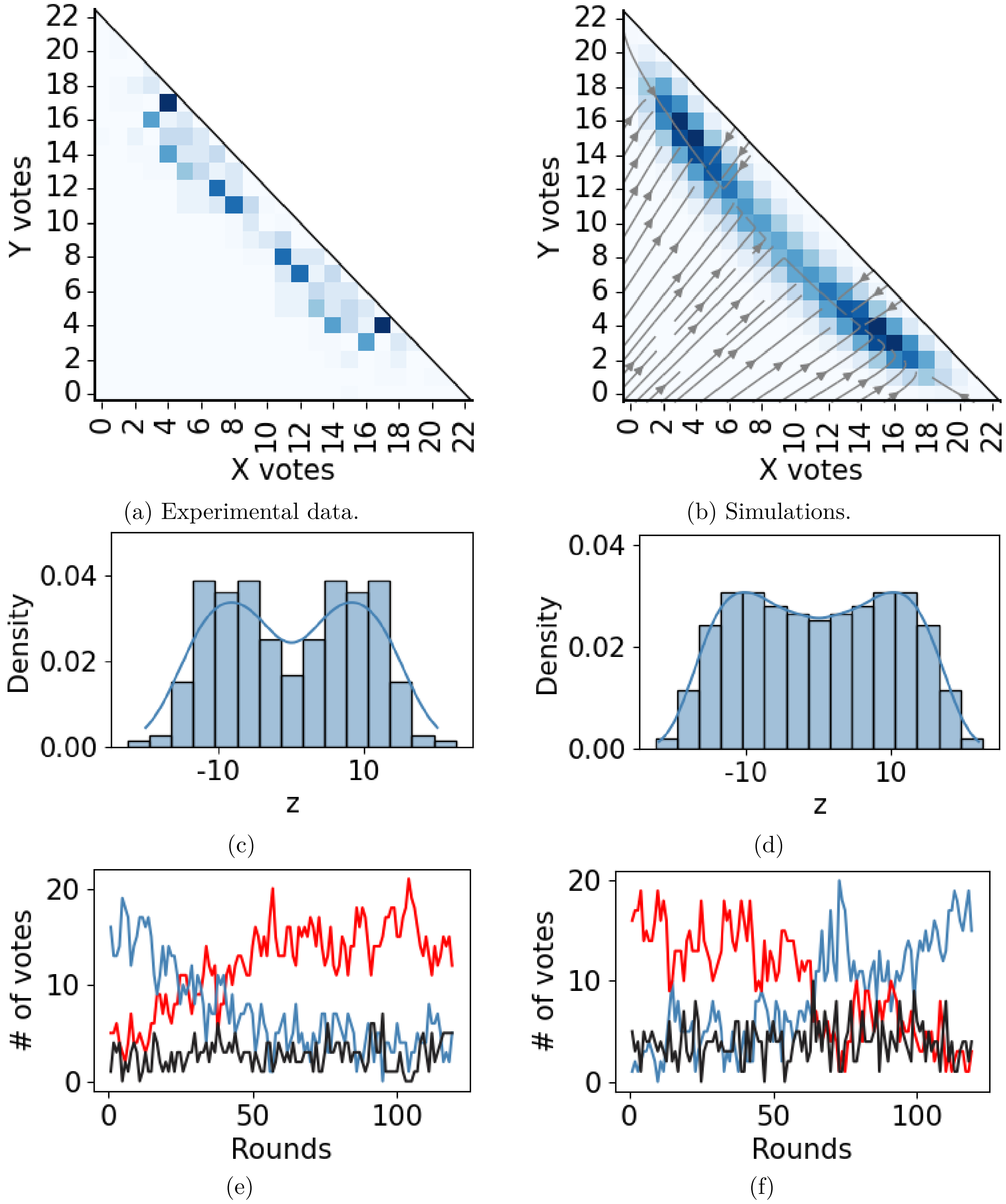

(a) Experimental data. (b) Simulations.

(c) (d)

(e) (f)

Figure SI.27: (a) A heat map of the data of an experiment with $N = 22$ participants. (b) A heat map generated from a simulated time course with the flow field superimposed atop. Parameters for the simulations are fitted from experimental data. (c-d) One-dimensional projections of the stationary probability distributions onto $Z = (X - Y)$ (see SM Sections 1 and 6 for details) for experimental (c) and simulated data (d). The Gaussian kernel density estimation is shown atop the histograms. (e-f) Time courses of the experimental (e) and simulated data (f). Votes for option X, option Y and abstentions are represented in blue, red and black, respectively.

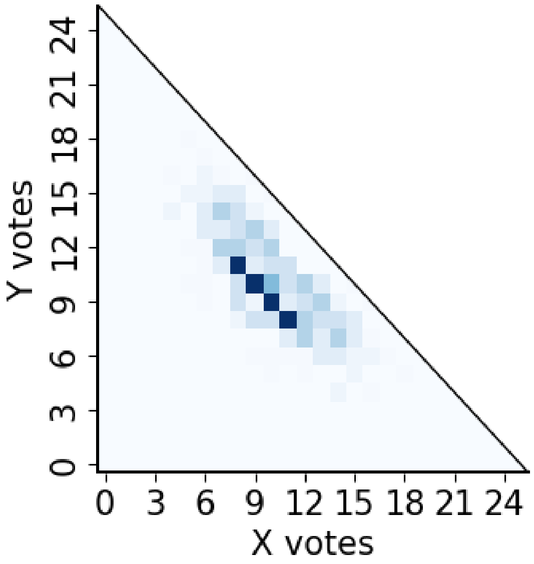

(a) Experimental data.

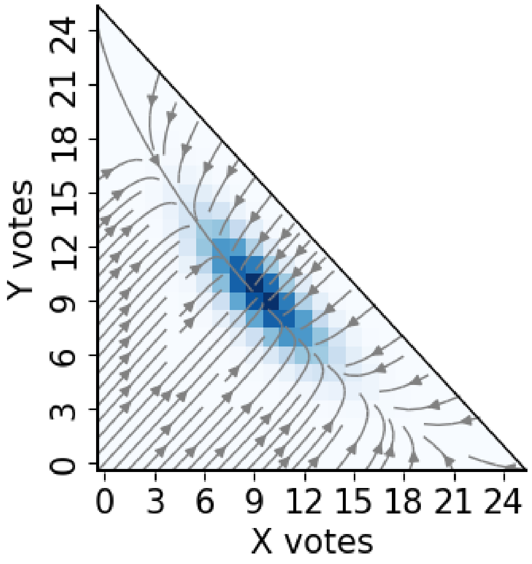

(b) Simulations.

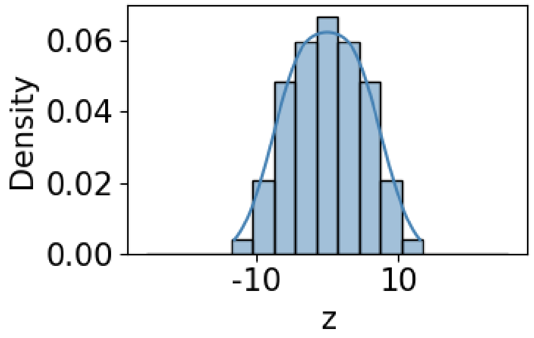

(c)

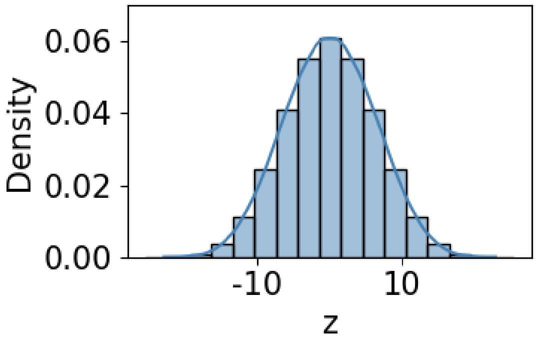

(d)

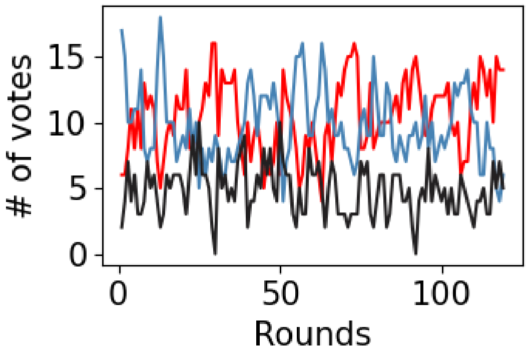

(e)

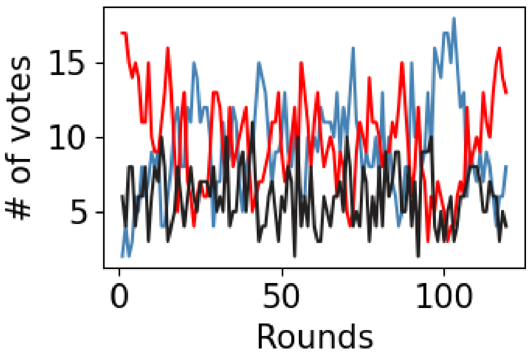

(f)

Figure SI.28: (a) A heat map of the data of an experiment with $N = 25$ participants. (b) A heat map generated from a simulated time course with the flow field superimposed atop. Parameters for the simulations are fitted from experimental data. (c-d) One-dimensional projections of the stationary probability distributions onto $Z = (X - Y)$ (see SM Sections 1 and 6 for details) for experimental (c) and simulated data (d). The Gaussian kernel density estimation is shown atop the histograms. (e-f) Time courses of the experimental (e) and simulated data (f). Votes for option X, option Y and abstentions are represented in blue, red and black, respectively.

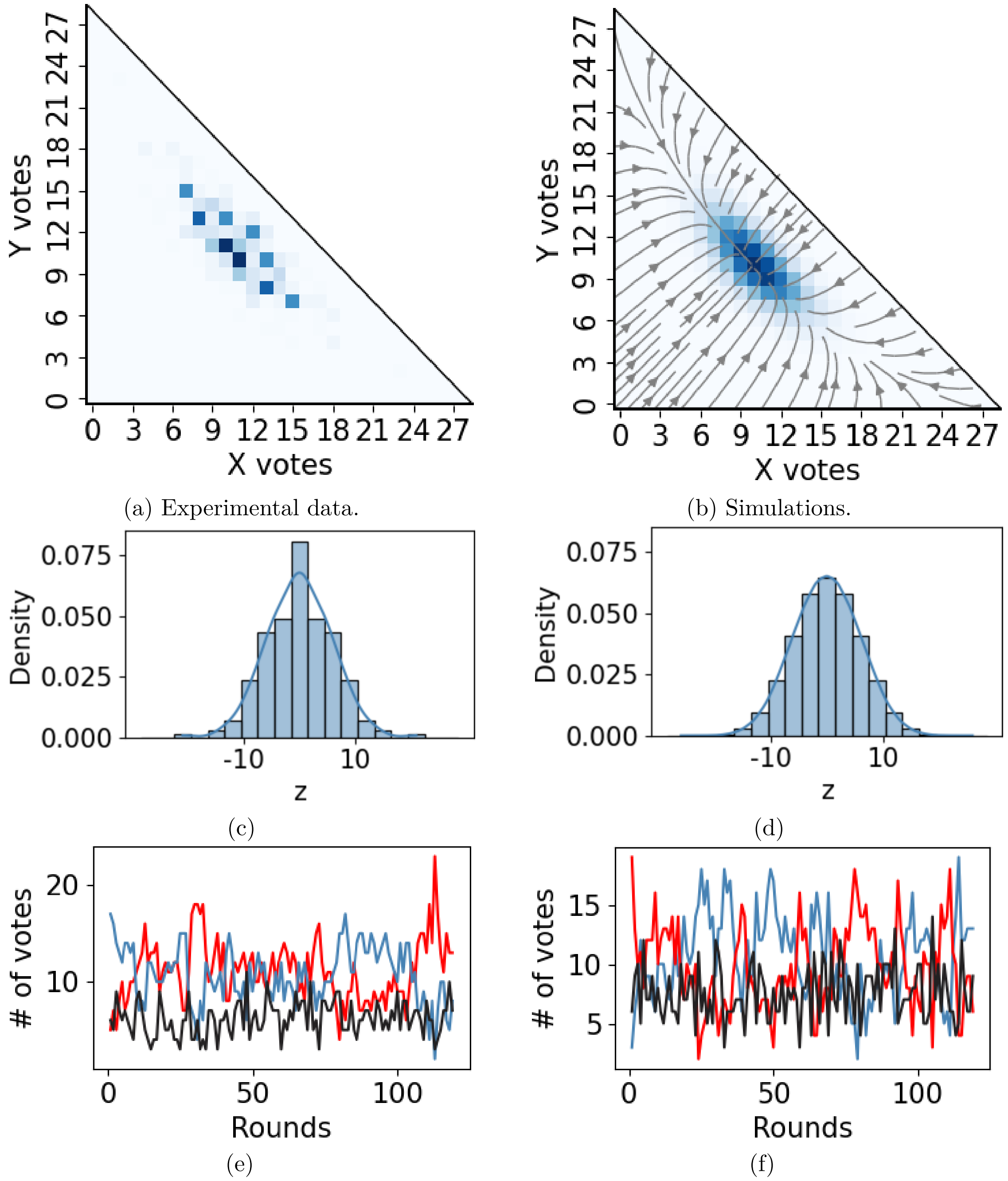

(a) Experimental data. (b) Simulations. (c) (d) (e) (f)

Figure SI.29: (a) A heat map of the data of an experiment with $N = 28$ participants. (b) A heat map generated from a simulated time course with the flow field superimposed atop. Parameters for the simulations are fitted from experimental data. (c-d) One-dimensional projections of the stationary probability distributions onto $Z = (X - Y)$ (see SM Sections 1 and 6 for details) for experimental (c) and simulated data (d). The Gaussian kernel density estimation is shown atop the histograms. (e-f) Time courses of the experimental (e) and simulated data (f). Votes for option X, option Y and abstentions are represented in blue, red and black, respectively.

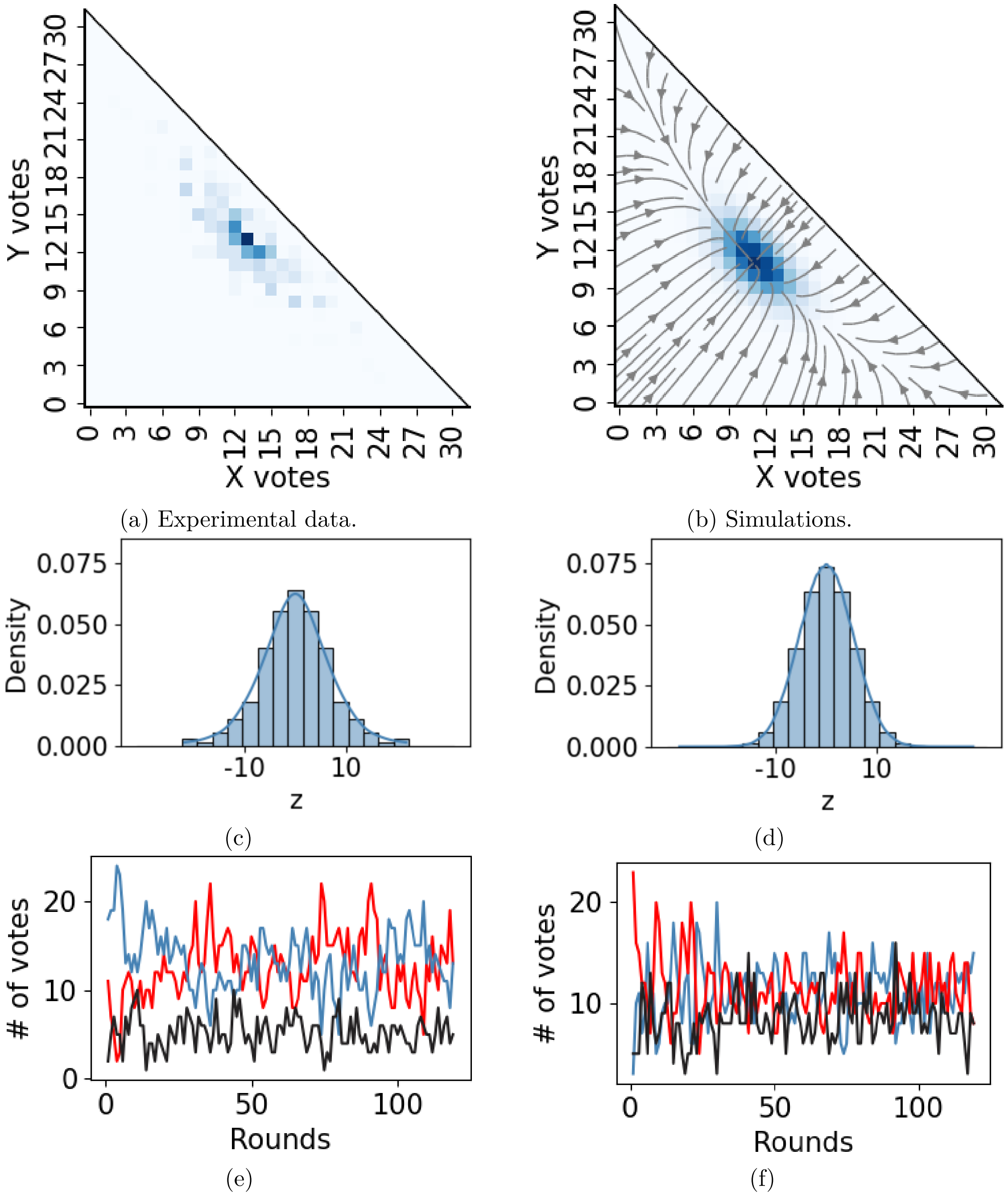

(a) Experimental data.

(b) Simulations.

(c)

(d)

(e)

(f)

Figure SI.30: (a) A heat map of the data of an experiment with $N = 31$ participants. (b) A heat map generated from a simulated time course with the flow field superimposed atop. Parameters for the simulations are fitted from experimental data. (c-d) One-dimensional projections of the stationary probability distributions onto $Z = (X - Y)$ (see SM Sections 1 and 6 for details) for experimental (c) and simulated data (d). The Gaussian kernel density estimation is shown atop the histograms. (e-f) Time courses of the experimental (e) and simulated data (f). Votes for option X, option Y and abstentions are represented in blue, red and black, respectively.

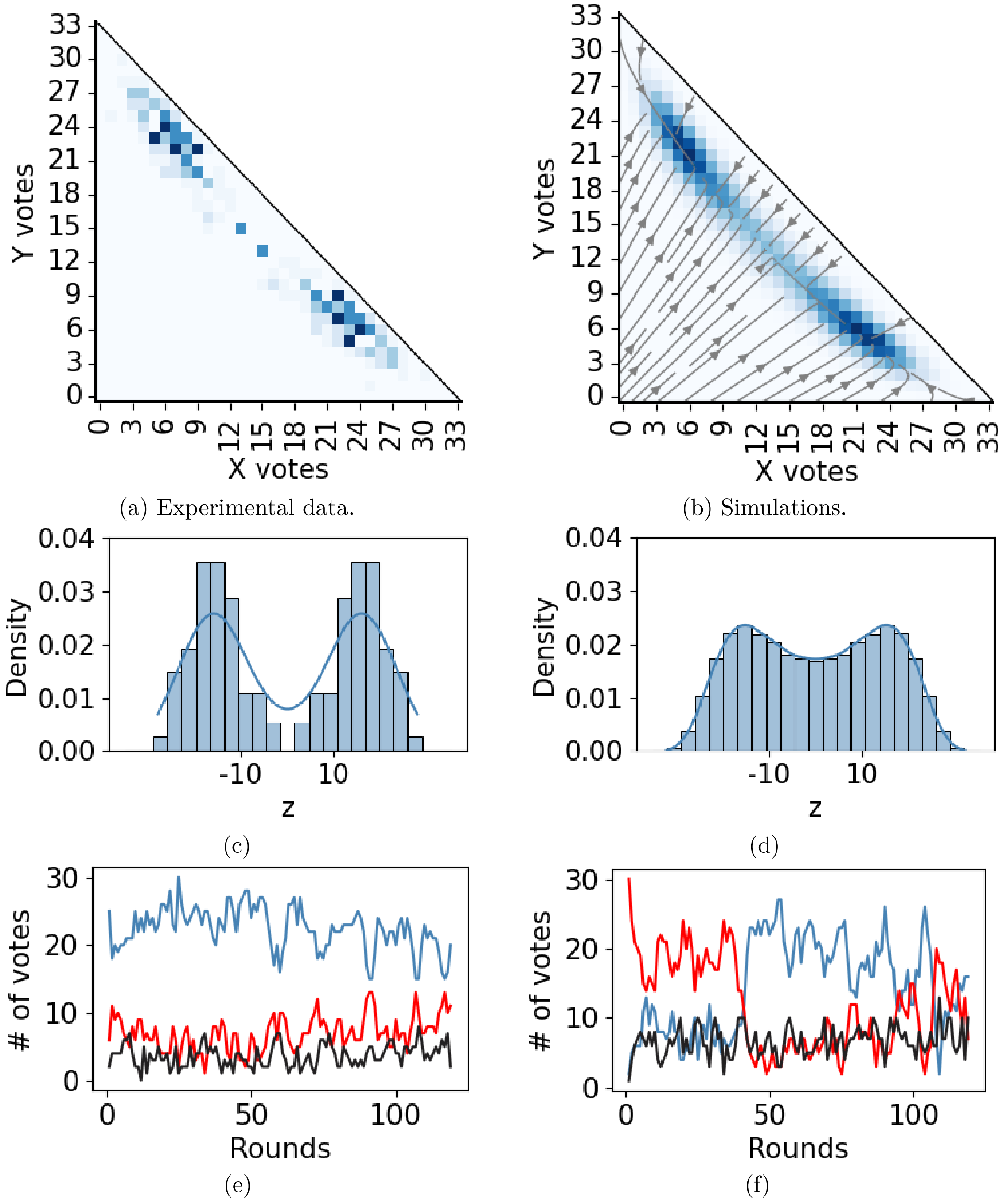

(a) Experimental data.

(b) Simulations.

(c)

(d)

(e)

(f)

Figure SI.31: (a) A heat map of the data of an experiment with $N = 33$ participants. (b) A heat map generated from a simulated time course with the flow field superimposed atop. Parameters for the simulations are fitted from experimental data. (c-d) One-dimensional projections of the stationary probability distributions onto $Z = (X - Y)$ (see SM Sections 1 and 6 for details) for experimental (c) and simulated data (d). The Gaussian kernel density estimation is shown atop the histograms. (e-f) Time courses of the experimental (e) and simulated data (f). Votes for option X, option Y and abstentions are represented in blue, red and black, respectively.

SI.6.4 No abstention experiments

Following the procedures described in Section SI.5, we performed eight experiments without the option to abstain. Fig. SI.32 shows the time courses of the experiments.

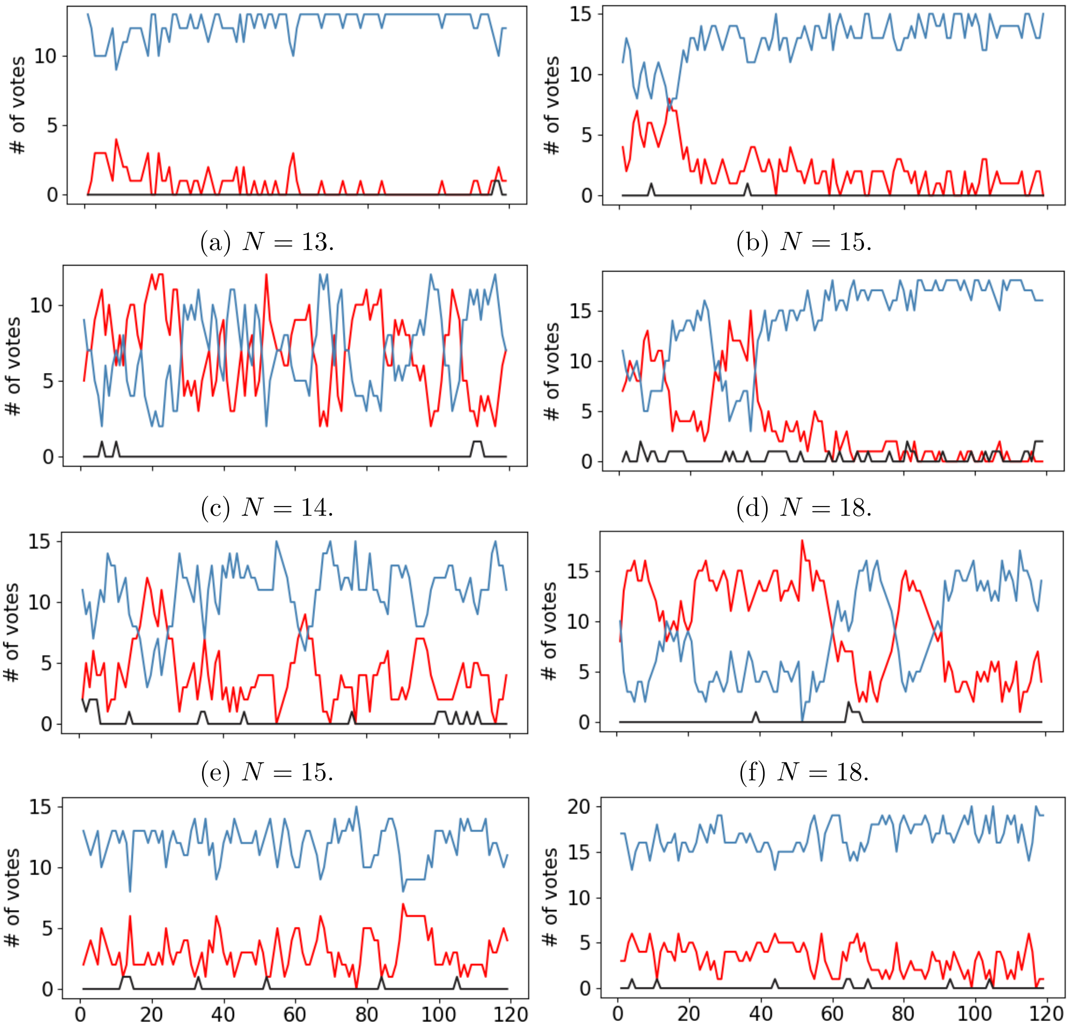

(a) $N = 13$. (b) $N = 15$.

(c) $N = 14$. (d) $N = 18$.

(e) $N = 15$. (f) $N = 18$.

(g) $N = 15$. (h) $N = 20$.

Figure SI.32: Time series of the experiments without the option to abstain. The number of votes for option X, Y and timeouts is represented in red, blue and black, respectively. Timeouts do not significantly affect the experiments. No strong consensus forms in (c). A strong consensus is observed in the remaining experiments with (a), (b), (g) and (h) not showing switches, (d) and (e) showing brief switches, and (f) showing a definite switch in the majority.

SI.7 Nucleosome modification model analysis

The nucleosome modification model [Dodd et al., 2007] is another example of the applicability of our model on a completely different physical scale. The model describes the modifications of nucleosomes by recruitment of modifying and unmodifying enzymes from their neighbours or in a 'recruitment-independent' (spontaneous) manner. In the model, nucleosomes can be acetylated (A), unmodified (U) or methylated (M), hence A and M represent active stances, while U is a neutral stance.

Dodd et al.'s model [Dodd et al., 2007]considers a population of $N = 60$ nucleosomes placed on a line without periodic boundary conditions. The computational model comprises two steps:

1. **Nucleosome selection.** At each time step, a random nucleosome, n_1, is selected among the N nucleosomes in the population to be modified.
2. **Modification.** Once a nucleosome is selected, one of the following happens:
   (a) *Recruitment conversion.* With probability p a recruitment conversion is attempted. A second nucleosome, n_2, is selected from the neighbourhood of n_1. If n_2 is A or M, the stance of n_1 is changed one step closer to that of n_2 (e.g., if $n_2 = A$, then the stance of n_1 undergoes either the transition $U \to A$ or $M \to U$). No changes happen if $n_2 = n_1$ or if $n_2 = U$.
   (b) *Independent conversion.* With probability $1 - p$ an independent change is performed. If $n_1 = A$ then that nucleosome is converted to stance U with probability 1/3 and stays in stance A otherwise. Similarly, if $n_1 = M$ that nucleosome is converted to stance U with probability 1/3 and stays in stance M otherwise. However if $n_1 = U$ then it is converted to stance M with probability 1/3, to stance A with probability 1/3 and stays in stance U with the remaining probability (1/3).

Fig. SI.33 shows the system's behaviour as we vary the neighbourhood size on each side of a nucleosome, $\mathcal{N}$, and the recruitment probability, p. Note that, given the absence of periodic boundary conditions, the first nucleosome can only be affected by the last nucleosome if $\mathcal{N} = 60$. In Fig. SI.33 we observe two interesting phenomena as p increases. Firstly, the alignment (or the difference in proportions of methylated and acetylated nucleosomes in the population in the favourable system states) is approximately zero until $p \approx 0.52$ when the system transitions from a disorder to an ordered state, becoming more polarised as p increases. Secondly, the system undergoes a phase transition in which the mean switching time quickly diverges as p becomes larger than ≈ 0.75 (indicated by the dark blue region in Fig. SI.33 (b)). For $p \leq 0.52$ the system is disordered (unimodal) and the curvature of the data on the simplex is difficult to estimate accurately. In terms of the neighbourhood size, the manifolds are curved for larger neighbourhood sizes when the system is well-mixed, turning into straight

lines as the neighbourhood becomes small and interactions are local.

Note that this is a discrete-time model for a symmetric system (acetylated/methylated) with a neutral stance (unmodified) and recruitment dynamics, which holds similarities to our general model. However, this model has a strong spatial component absent in our modelling framework. Nucleosomes are static and can only interact within a predefined neighbourhood of their location. Nonetheless, simulations of this model show that the same manifold dynamics are observed in this case, suggesting a wider range of applicability of our model than expected from our assumptions.

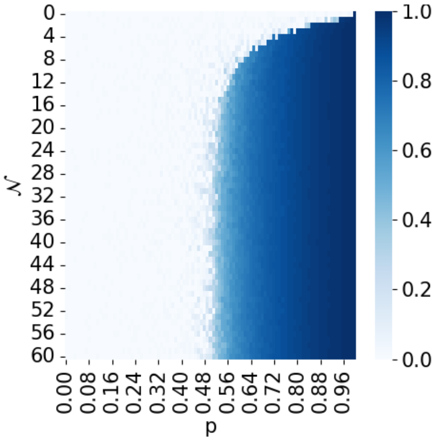

(a)

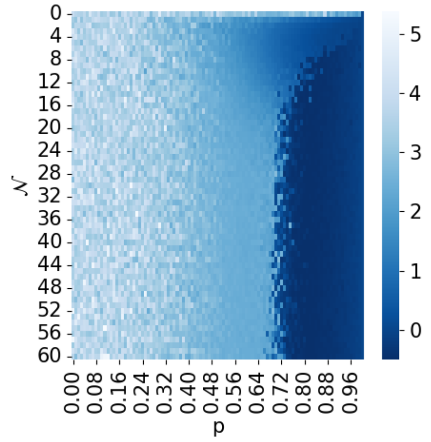

(b)

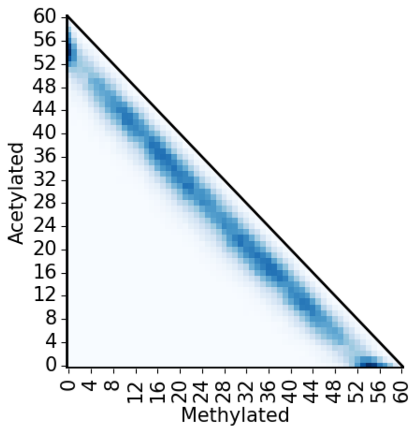

(c) $\mathcal{N} = 2$, $p = 0.83$.

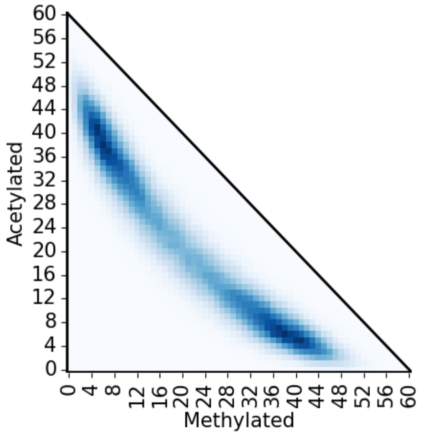

(d) $\mathcal{N} = 20$, $p = 0.55$.

Figure SI.33: Consensus switching between chromatin states follows a curved slow manifold, indicating that transitions occur through intermediate states of increased neutrality rather than direct flips between active states.(a-b) Phase plot analysis of Dodd et al. [2007]'s model for the neighbourhood size, $\mathcal{N}$, and recruitment probability, p. (a) The polarization (consensus) at the favourable states of the system. (b) The curvature, γ, of the lower dimensional manifold obtained by fitting the curve $1 - x - y - \gamma xy = \beta$ to the simulation data for (x, y) corresponding to the proportion of methylated and acetylated nucleosomes, respectively. We note a transition from disorder to order and straight to curved manifolds as p increases. (c-d) Heat maps of the states visited by the system (dark colours indicate frequency) for two choices of $\mathcal{N}$ and p, giving straight (c) and curved (d) manifolds.

Bibliography

H. C. Berg, editor. *E. coli in Motion*. Springer New York, 2004. ISBN 9780387216386.

T. Biancalani, L. Dyson, and A. J. McKane. Noise-induced bistable states and their mean switching time in foraging colonies. *Physical Review Letters*, 112(3), 01 2014.

N. W. Bode, D. W. Franks, and A. Jamie Wood. Making noise: Emergent stochasticity in collective motion. *Journal of Theoretical Biology*, 267(3):292–299, Dec. 2010. ISSN 0022-5193.

M. R. Bonyadi and Z. Michalewicz. Particle swarm optimization for single objective continuous space problems: A review. *Evolutionary Computation*, 25(1):1–54, Mar. 2017. ISSN 1530-9304.

J. Buhl, D. J. Sumpter, I. D. Couzin, J. J. Hale, E. Despland, E. R. Miller, and S. J. Simpson. From disorder to order in marching locusts. *Science*, 312(5778):1402–1406, 2006.

D. L. Chen, M. Schonger, and C. Wickens. otree – an open-source platform for laboratory, online, and field experiments. *Journal of Behavioral and Experimental Finance*, 9:88–97, 2016. ISSN 2214-6350.

E. A. Codling, M. J. Plank, and S. Benhamou. Random walk models in biology. *Journal of The Royal Society Interface*, 5(25):813–834, apr 2008.

G. W. A. Constable, T. Rogers, A. J. McKane, and C. E. Tarnita. Demographic noise can reverse the direction of deterministic selection. *Proceedings of the National Academy of Sciences*, 113(32), July 2016. ISSN 1091-6490.

A. Czirók, A.-L. Barabási, and T. Vicsek. Collective motion of self-propelled particles: Kinetic phase transition in one dimension. *Physical Review Letters*, 82(1):209–212, 01 1999.

M. Dai, T. Gao, Y. Lu, Y. Zheng, and J. Duan. Detecting the maximum likelihood transition path from data of stochastic dynamical systems. *Chaos: An Interdisciplinary Journal of Nonlinear Science*, 30(11), Nov. 2020. ISSN 1089-7682.

I. B. Dodd, M. A. Micheelsen, K. Sneppen, and G. Thon. Theoretical analysis of epigenetic cell memory by nucleosome modification. *Cell*, 129(4):813–822, May 2007. ISSN 0092-8674.

L. Dyson, C. A. Yates, J. Buhl, and A. J. McKane. Onset of collective motion in locusts is captured by a minimal model. *Physical Review E*, 92(5):052708, 2015.

R. Erban, J. Chapman, and P. Maini. A practical guide to stochastic simulations of reaction-diffusion processes, 2007.

C. Gardiner. *Handbook of stochastic methods for physics, chemistry, and the natural sciences.* Springer, second edition, 1985.

E. Gavagnin, J. P. Owen, and C. A. Yates. Pair correlation functions for identifying spatial correlation in discrete domains. *Physical review special topics.*, 97(6):062104, 2018. ISSN 1554-9178.

G. Grimmett and D. Stirzaker. *Probability and random processes.* Oxford University Press, London, England, 4 edition, July 2020.

G. S. Katzenberger. *Solutions of a stochastic differential equation forced onto a manifold by a large drift.* The University of Wisconsin-Madison, 1990.

J. Kennedy. *Swarm Intelligence*, pages 187–219. Springer US, Boston, MA, 2006. ISBN 978-0-387-27705-9.

J. Kennedy and R. Eberhart. Particle swarm optimization. In *Proceedings of ICNN'95 - International Conference on Neural Networks*, volume 4, pages 1942–1948 vol.4, 1995.

D. Levin and Y. Peres. *Markov Chains and Mixing Times.* American Mathematical Society, Oct. 2017. ISBN 9781470442323.

K. Martens, L. Angelani, R. Di Leonardo, and L. Bocquet. Probability distributions for the run-and-tumble bacterial dynamics: An analogy to the lorentz model. *The European Physical Journal E*, 35(9), Sept. 2012. ISSN 1292-895X.

K. Minors, T. Rogers, and C. A. Yates. Noise-driven bias in the non-local voter model. *EPL (Europhysics Letters)*, 122(1):10004, Apr. 2018.

T. L. Parsons and T. Rogers. Dimension reduction for stochastic dynamical systems forced onto a manifold by large drift: a constructive approach with examples from theoretical biology. *Journal of Physics A: Mathematical and Theoretical*, 50(41):415601, 09 2017.

Prolific. www.prolific.com, 2014. URL `https://www.prolific.com`. Accessed: 2023-08-12.

G. Rosser, A. G. Fletcher, D. A. Wilkinson, J. A. de Beyer, C. A. Yates, J. P. Armitage, P. K. Maini, and R. E. Baker. Novel methods for analysing bacterial tracks reveal persistence in rhodobacter sphaeroides. *PLoS Computational Biology*, 9(10):e1003276, Oct. 2013. ISSN 1553-7358.

Y. Rubner, C. Tomasi, and L. Guibas. A metric for distributions with applications to

image databases. In *Sixth International Conference on Computer Vision (IEEE Cat. No.98CH36271)*, pages 59–66, 1998.

Y. Sowa and R. M. Berry. Bacterial flagellar motor. *Quarterly Reviews of Biophysics*, 41(2): 103–132, 2008.

P. Stoica and Y. Selen. Model-order selection. *IEEE Signal Processing Magazine*, 21(4): 36–47, July 2004.

N. Van Kampen. Chapter 10 - The expansion of the master equation. In *Stochastic Processes in Physics and Chemistry (Third Edition)*, North-Holland Personal Library, pages 244–272. Elsevier, Amsterdam, third edition edition, 2007.

C. Yates, R. Erban, C. Escudero, I. Couzin, J. Buhl, I. Kevrekidis, P. Maini, and D. Sumpter. Inherent noise can facilitate coherence in collective swarm motion. *Proc. Natl. Acad. Sci. USA*, 106(14):5464–5469, Mar 2009. doi: 10.1073/pnas.0811195106. URL `http://dx.doi.org/10.1073/pnas.0811195106`.

C. Yates, R. Baker, R. Erban, and P. Maini. Refining self-propelled particle models for collective behaviour. *The Canadian Applied Mathematics Quarterly*, 18(3):299–350, 2011. ISSN 1073-1849.

K. Younge, B. Johnston, C. Christenson, A. Bohara, J. Jacobson, N. M. Butler, and P. Saulnier. The use of radial distribution and pair-correlation functions to analyze and describe biological aggregations: Radial distribution analysis of swarms. *Limnology and Oceanography: Methods*, 4(10):382–391, Nov. 2006. ISSN 1541-5856.

Y. Zheng and X. Sun. Governing equations for probability densities of stochastic differential equations with discrete time delays. *Discrete and Continuous Dynamical Systems - B*, 22 (9):3615–3628, 2017. ISSN 1531-3492.

Y. Zheng, F. Yang, J. Duan, X. Sun, L. Fu, and J. Kurths. The maximum likelihood climate change for global warming under the influence of greenhouse effect and Lévy noise. *Chaos: An Interdisciplinary Journal of Nonlinear Science*, 30(1):013132, 01 2020. ISSN 1054-1500.
